# Organocatalytic asymmetric chlorinative dearomatization of naphthols†
†Electronic supplementary information (ESI) available: Experimental procedures and analysis data for the new compounds. CCDC 1048128 ((*R*)-**2t**) and 1048302 ((*R*)-**2d**). For ESI and crystallographic data in CIF or other electronic format see DOI: 10.1039/c5sc00494b


**DOI:** 10.1039/c5sc00494b

**Published:** 2015-04-27

**Authors:** Qin Yin, Shou-Guo Wang, Xiao-Wei Liang, De-Wei Gao, Jun Zheng, Shu-Li You

**Affiliations:** a State Key Laboratory of Organometallic Chemistry , Shanghai Institute of Organic Chemistry , Chinese Academy of Sciences , 345 Lingling Lu , Shanghai 200032 , China . Email: slyou@sioc.ac.cn ; Fax: +86-21-5492-5087

## Abstract

An organocatalytic asymmetric chlorinative dearomatization of naphthols was realized for the first time, providing chiral naphthalenones with a Cl-containing all-substituted stereocenter in excellent yields and enantioselectivity (up to 97% yield and 96% ee). The reaction features mild reaction conditions, good tolerance of diverse functional groups and simple reaction operation.

## Abstract 2

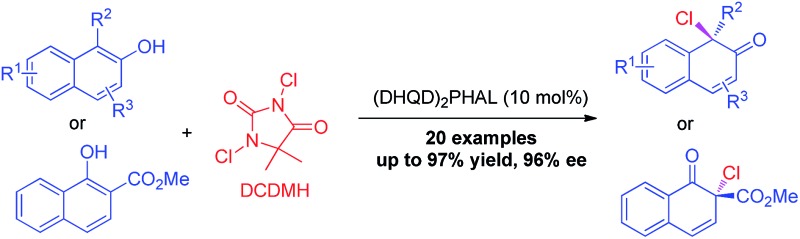
A highly enantioselective chlorinative dearomatization of 1-naphthol and 2-naphthols was realized for the first time, providing chiral naphthalenones with a Cl-containing all-substituted stereocenter in excellent yields and enantioselectivity (up to 97% yield and 96% ee).

## 

Phenol and its derivatives are readily accessible chemical feedstocks and are widely utilized in chemical synthesis.^1^ Among the versatile transformations, the catalytic asymmetric dearomatization (CADA) reaction^2^ of phenol derivatives offers a facile and straightforward route to access chiral cyclic enones with one quaternary carbon stereogenic center. Therefore, development of the CADA reaction of phenol derivatives has received increasing attention recently.^3^ Strategies for the direct catalytic asymmetric dearomatization of phenols, including hypervalent iodine^4^ or transition metal-catalyzed^5^ oxidation, transition metal-catalyzed allylic alkylation^6^ or arylation,^7^ and chiral phosphoric acid catalyzed amination,^8^ have been elegantly unveiled.^9^ Very recently, Toste and co-workers reported a highly enantioselective dearomative fluorination of phenols by chiral anion phase-transfer catalysis.^10^ Inspired by these pioneering works, we envisaged that the asymmetric chlorinative dearomatization of phenols *via* homogeneous catalysis might be possible, providing interesting products with a C–Cl bond-containing chiral center.^11^ However, compared with electrophilic fluorination reagents such as Selectfluor, electrophilic chlorination reagents such as *N*-chlorosuccinimide (NCS) and DCDMH (1,3-dichloro-5,5-dimethylhydantoin) have much higher electrophilic reactivity, which may cause a significant amount of background reaction or undesired reactions such as electrophilic aromatic substitution at the *ortho* or *para*-position (the problem of regioselectivity). In addition, the construction of a Cl-containing all-substituted stereocenter with high enantioselectivity *via* dearomatization of phenols remains underexplored. To test our hypothesis, commercially available cinchonine derivatives such as (DHQD)_2_PHAL were chosen as chiral catalysts since they are privileged catalysts for the asymmetric halofunctionalization of alkenes.^12^,^13^ After extensive preliminary investigation of substituted phenols, we found that naphthols are suitable substrates for the chlorinative dearomatization process.^14^ Herein, we report such a highly enantioselective dearomative chlorination of naphthols under catalysis by (DHQD)_2_PHAL, providing an efficient synthesis of chiral naphthalenones with an α-Cl-containing all-substituted stereocenter (Scheme 1).^15^

**Scheme 1:**
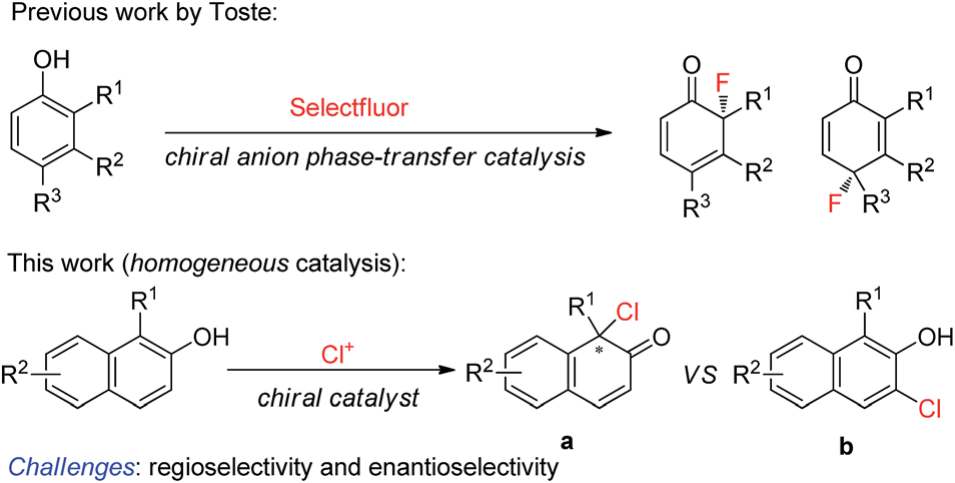
Asymmetric chlorination of naphthol derivatives *via* homogeneous catalysis.

We commenced our studies by testing the reactions between commercially available substrate **1a** and 1,3-dichloro-5,5-dimethylhydantoin (DCDMH) in the presence of 10 mol% (DHQD)_2_PHAL.^16^ Firstly, various solvents were surveyed at room temperature. The dearomative product **2a** could be obtained in 94% yield in toluene with encouraging enantioselectivity observed (52% ee, entry 1). Further screening of chlorine-containing solvents revealed that CCl_4_ could give comparable results, affording **2a** in 54% ee (entries 2–5). To our surprise, the screening of other solvents revealed that CS_2_ gave the best results and **2a** could be produced in 90% yield with 62% ee with a prolonged reaction time (entries 6–8). However, the enantioselectivity of **2a** was only slightly elevated from 62% ee to 64% ee when the reaction was carried out at –30 °C in CS_2_ (entry 9). Gratifyingly, a significant increase in enantioselectivity could be achieved when the reaction was performed in toluene at a decreased temperature. **2a** was obtained in 95% yield with 92% ee by carrying out the reaction at –78 °C (entry 11). Notably, by changing the catalyst from (DHQD)_2_PHAL to (DHQ)_2_PHAL, **2a** with 90% ee, with the opposite configuration, could be obtained in an almost quantitative yield (entry 12). A decrease in the catalyst loading from 10 mol% to 2 mol% led to a prolonged reaction time, however, the yield and enantioselectivity of **2a** remained at an excellent level (98% yield, 90% ee, entry 13) (Table 1).

**Table 1:** Evaluation of reaction conditions

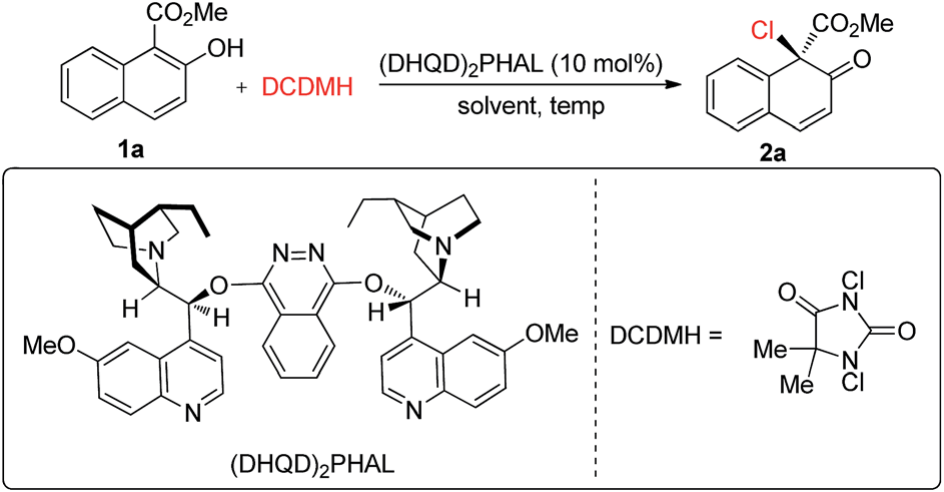					
Entry^*a*^	Solvent	Temp (°C)	Time (h)	Yield^*b*^ (%)	Ee^*c*^ (%)
1	Toluene	Rt	1	94	52
2	DCM	Rt	1	92	36
3	DCE	Rt	1	93	25
4	CHCl_3_	Rt	1	92	34
5	CCl_4_	Rt	1	92	54
6	Hexane	Rt	1	89	32
7	THF	Rt	1	88	12
8	CS_2_	Rt	4	90	62
9	CS_2_	–30	24	91	64
10	Toluene	–30	8	94	75
11	Toluene	–78	10	95	92
12^*d*^	Toluene	–78	10	94	–90
13^*e*^	Toluene	–78	31	98	90

^*a*^Reactions were performed with **1a** (0.1 mmol), DCDMH (0.12 mmol) and 10 mol% of (DHQD)_2_PHAL at rt in an open flask.

^*b*^Isolated yield.

^*c*^Determined by HPLC analysis.

^*d*^10 mol% of (DHQ)_2_PHAL was utilized.

^*e*^2 mol% of (DHQD)_2_PHAL was utilized.

Under the optimized reaction conditions, 2-naphthols with different substituents were synthesized to test the generality of this asymmetric chlorination process (Scheme 2). Firstly, the substituent effect of the ester group (Me, Et, allyl) was evaluated and, in all cases, excellent yields were achieved. With the increase in steric hindrance from a methyl, to an ethyl, to an allyl group, the enantioselectivity of the corresponding products **2a–2c** showed a decreasing trend. However, the levels were still excellent (**2a–2c**, 91–95% yields, 86–92% ee). The substituent effect on the core of 2-hydroxy-1-naphthoate was next investigated. Electron-withdrawing groups such as 6-Br and 6-CN, or aryl groups such as Ph, 3,5-(Me)_2_C_6_H_3_ and 4-F-C_6_H_4_ were well tolerated. The corresponding products were all obtained in excellent yields and enantioselectivity (**2d–2h**, 80–88% yields, 93–96% ee). Electron-donating groups such as 6-Me and 6-phenethyl were also well tolerated and the corresponding products **2i** and **2j** were obtained in 87% yield, 93% ee and 90% yield, 94% ee, respectively. To our delight, the unsaturated double bond or triple bond in the substrates did not interfere with the reactivity or enantiocontrol of the reaction. For instance, reactions with substrate **1k** with a styryl group and substrate **1l** with a phenylethynyl group could proceed smoothly to give the corresponding products **2k** and **2l** in 88% yield, 94% ee and 91% yield, 93% ee, respectively. Furthermore, substrate **1m** with both a triple bond and a hydroxyl group was also well tolerated, and product **2m** was obtained in 80% yield with 78% ee. Various substituents at other positions of 2-hydroxy-1-naphthoate were also surveyed. Substrate **1n** with 4-Br, substrate **1o** with 7-Br and substrate **1p** with 7-MeO could all be successfully converted to their corresponding products in excellent yields and enantioselectivity (**2n–2p**, 85–95% yields, 90–94% ee). In addition, product **2q** with a 3-Br substituent was obtained in 88% yield with 73% ee. Apart from 2-hydroxy-1-naphthoates, 2-naphthols with an electron-donating group at the 1-position were also suitable substrates. For instance, substrate **1r** with 1,3-dimethyl groups could be smoothly transformed to the corresponding product **2r** (91% yield, 82% ee). To be noted, in the presence of (DHQ)_2_PHAL, substrates **1r** and **1s** with 1-Me and 3-Ph, respectively, could also work well in this reaction to yield **2r** (94% yield, 86% ee) and **2s** (89% yield, 82% ee) respectively. The absolute configuration of the product was determined, by X-ray analysis of enantiopure **2d**, as *R* (see the ESI† for details).

**Scheme 2:**
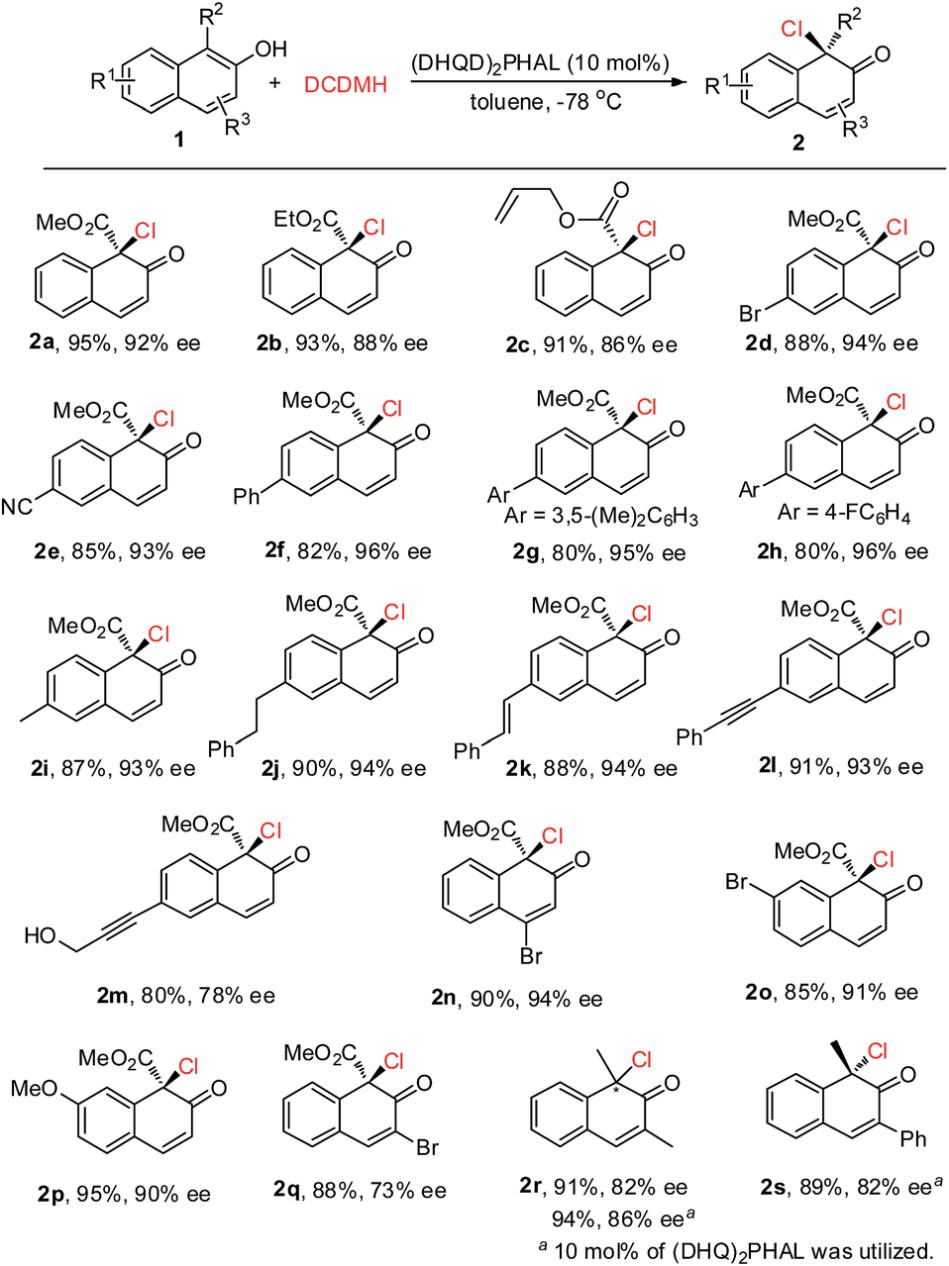
Evaluation of substrate scope.

Besides 2-naphthols, methyl 1-hydroxy-2-naphthoate, **1t**, was also well tolerated in this dearomative chlorination reaction. Under slightly optimized conditions (in the presence of 10 mol% of (DHQD)_2_PYR in CHCl_3_/CCl_4_ at –70 °C), product **2t** was obtained in 94% yield with 90% ee (Scheme 3), and its structure was confirmed by X-ray analysis. To our knowledge, highly enantioselective intermolecular dearomatization of 1-naphthol derivatives has not been reported yet.4*b*

**Scheme 3:**
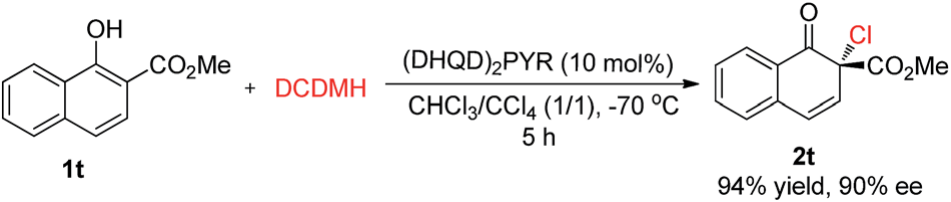
Asymmetric chlorinative dearomatization reaction of a 1-naphthol derivative.

We also tested the asymmetric bromination of **1a** with 1,3-dibromo-5,5-dimethylhydantoin (DBDMH) under the standard reaction conditions (eqn (1), Scheme 4). The desired brominative product **2u** was obtained in 96% yield with 9% ee. The almost racemic result was possibly due to the very strong background reaction. To our surprise, a further attempt using 2-hydroxy-1-naphthoic acid (**1v**) as the substrate in the presence of (DHQD)_2_PHAL provided the achiral decarboxylative compound **2v** in quantitative yield (eqn (2), Scheme 4).

**Scheme 4:**
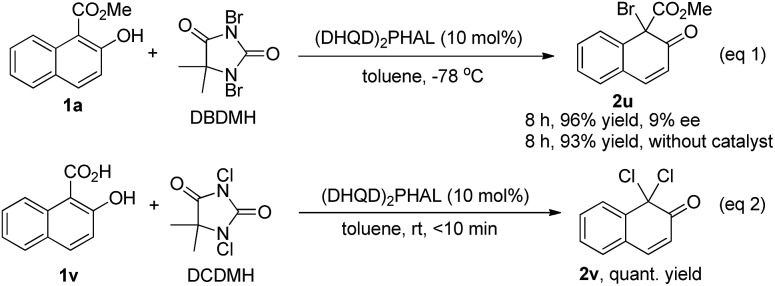
Bromination of **1a** with DBDMH (eqn 1) and reaction of 2-hydroxy-1-naphthoic acid (**1v**) with DCDMH (eqn 2).

To evaluate the practicality of this dearomative strategy, gram-scale reactions of **1a** and **1t** were performed. As displayed in Scheme 5, the corresponding products **2a** and **2t** could be obtained in excellent yields without a notable reduction in the enantioselectivity (91% ee and 87% ee, respectively).

**Scheme 5:**
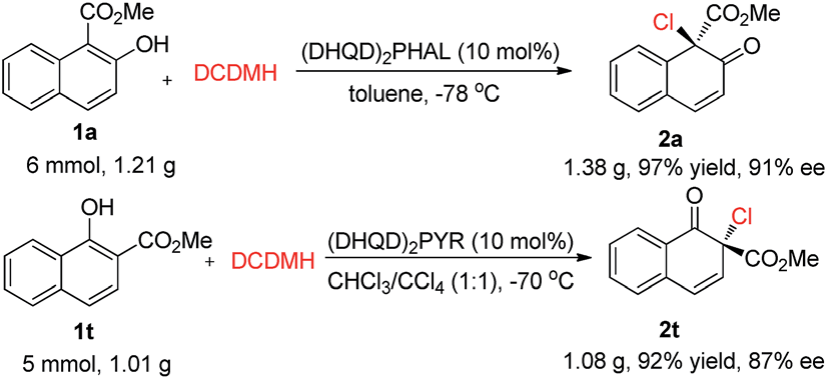
Gram-scale reactions.

To further show the synthetic utility of this newly developed protocol, several transformations of the products were carried out (Scheme 6). With different workup procedures, **2a** could be converted to the chiral allylic alcohol **3a** or epoxide **3b** in moderate yields with excellent diastereoselectivity (>20 : 1) *via* the reduction of carbonyl by Dibal-H (eqn (1) and (2), Scheme 6). When **2a** was subjected to oxidative bromination conditions, **2q** could be achieved in 74% yield with 89% ee, serving as a complementary route to access **2q** with high enantioselectivity (eqn (3), Scheme 6). In addition, **2t** could be converted to highly functionalized compounds through stereoselective halogenation of the double bond. For instance, the dibromination product **3c** could be obtained as a single diastereoisomer under bromination conditions, without reduction in the enantioselectivity (85% yield, 88% ee, eqn (4), Scheme 6). Furthermore, multi-functionalized chlorohydrin **3d** was obtained under electrophilic chlorination reaction conditions in 70% yield with good stereochemical integrity and high diastereoselectivity (dr = 11 : 1, 86% ee for the major diastereoisomer, eqn (5), Scheme 6).^17^

**Scheme 6:**
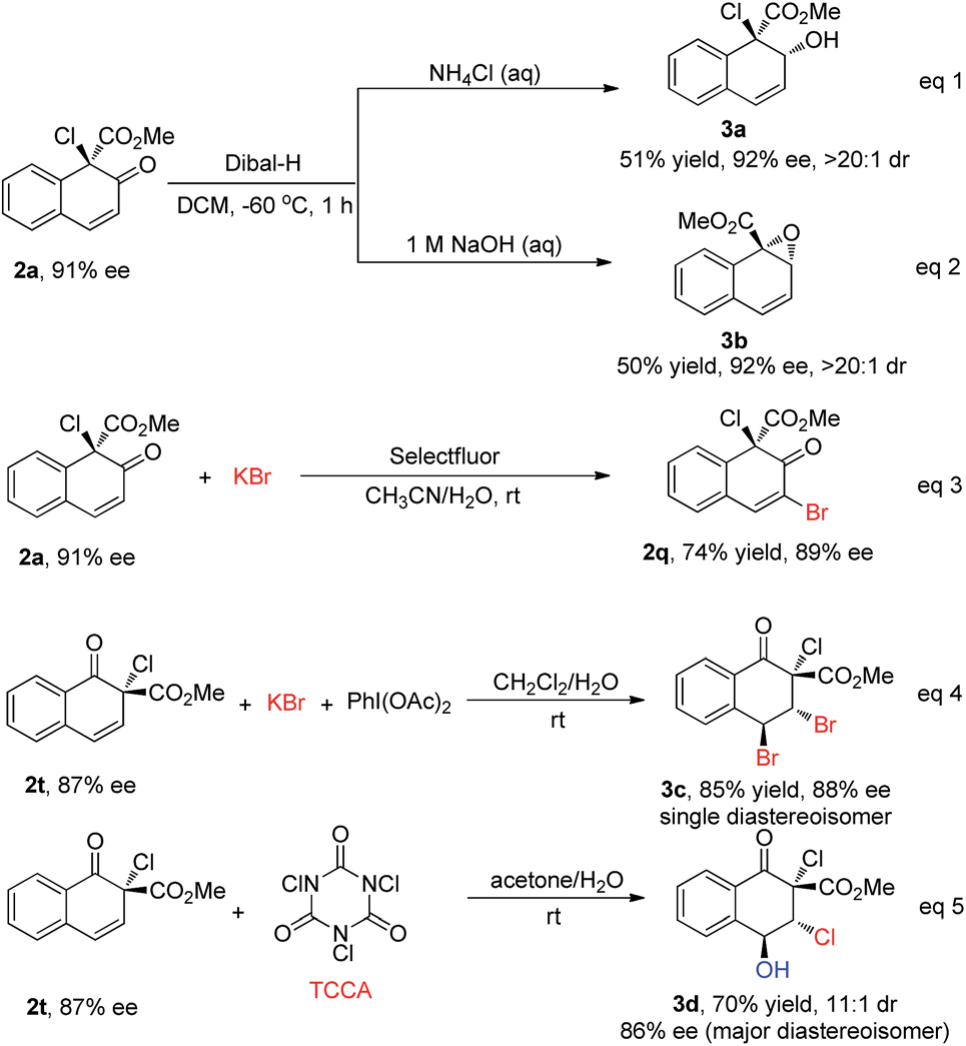
Transformations of products.

As for the working model of this reaction, inspired by the pioneering studies by Nicolaou,13*c* Hennecke13*k* and Tang,13*l* we speculate that the phthalazine nitrogen in the catalyst interacts with the hydroxyl group of naphthol *via* a hydrogen bond to increase the nucleophilic property of the 1-position (Fig. 1). In addition, the intramolecular hydrogen bond of **1a** itself also makes a contribution to a relatively rigid chiral environment. On the other hand, the tertiary amine nitrogen in quinuclidine acts as a Lewis base to activate the chloronium species to provide a bifunctional catalytic model, which is in line with Borhan's research.13*a*

**Fig. 1:**
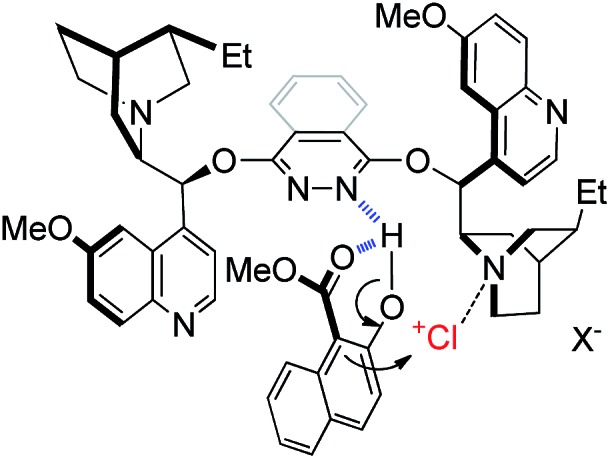
Proposed working model.

To investigate this proposal, several control experiments were carried out, as shown in Scheme 7. Firstly, when substrate **1u**, of which the hydroxyl was protected by a methyl group, was subjected to the chlorination conditions, no reaction occurred (eqn (1), Scheme 7). When the protecting group was changed to TMS, the reaction of **1v** proceeded very slowly to give the desired product **2a** in only 45% yield with 20% ee (eqn (2), Scheme 7). When a homogeneous toluene solution of the potassium salt of **1a**, prepared *in situ* by treating **1a** with 1.05 equiv of KOMe and 18-crown-6, was subjected to the standard conditions, product **2a** was obtained in 99% yield, however in an almost racemic form (eqn (3), Scheme 7). The control experiment (eqn (4), Scheme 7) revealed that the addition of methanol and 18-crown-6 did not have any effect on the yield or enantioselectivity of **2a**. All these experiments suggested that the hydroxyl group in the substrate is relevant not only to the reactivity but also to the enantiocontrol, possibly playing a role as a hydrogen bond donor. Furthermore, the addition of benzoic acid dramatically decreased the reaction rate as well as the enantioselectivity of **2a** from 92% ee to 64% ee (eqn (5), Scheme 7). The possible protonation of the quinuclidine nitrogen atom by the acid decreased the catalytic efficiency of the catalyst. Despite the fact that some promising experimental evidence was obtained, the working model is postulated and needs further studies.

**Scheme 7:**
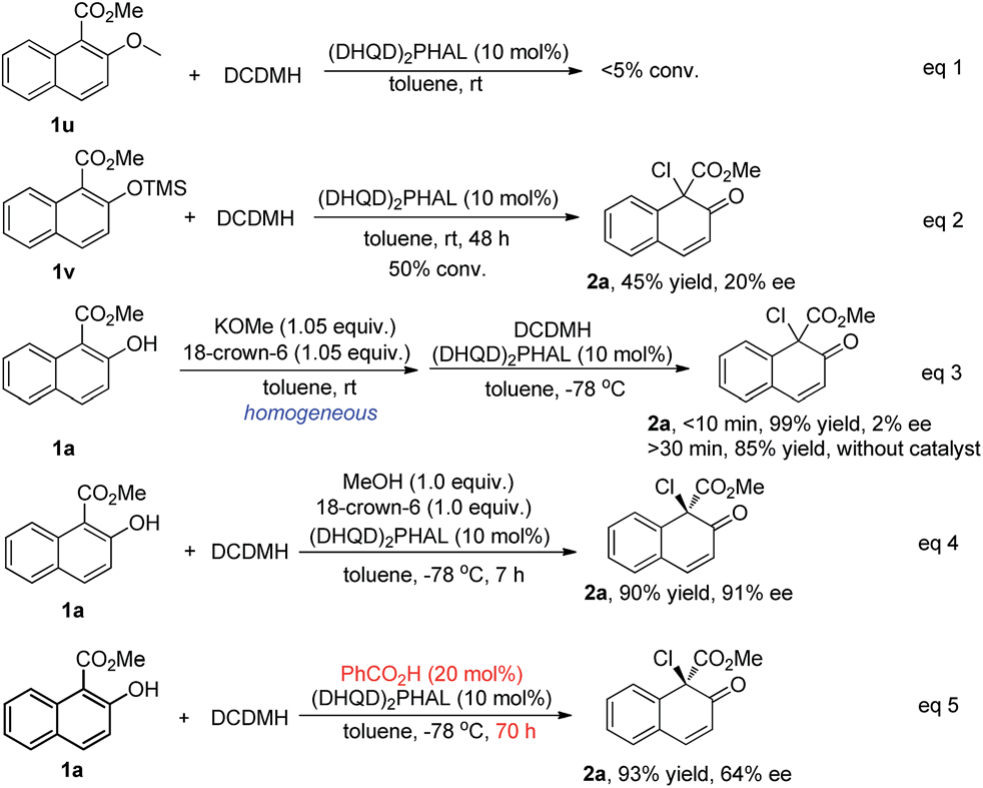
Control experiments.

In summary, we have realized for the first time the organocatalytic asymmetric chlorinative dearomatization of naphthols, providing chiral naphthalenones with a Cl-containing all-substituted stereocenter in excellent yields and enantioselectivity. The reaction features mild reaction conditions, good tolerance of diverse functional groups and simple reaction operation. Notably, highly enantioselective intermolecular dearomative chlorination of 1-naphthol derivative was also realized. In addition, the gram-scale reactions and practical transformations of the products reveal the potential synthetic utility of this method.

## Supplementary Material

Supplementary information

Crystal structure data
