# Supplementary material for: Organocatalytic asymmetric chlorinative dearomatization of naphthols
Source: Chem Sci. 2015 Apr 27;6(7):4179–83. doi: 10.1039/c5sc00494b (PMC5707459; doi:10.1039/c5sc00494b)
Supplement: Supplementary file 1 [file SC-006-C5SC00494B-s001.pdf]

Electronic Supplementary Material (ESI) for Chemical Science.
This journal is © The Royal Society of Chemistry 2015

Supporting Information for

Organocatalytic Asymmetric Chlorinative Dearomatization of Naphthols

*Qin Yin, Shou-Guo Wang, Xiao-Wei Liang, De-Wei Gao, Jun Zheng and Shu-Li You**

State Key Laboratory of Organometallic Chemistry
Shanghai Institute of Organic Chemistry, Chinese Academy of Sciences
345 Lingling Lu, Shanghai 200032, China
Fax: (+86) 21-54925087
E-mail: slyou@.sioc.ac.cn

Table of Contents

General considerations..S2

Procedures for the preparation of **1c, 1f-1q**..S2

General procedure for the asymmetric chlorination S9

Transformations of **2a** and **2t**……………………………………………...............S19

Mechanistic Investigations…………………………………………….............S23

Crystal data S25

References...S28

Copies of spectra...S29

1. General considerations

Unless stated otherwise, all solvents were purified and dried according to standard methods prior to use. ^{1}H and ^{19}F NMR spectra were recorded on Varian or Angilent instrument (400 MHz and 376 MHz, 300 MHz and 282 MHz, respectively) and referenced relative to tetramethylsilane signal or residual protio solvent signals and $CFCl_3$ respectively. ^{13}C NMR spectra were recorded on Varian or Angilent instrument (100 MHz or 75 MHz) and referenced relative to residual solvent signals. Data for ^{1}H NMR are recorded as follows: chemical shift (δ, ppm), multiplicity (s = singlet, d = doublet, t = triplet, m = multiplet or unresolved, br = broad singlet, coupling constant(s) in Hz, integration). Data for ^{13}C NMR and ^{19}F NMR are reported in terms of chemical shift (δ, ppm).

Methyl 2-hydroxy-1-naphthoate (**1a**) and Methyl 1-hydroxy-2-naphthoate (**1t**) were purchased from Alfa Aesar and used without further purification. Substituted 2-hydroxy-1-naphthoates (**1b**, **1d**, **1e**) and compounds (**1r**, **1s**) are known compounds and prepared according to the literature.[1]

2. Experimental procedures, analytical and spectroscopic data

2.1 Procedure for preparation of 1c

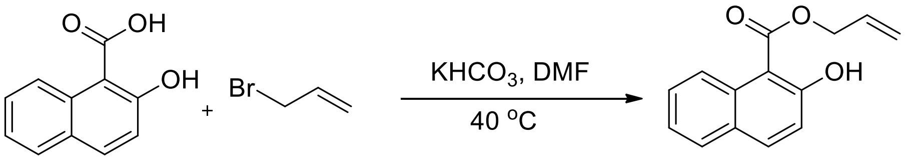

To a solution of 2-hydroxy-1-naphthoic acid (944 mg, 5 mmol) in DMF (10 mL), potassium hydrogen carbonate (600 mg, 6 mmol) was added. The mixture was stirred at rt for 10 min, followed by addition of allyl bromide (908 mg, 7.5 mmol). Then the mixture was stirred at 40 °C until the reaction was complete (monitored by TLC). The reaction was quenched by the addition of water (5 mL). The aqueous layer was extracted with ethyl acetate. The organic layer was washed with brine, dried over Na_2SO_4, filtered and then concentrated. The residue was purified by silica gel column chromatography (ethyl acetate/petroleum ether = 1/20, v/v) to afford the product.

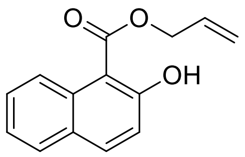

Allyl 2-hydroxy-1-naphthoate (1c)

Colorless liquid. Analytical data for **1c**: ^{1}H NMR (300 MHz, $CDCl_3$) δ 4.86 (d, *J* = 5.7 Hz, 2H), 5.24 (dd, *J* = 1.2, 10.5 Hz, 1H), 5.36 (dd, *J* = 1.2, 17.1 Hz, 1H), 5.94-6.05 (m, 1H), 7.02 (d, *J* = 9.0 Hz, 1H), 7.22 (t, *J* = 6.9 Hz, 1H), 7.42 (td, *J* = 8.4, 1.2 Hz, 1H), 7.59 (d, *J* = 8.1 Hz, 1H), 7.72 (d, *J* = 8.7 Hz, 1H), 8.65 (d, *J* = 9.0 Hz, 1H), 12.16 (s, 1H); ^{13}C NMR (75 MHz, $CDCl_3$) δ 66.3, 104.5, 119.2, 119.3, 123.5, 125.2, 128.4, 128.5, 129.0, 131.5, 131.7, 136.8, 164.4, 172.0; IR (film) 2986, 1641, 1201, 825 cm^{-1}; HRMS (ESI) exact mass calcd for $C_{14}H_{13}O_3^+$ (M+H) requires *m/z* 229.0859. Found *m/z* 229.0865.

2.2 General procedure for preparation of 1f-1h, 1k

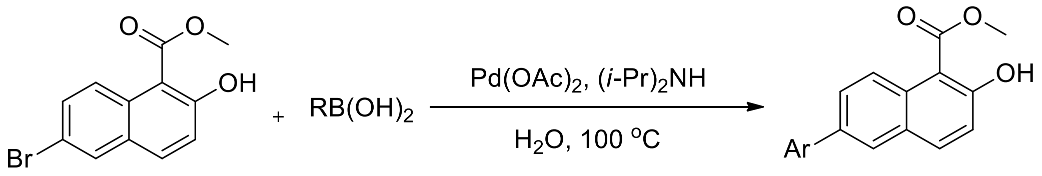

To a 25 mL two-neck round-bottomed flask equipped with a condenser, methyl 6-bromo-2-hydroxy-1-naphthoate (562 mg, 2.0 mmol), boronic acid (3.0 mmol), (*i*-Pr)$_2$NH (202 mg, 2.0 mmol), $Pd(OAc)_2$ (9.0 mg, 0.04 mmol) and H_2O (4.0 mL) were added successively. The mixture was reacted at 100 ^{o}C until the reaction was complete (monitored by TLC). The mixture was filtered through a pad of celite and washed with ethyl acetate. The aqueous layer was extracted with ethyl acetate. The organic layer was washed with brine, dried over Na_2SO_4, filtered and then concentrated. The residue was purified by silica gel column chromatography (ethyl acetate/petroleum ether = 1/50, v/v) to afford the product.

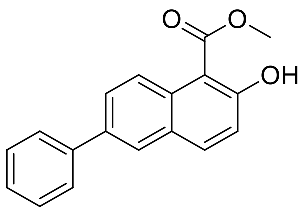

Methyl 2-hydroxy-6-phenyl-1-naphthoate (1f)

White solid. Analytical data for **1f**: ^{1}H NMR (400 MHz, CDCl$_3$) δ 4.04 (s, 3H), 7.12 (d, *J* = 8.8 Hz, 1H), 7.34-7.46 (m, 3H), 7.64-7.85 (m, 5H), 8.72 (d, *J* = 8.8 Hz, 1H), 12.31 (s, 1H); ^{13}C NMR (100 MHz, CDCl$_3$) δ 52.3, 104.4, 119.5, 125.8, 126.6, 126.9, 127.0, 127.3, 127.6, 128.8, 130.7, 136.0, 137.0, 140.1, 164.3, 172.7; IR (film) 2958, 1648, 1220 cm^{-1}; HRMS (ESI) exact mass calcd for $C_{18}H_{15}O_3^+$ (M+H) requires *m/z* 279.1016. Found *m/z* 279.1008.

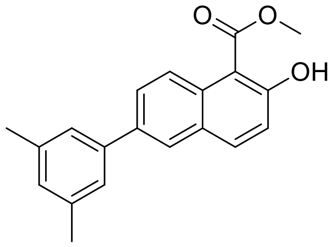

Methyl 6-(3,5-dimethylphenyl)-2-hydroxy-1-naphthoate (1g)

White solid. Analytical data for **1g**: ^{1}H NMR (400 MHz, CDCl$_3$) δ 2.41 (s, 6H), 4.11 (s, 3H), 7.02 (br, 1H), 7.17-7.32 (m, 3H), 7.79-7.92 (m, 3H), 8.77 (d, *J* = 7.2 Hz, 1H), 12.30 (s, 1H); ^{13}C NMR (100 MHz, CDCl$_3$) δ 21.5, 52.5, 104.6, 119.6, 125.0, 125.7, 126.8, 127.9, 129.0, 130.8, 135.7, 136.4, 137.1, 138.4, 140.3, 164.3, 172.8; IR (film) 2955, 1646, 1217 cm^{-1}; HRMS (ESI) exact mass calcd for $C_{20}H_{19}O_3^+$ (M+H) requires *m/z* 307.1329. Found *m/z* 307.1320.

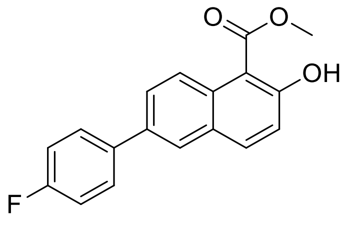

Methyl 6-(4-fluorophenyl)-2-hydroxy-1-naphthoate (1h)

White solid. Analytical data for **1h**: ^{1}H NMR (300 MHz, CDCl$_3$) δ 4.00 (s, 3H), 7.03-7.09 (m, 3H), 7.52 (br, 2H), 7.63 (d, *J* = 8.7 Hz, 1H), 7.74-7.80 (m, 2H), 8.66 (d, *J* = 8.7 Hz, 1H), 12.26 (s, 1H); ^{13}C NMR (75 MHz, CDCl$_3$) δ 52.8, 104.8, 116.0 (d, *J* = 21.2 Hz), 120.1, 126.2, 126.8, 127.8, 128.8 (d, *J* = 8.0 Hz), 129.1, 131.0, 135.4, 136.6, 137.3, 162.7 (d, *J* = 244.7 Hz), 164.7, 173.0; ^{19}F NMR (282 MHz, CDCl$_3$) δ -114.2; IR (film) 2957, 1647, 1216 cm^{-1}; HRMS (ESI) exact mass calcd for $C_{18}H_{14}FO_3^+$ (M+H) requires *m/z* 297.0921. Found *m/z* 297.0918.

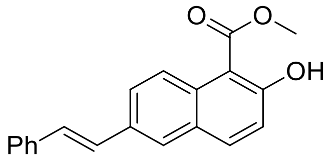

(*E*)-Methyl 2-hydroxy-6-styryl-1-naphthoate (1k)

Yellow solid. Analytical data for **1k**: ^{1}H NMR (400 MHz, $CDCl_3$) δ 4.09 (s, 3H), 7.15-7.19 (m, 3H), 7.31-7.42 (m, 3H), 7.55-7.57 (m, 2H), 7.71 (s, 1H), 7.76 (d, J = 8.4 Hz, 1H), 7.84 (d, J = 8.4 Hz, 1H), 8.69 (d, J = 8.8 Hz, 1H), 12.32 (s, 1H); ^{13}C NMR (100 MHz, $CDCl_3$) δ 52.5, 104.9, 119.7, 125.7, 126.2, 126.5, 127.4, 127.7, 127.9, 128.7, 128.8, 128.9, 131.2, 132.6, 136.9, 137.3, 164.4, 172.7; IR (film) 1645, 1335, 1225 cm^{-1}; HRMS (ESI) exact mass calcd for $C_{20}H_{17}O_3^+$ (M+H) requires *m/z* 305.1172. Found *m/z* 305.1167.

2.3 General procedure for preparation of 1i, 1n, 1o-1q (1q as an example, substituted 2-naphthols are commercially available)

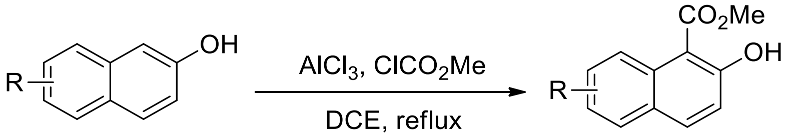

To a 25 mL two-neck round-bottomed flask equipped with a condenser, $AlCl_3$ (1.29 g, 10 mmol) and DCE (20 mL) were added successively. Then methyl chloroformate (945 mg, 10 mmol) was added and the mixture was stirred for 10 min at rt. 3-Bromo-2-naphthol (1.12 g, 5 mmol) was added and the mixture was stirred under reflux for 10 h. Then H_2O (10 mL) was added at 0 °C. The aqueous layer was extracted with DCM. The organic layer was washed with brine, dried over Na_2SO_4, filtered and then concentrated. The residue was purified by silica gel column chromatography (ethyl acetate/petroleum ether = 1/50, v/v) to afford the product.

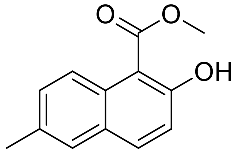

Methyl 2-hydroxy-6-methyl-1-naphthoate (1i)

Yellow solid. Analytical data for **1i**: ^{1}H NMR (400 MHz, $CDCl_3$) δ 2.37 (s, 3H), 3.99

(s, 3H), 7.04 (d, J = 8.8 Hz, 1H), 7.29 (d, J = 8.8 Hz, 1H), 7.42 (s, 1H), 7.71 (d, J = 8.8 Hz, 1H), 8.52 (d, J = 8.8 Hz, 1H), 12.10 (s, 1H); ^{13}C NMR (100 MHz, $CDCl_3$) δ 20.9, 52.3, 104.6, 119.2, 125.2, 128.3, 128.9, 129.7, 130.5, 133.1, 136.4, 163.8, 172.9; IR (film) 2956, 1639, 1215 cm^{-1}; HRMS (DART) exact mass calcd for $C_{13}H_{13}O_3^+$ (M+H) requires *m/z* 217.0859. Found *m/z* 217.0859.

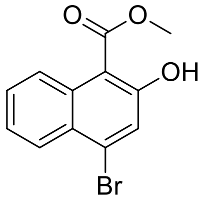

Methyl 4-bromo-2-hydroxy-1-naphthoate (1n)

Yellow solid. Analytical data for **1n**: ^{1}H NMR (400 MHz, $CDCl_3$) δ 3.99 (s, 3H), 7.33-7.35 (m, 1H), 7.42 (s, 1H), 7.42-7.46 (m, 1H), 8.09 (d, J = 8.4 Hz, 1H), 8.61 (d, J = 8.8 Hz, 1H), 12.10 (s, 1H); ^{13}C NMR (100 MHz, $CDCl_3$) δ 52.7, 104.7, 123.6, 124.8, 125.5, 127.1, 128.2, 129.1, 132.0, 132.3, 163.1, 172.3; IR (film) 1647, 1216 cm^{-1}; HRMS (DART) exact mass calcd for $C_{12}H_{10}O_3Br^+$ (M+H) requires *m/z* 280.9808. Found *m/z* 280.9806.

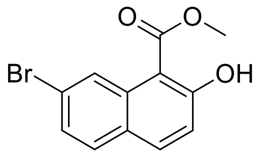

Methyl 7-bromo-2-hydroxy-1-naphthoate (1o)

Yellow solid. Analytical data for **1o**: ^{1}H NMR (400 MHz, $CDCl_3$) δ 4.03 (s, 3H), 7.07 (d, J = 9.2 Hz, 1H), 7.37 (dd, J = 8.8, 1.6 Hz, 1H), 7.50 (d, J = 8.4 Hz, 1H), 7.74 (d, J = 8.8 Hz, 1H), 8.82 (s, 1H), 12.30 (s, 1H); ^{13}C NMR (100 MHz, $CDCl_3$) δ 52.7, 103.9, 119.8, 123.5, 127.0, 127.8, 130.3, 130.4, 132.8, 136.6, 165.0, 172.4; IR (film) 2955, 1648, 1217 cm^{-1}; HRMS (DART) exact mass calcd for $C_{12}H_9O_3Br^+$ requires *m/z* 279.9730. Found *m/z* 279.9728.

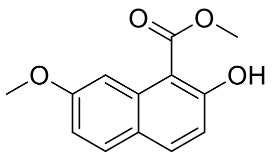

Methyl 2-hydroxy-7-methoxy-1-naphthoate (1p)

White solid. Analytical data for **1p**: ^{1}H NMR (400 MHz, $CDCl_3$) δ 3.92 (s, 3H), 4.08

(s, 3H), 6.98-7.02 (m, 2H), 7.62 (d, J = 8.8 Hz, 1H), 7.78 (d, J = 8.8 Hz, 1H), 8.16 (s, 1H), 12.30 (s, 1H); ^{13}C NMR (100 MHz, $CDCl_3$) δ 52.3, 55.2, 103.9, 106.2, 114.5, 116.6, 123.8, 130.5, 133.4, 136.7, 159.9, 165.1, 172.8; IR (film) 1643, 1617, 1194 cm^{-1}; HRMS (ESI) exact mass calcd for $C_{13}H_{13}O_4^+$ requires m/z 233.0808. Found m/z 233.0810.

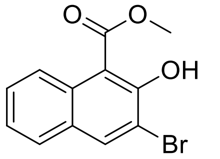

Methyl 3-bromo-2-hydroxy-1-naphthoate (1q)

Yellow solid. Analytical data for 1**q**: ^{1}H NMR (400 MHz, $CDCl_3$) δ 3.99 (s, 3H), 7.24 (td, J = 8.0, 2.7 Hz, 1H), 7.42 (td, J = 8.4, 1.2 Hz, 1H), 7.50 (d, J = 8.0 Hz, 1H), 8.03 (s, 1H), 8.51 (d, J = 8.8 Hz, 1H); ^{13}C NMR (100 MHz, $CDCl_3$) δ 52.9, 105.8, 112.9, 124.4, 125.3, 128.2, 128.7, 130.7, 139.2, 160.3, 172.6; IR (film) 2956, 1646, 1215 cm^{-1}; HRMS (ESI) exact mass calcd for $C_{12}H_{10}BrO_3^+$ (M+H) requires m/z 280.9808. Found m/z 280.9813.

2.4 Procedure for preparation of 1j

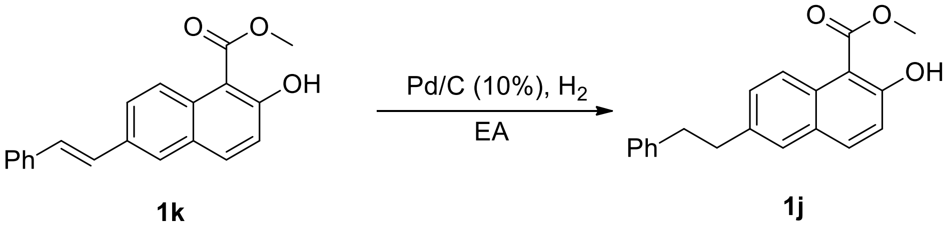

To a solution of **1k** (304 mg, 1 mmol) in ethyl acetate (3 mL), 10% Pd/C (20 mg) was added under Ar atmosphere. Then the reaction was charged with 1 atm of hydrogen and stirred at room temperature for 17 h. The reaction mixture was filtered through a pad of celite and washed with ethyl acetate. The filtrate was concentrated under reduced pressure. The crude product was purified by silica gel column chromatography (ethyl acetate/petroleum ether = 1/30, v/v) to afford the product.

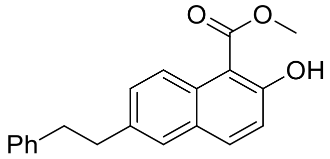

Methyl 2-hydroxy-6-phenethyl-1-naphthoate (1j)

White solid. Analytical data for **1j**: ^{1}H NMR (400 MHz, $CDCl_3$) δ 2.89-2.92 (m, 4H), 3.97 (s, 3H), 7.02-7.20 (m, 6H), 7.29 (d, *J* = 9.2 Hz, 1H), 7.39 (s, 1H), 7.69 (d, *J* = 8.8 Hz, 1H), 8.54 (d, *J* = 9.2 Hz, 1H), 12.13 (s, 1H); ^{13}C NMR (100 MHz, $CDCl_3$) δ 37.2, 37.6, 52.3, 104.5, 119.2, 125.3, 125.9, 127.8, 128.3, 128.4, 128.8, 129.7, 130.0, 136.5, 136.8, 141.5, 163.9, 172.8; IR (film) 1641, 1335, 1236 cm^{-1}; HRMS (ESI) exact mass calcd for $C_{20}H_{19}O_3^+$ (M+H) requires *m/z* 307.1329. Found *m/z* 307.1329.

2.5 General procedure for preparation of 1l-1m (1l as an example)

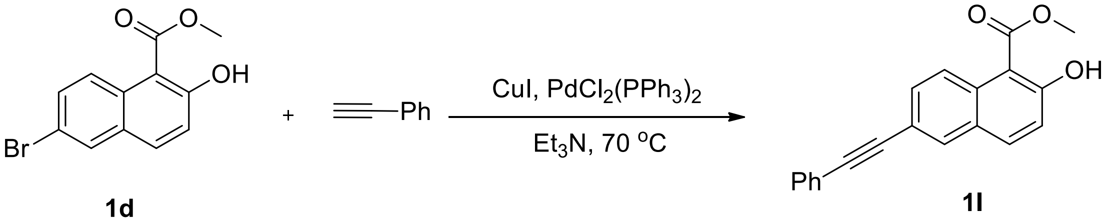

1d **1l**

To a solution of **1d** (843 mg, 3 mmol) in Et_3N (10 mL), phenylacetylene (460 mg, 4.5 mmol), bis(triphenylphosphine)palladium(II) chloride (84 mg, 0.12 mmol) and cuprous iodide (12 mg, 0.06 mmol) were added successively under Ar atmosphere. The reaction mixture was stirred at 70 °C until the reaction was complete (monitored by TLC). The mixture was filtered through a pad of celite, washed with ethyl acetate, and followed by concentration. The residue was purified by silica gel column chromatography (ethyl acetate/petroleum ether = 1/20, v/v) to afford the product.

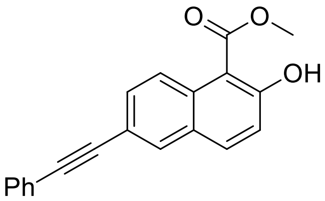

Methyl 2-hydroxy-6-(phenylethynyl)-1-naphthoate (1l)

Yellow solid. Analytical data for **1l**: ^{1}H NMR (400 MHz, $CDCl_3$) δ 4.07 (s, 3H), 7.14 (d, *J* = 8.8 Hz, 1H), 7.34-7.35 (m, 3H), 7 7.55-7.57 (m, 2H), 7.62 (dd, *J* = 9.2, 1.6 Hz, 1H), 7.80 (d, *J* = 8.8 Hz, 1H), 7.90 (d, *J* = 0.8 Hz, 1H), 8.66 (d, *J* = 8.8 Hz, 1H), 12.35 (s, 1H); ^{13}C NMR (100 MHz, $CDCl_3$) δ 52.6, 89.2, 89.7, 104.8, 118.4, 120.1, 123.2, 125.5, 128.3, 128.3, 128.4, 131.0, 131.3, 131.6, 132.3, 136.6, 164.9, 172.6; IR (film)

1657, 1323, 1211 cm^{-1}; HRMS (DART) exact mass calcd for $C_{20}H_{14}O_3^+$ requires *m/z* 302.0937. Found *m/z* 302.0936.

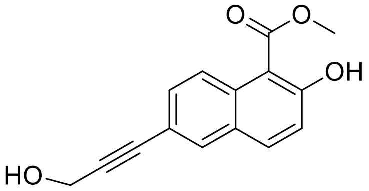

Methyl 2-hydroxy-6-(3-hydroxyprop-1-yn-1-yl)-1-naphthoate (1m)

Yellow solid. Analytical data for **1m**: ^{1}H NMR (400 MHz, $CDCl_3$) δ 2.34 (br, 1H), 3.98 (s, 3H), 4.47 (s, 2H), 7.03 (d, *J* = 9.2 Hz, 1H), 7.39 (dd, *J* = 9.2, 1.2 Hz, 1H), 7.63 (d, *J* = 9.2 Hz, 1H), 7.66 (s, 1H), 8.48 (d, *J* = 8.8 Hz, 1H), 12.26 (s, 1H); ^{13}C NMR (100 MHz, $CDCl_3$) δ 51.6, 52.6, 85.4, 87.5, 104.7, 117.6, 120.0, 125.4, 128.1, 130.9, 131.3, 132.4, 136.5, 164.8, 172.5; IR (film) 1639, 1230, 1028 cm^{-1}; HRMS (DART) exact mass calcd for $C_{15}H_{13}O_4^+$ (M+H) requires *m/z* 257.0808. Found *m/z* 257.0807.

2.6 General procedure for asymmetric chlorination of naphthols

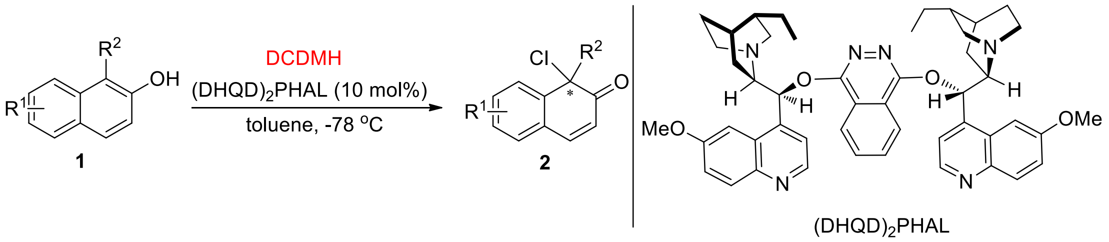

To a Schlenk tube, DCDMH (70.9 mg, 0.36 mmol), $(DHQD)_2PHAL$ (23.4 mg, 0.03 mmol) and toluene (2.0 mL) were added. After stirred for 10 min at -78 ^oC, **1** (0.3 mmol) was added in one portion. After the reaction was complete (monitored by TLC), the reaction was quenched by the addition of saturated Na_2SO_3 aqueous solution (3.0 mL). The organic layer was extracted with ethyl acetate, washed with brine, dried over Na_2SO_4, filtered and then concentrated. The residue was purified by silica gel column chromatography (ethyl acetate/petroleum ether = 1/20, v/v) to afford the product.

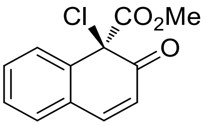

(*R*)-Methyl 1-chloro-2-oxo-1,2-dihydronaphthalene-1-carboxylate (2a)

Pale yellow solid (1.38 g, 97% yield, 6.0 mmol scale). Analytical data for **2a**: $[\alpha]_D^{20}$ = -58.5 (c = 1.0 $CHCl_3$, 92% ee); ^1H NMR (400 MHz, $CDCl_3$) δ 3.77 (s, 3H), 6.28 (d, *J* = 10.0 Hz, 1H), 7.37-7.54 (m, 5H); ^{13}C NMR (100 MHz, $CDCl_3$) δ 54.1, 67.3, 123.1, 128.3, 128.8, 129.9, 130.0, 131.0, 137.3, 145.7, 166.7, 189.8; IR (film) 1760, 1671, 1207, 1011 cm^{-1}; HRMS (ESI) exact mass calcd for $C_{12}H_{13}NO_3Cl^+$ (M+NH_4) requires *m/z* 254.0578. Found *m/z* 254.0577. The enantiomeric ratio was determined by Daicel Chiralpak OD-H (25 cm), Hexane / IPA = 90 / 10, 1.0 mL/min, λ = 254 nm, t (major) = 11.81 min, t (minor) = 14.26 min.

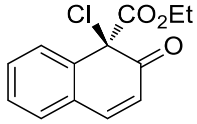

(*R*)-Ethyl 1-chloro-2-oxo-1,2-dihydronaphthalene-1-carboxylate (2b)

Pale yellow solid (92.8 mg, 93% yield, 0.4 mmol scale). Analytical data for **2b**: $[\alpha]_D^{20}$ = -43.4 (c = 1.0 $CHCl_3$, 88% ee); ^1H NMR (400 MHz, $CDCl_3$) δ 1.17 (t, *J* = 7.2 Hz, 3H), 4.18-4.29 (m, 2H), 6.27 (d, *J* = 10.4 Hz, 1H), 7.39-7.54 (m, 5H); ^{13}C NMR (100 MHz, $CDCl_3$) δ 13.7, 63.5, 67.3, 123.1, 128.2, 128.7, 129.9, 129.9, 130.9, 137.4, 145.6, 166.1, 189.9; IR (film) 1756, 1675, 1202, 1022 cm^{-1}; HRMS (ESI) exact mass calcd for $C_{13}H_{15}NO_3Cl^+$ (M+NH_4) requires *m/z* 268.0735. Found *m/z* 268.0729. The enantiomeric ratio was determined by Daicel Chiralpak OD-H (25 cm), Hexane / IPA = 90 / 10, 1.0 mL/min, λ = 254 nm, t (major) = 10.14 min, t (minor) = 11.84 min.

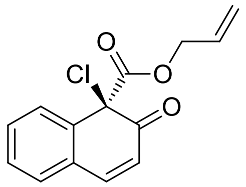

(*R*)-Allyl 1-chloro-2-oxo-1,2-dihydronaphthalene-1-carboxylate (2c)

Pale yellow solid (110.2 mg, 91% yield, 0.46 mmol scale). Analytical data for **2c**: $[\alpha]_D^{20}$ = -52.2 (c = 1.0 $CHCl_3$, 86% ee); ^1H NMR (400 MHz, $CDCl_3$) δ 4.65-4.67 (m, 2H), 5.14-5.18 (m, 2H), 5.73-5.80 (m, 1H), 6.28 (d, *J* = 10.0 Hz, 1H), 7.39-7.49 (m, 3H), 7.52-7.55 (m, 2H); ^{13}C NMR (100 MHz, $CDCl_3$) δ 67.3, 67.5, 118.9, 123.1, 128.2, 128.8, 129.9, 130.0, 130.4, 130.9, 137.2, 145.7, 165.8, 189.7; IR (film) 1757,

1672, 1197 cm^{-1}; HRMS (ESI) exact mass calcd for $C_{14}H_{15}NO_3Cl^+$ (M+NH_4) requires *m/z* 280.0735. Found *m/z* 280.0731. The enantiomeric ratio was determined by Daicel Chiralpak OD-H (25 cm), Hexane / IPA = 90 / 10, 0.6 mL/min, λ = 254 nm, t (major) = 17.96 min, t (minor) = 21.31 min.

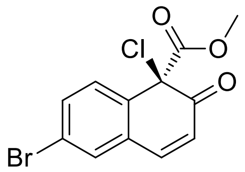

(*R*)-Methyl 6-bromo-1-chloro-2-oxo-1,2-dihydronaphthalene-1-carboxylate (2d)

Pale yellow solid (82.9 mg, 88% yield, 0.3 mmol scale). Analytical data for **2d**: $[\alpha]_D^{20}$ = -15.9 (c = 1.0 $CHCl_3$, 95% ee); ^{1}H NMR (400 MHz, $CDCl_3$) δ 3.77 (s, 3H), 6.32 (d, *J* = 10.4 Hz, 1H), 7.41 (d, *J* = 8.0 Hz, 1H), 7.44 (d, *J* = 10.0 Hz, 1H), 7.54 (d, *J* = 2.0 Hz, 1H), 7.59 (dd, *J* = 8.4, 2.0 Hz, 1H); ^{13}C NMR (100 MHz, $CDCl_3$) δ 54.4, 66.9, 124.1, 124.4, 130.1, 130.5, 132.5, 133.8, 136.1, 144.0, 166.3, 189.2; IR (film) 1759, 1675, 1202 cm^{-1}; HRMS (ESI) exact mass calcd for $C_{12}H_{12}NO_3ClBr^+$ (M+NH_4) requires *m/z* 331.9684. Found *m/z* 331.9676. The enantiomeric ratio was determined by Daicel Chiralpak IC (25 cm), Hexane / IPA = 90 / 10, 1.0 mL/min, λ = 254 nm, t (major) = 22.32 min, t (minor) = 24.32 min.

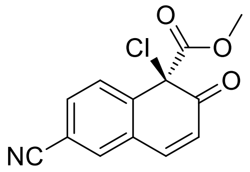

(*R*)-Methyl 1-chloro-6-cyano-2-oxo-1,2-dihydronaphthalene-1-carboxylate (2e)

Pale yellow solid (22.3 mg, 85% yield, 0.1 mmol scale). Analytical data for **2e**: $[\alpha]_D^{20}$ = 3.13 (c = 1.0 $CHCl_3$, 94% ee); ^{1}H NMR (400 MHz, $CDCl_3$) δ 3.77 (s, 3H), 6.40 (d, *J* = 10.0 Hz, 1H), 7.50 (d, *J* = 10.0 Hz, 1H), 7.65-7.75 (m, 3H); ^{13}C NMR (100 MHz, $CDCl_3$) δ 54.7, 66.7, 114.3, 117.2, 125.3, 129.5, 129.9, 132.9, 133.8, 141.6, 143.1, 165.8, 188.3; IR (film) 1733, 1675, 1251 cm^{-1}; HRMS (ESI) exact mass calcd for $C_{13}H_{12}N_2O_3Cl^+$ (M+NH_4) requires *m/z* 279.0531. Found *m/z* 279.0524. The enantiomeric ratio was determined by Daicel Chiralpak OD-H (25 cm), Hexane / IPA = 90 / 10, 1.0 mL/min, λ = 254 nm, t (major) = 37.72 min, t (minor) = 43.26 min.

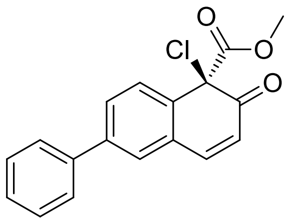

(*R*)-Methyl 1-chloro-2-oxo-6-phenyl-1,2-dihydronaphthalene-1-carboxylate (2f)

Pale yellow solid (76.8 mg, 82% yield, 0.3 mmol scale). Analytical data for **2f**: $[\alpha]_D^{20}$ = -32.3 (c = 1.0 $CHCl_3$, 96% ee); ^1H NMR (400 MHz, $CDCl_3$) δ 3.78 (s, 3H), 6.31 (d, *J* = 10.0 Hz, 1H), 7.39-7.49 (m, 3H), 7.55-7.60 (m, 5H), 7.65 (dd, *J* = 8.4, 2.0 Hz, 1H); ^{13}C NMR (100 MHz, $CDCl_3$) δ 54.3, 67.3, 123.6, 127.1, 128.4, 128.6, 128.8, 129.1, 129.4, 129.6, 135.9, 139.1, 143.2, 145.7, 166.8, 189.9; IR (film) 1754, 1677, 1016 cm^{-1}; HRMS (ESI) exact mass calcd for $C_{18}H_{17}NO_3Cl^+$ (M+NH_4) requires *m/z* 330.0891. Found *m/z* 330.0880. The enantiomeric ratio was determined by Daicel Chiralpak IC (25 cm), Hexane / IPA = 90 / 10, 1.0 mL/min, λ = 254 nm, t (major) = 36.20 min, t (minor) = 41.73 min.

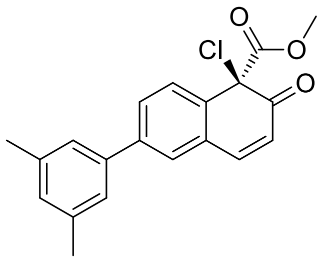

(*R*)-Methyl 1-chloro-6-(3,5-dimethylphenyl)-2-oxo-1,2-dihydronaphthalene-1-carboxylate (2g)

Pale yellow solid (81.6 mg, 80% yield, 0.3 mmol scale). Analytical data for **2g**: $[\alpha]_D^{20}$ = -25.5 (c = 1.0 $CHCl_3$, 95% ee); ^1H NMR (400 MHz, $CDCl_3$) δ 2.39 (s, 6H), 3.79 (s, 3H), 6.31 (d, *J* = 10.0 Hz, 1H), 7.06 (s, 1H), 7.20 (s, 2H), 7.55-7.58 (m, 3H), 7.64 (d, *J* = 8.4 Hz, 1H); ^{13}C NMR (100 MHz, $CDCl_3$) δ 21.4, 54.3, 67.3, 123.5, 125.0, 128.6, 128.7, 129.3, 129.6, 130.0, 135.7, 138.7, 139.1, 143.5, 145.7, 166.9, 189.9; IR (film) 1738, 1676, 1215 cm^{-1}; HRMS (ESI) exact mass calcd for $C_{20}H_{21}NO_3Cl^+$ (M+NH_4) requires *m/z* 358.1204. Found *m/z* 358.1203. The enantiomeric ratio was determined by Daicel Chiralpak IC (25 cm), Hexane / IPA = 90 / 10, 1.0 mL/min, λ = 254 nm, t

(major) = 31.31 min, t (minor) = 37.60 min.

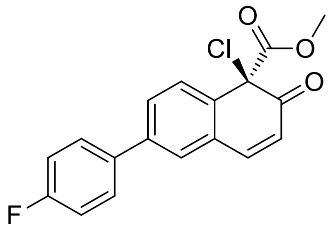

(*R*)-Methyl 1-chloro-6-(4-fluorophenyl)-2-oxo-1,2-dihydronaphthalene-1-carboxylate (2h)

Pale yellow foam (90.7 mg, 85% yield, 0.32 mmol scale). Analytical data for **2h**: $[\alpha]_D^{20}$ = -28.3 (c = 1.0 $CHCl_3$, 96% ee); ^1H NMR (300 MHz, $CDCl_3$) δ 3.76 (s, 3H), 6.30 (d, *J* = 10.0 Hz, 1H), 7.12-7.16 (m, 2H), 7.51-7.60 (m, 6H); ^{13}C NMR (100 MHz, $CDCl_3$) δ 54.2, 67.2, 115.9 (d, *J* = 21.3 Hz), 123.6, 128.3, 128.6, 128.7, 128.8, 129.3 (d, *J* = 13.7 Hz), 135.1 (d, *J* = 3.0 Hz), 135.8, 142.1, 145.5, 162.9 (d, *J* = 246.7 Hz), 166.7, 189.7; ^{19}F NMR (282 MHz, $CDCl_3$) δ -112.5; IR (film) 1757, 1676, 1224 cm^{-1}; HRMS (ESI) exact mass calcd for $C_{18}H_{16}ClFNO_3^+$ (M+NH_4) requires *m/z* 348.0797. Found *m/z* 348.0787. The enantiomeric ratio was determined by Daicel Chiralpak OD-H (25 cm), Hexane / IPA = 90 / 10, 0.6 mL/min, λ = 254 nm, t (major) = 31.73 min, t (minor) = 29.59 min.

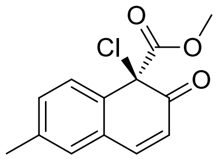

(*R*)-Methyl 1-chloro-6-methyl-2-oxo-1,2-dihydronaphthalene-1-carboxylate (2i)

Pale yellow foam (65.3 mg, 87% yield, 0.3 mmol scale). Analytical data for **2i**: $[\alpha]_D^{20}$ = -56.9 (c = 1.0 $CHCl_3$, 93% ee); ^1H NMR (400 MHz, $CDCl_3$) δ 2.39 (s, 3H), 3.76 (s, 3H), 6.25 (d, *J* = 10.0 Hz, 1H), 7.18 (s,1H), 7.27-7.25 (m, 1H), 7.41 (d, *J* = 8.0 Hz, 1H), 7.45 (d, *J* = 10.0 Hz, 1H); ^{13}C NMR (150 MHz, $CDCl_3$) δ 21.1, 54.1, 67.3, 123.2, 128.2, 128.9, 130.5, 131.7, 134.4, 140.3, 145.7, 166.9, 190.0; IR (film) 1756, 1674, 1232 cm^{-1}; HRMS (ESI) exact mass calcd for $C_{13}H_{12}ClO_3^+$ (M+H) requires *m/z* 251.0469. Found *m/z* 251.0471. The enantiomeric ratio was determined by Daicel Chiralpak IC (25 cm), Hexane / IPA = 90 / 10, 1.0 mL/min, λ = 254 nm, t (major) = 37.18 min, t (minor) = 45.75 min.

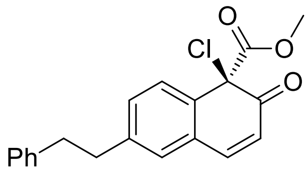

(*R*)-Methyl 1-chloro-2-oxo-6-phenethyl-1,2-dihydronaphthalene-1-carboxylate (2j)

Pale yellow solid (91.8 mg, 90% yield, 0.3 mmol scale). Analytical data for **2j**: $[\alpha]_D^{20}$ = -46.2 (c = 1.0 $CHCl_3$, 94% ee); ^{1}H NMR (400 MHz, $CDCl_3$) δ 2.93-2.96 (m, 4H), 3.75 (s, 3H), 6.25 (d, *J* = 9.6 Hz, 1H), 7.17-7.31 (m, 7H), 7.42-7.46 (m, 2H); ^{13}C NMR (100 MHz, $CDCl_3$) δ 37.2, 37.2, 54.1, 67.3, 123.1, 126.1, 128.2, 128.3, 128.4, 128.9, 130.0, 131.1, 134.8, 140.8, 144.0, 145.8, 166.8, 190.0; IR (film) 1762, 1675, 1213 cm^{-1}; HRMS (ESI) exact mass calcd for $C_{20}H_{21}ClNO_3^+$ (M+NH_4) requires *m/z* 358.1204. Found *m/z* 358.1206. The enantiomeric ratio was determined by Daicel Chiralpak IC (25 cm), Hexane / IPA = 90 / 10, 1.0 mL/min, λ = 254 nm, t (major) = 39.45 min, t (minor) = 47.75 min.

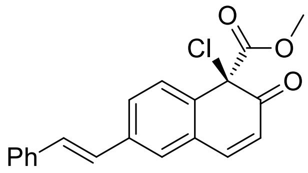

(*R*,*E*)-Methyl 1-chloro-2-oxo-6-styryl-1,2-dihydronaphthalene-1-carboxylate (2k)

Yellow solid (89.7 mg, 88% yield, 0.3 mmol scale). Analytical data for **2k**: $[\alpha]_D^{20}$ = -30.3 (c = 1.0 $CHCl_3$, 94% ee); ^{1}H NMR (400 MHz, $CDCl_3$) δ 3.77 (s, 3H), 6.30 (d, *J* = 9.6 Hz, 1H), 7.08 (d, *J* = 16.0 Hz, 1H), 7.18 (d, *J* = 16.0 Hz, 1H), 7.31-7.33 (m, 1H), 7.39 (t, *J* = 7.2 Hz, 2H), 7.48-7.57 (m, 6H); ^{13}C NMR (100 MHz, $CDCl_3$) δ 54.2, 67.3, 123.6, 126.4, 126.7, 127.7, 128.4, 128.6, 128.7, 128.8, 129.3, 131.2, 135.7, 136.4, 139.3, 145.5, 166.7, 189.8; IR (film) 1761, 1674, 1211 cm^{-1}; HRMS (ESI) exact mass calcd for $C_{20}H_{19}ClNO_3^+$ (M+NH_4) requires *m/z* 356.1048. Found *m/z* 356.1047. The enantiomeric ratio was determined by Daicel Chiralpak AD-H (25 cm), Hexane / IPA = 90 / 10, 1.0 mL/min, λ = 254 nm, t (major) = 24.14 min, t (minor) = 20.81 min.

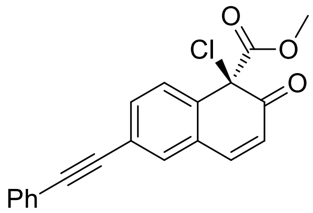

(*R*)-Methyl

1-chloro-2-oxo-6-(phenylethynyl)-1,2-dihydronaphthalene-1-carboxylate (2l)

Yellow solid (92.2 mg, 91% yield, 0.3 mmol scale). Analytical data for **2l**: $[\alpha]_D^{20}$ = -17.4 (c = 1.0 $CHCl_3$, 93% ee); ^1H NMR (400 MHz, $CDCl_3$) δ 3.77 (s, 3H), 6.31 (d, *J* = 10.0 Hz, 1H), 7.37-7.38 (m, 3H), 7.47-7.60 (m, 6H); ^{13}C NMR (100 MHz, $CDCl_3$) δ 54.3, 67.1, 87.5, 91.9, 122.3, 123.9, 125.4, 128.4, 128.5, 128.9, 128.9, 131.7, 132.6, 133.6, 136.5, 144.9, 166.4, 189.4; IR (film) 1769, 1680, 1221 cm^{-1}; HRMS (ESI) exact mass calcd for $C_{20}H_{17}ClNO_3^+$ (M+NH_4) requires *m/z* 354.0891. Found *m/z* 354.0892. The enantiomeric ratio was determined by Daicel Chiralpak IC (25 cm), Hexane / IPA = 90 / 10, 1.0 mL/min, λ = 254 nm, t (major) = 25.08 min, t (minor) = 27.97 min.

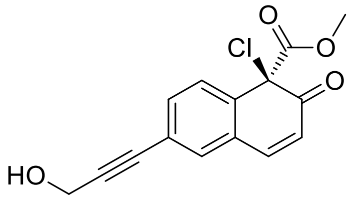

(*R*)-Methyl

1-chloro-6-(3-hydroxyprop-1-yn-1-yl)-2-oxo-1,2-dihydronaphthalene-1-carboxylate (2m)

Yellow foam (69.4 mg, 80% yield, 0.3 mmol scale). Analytical data for **2m**: $[\alpha]_D^{20}$ = -20.6 (c = 1.0 $CHCl_3$, 78% ee); ^1H NMR (400 MHz, $CDCl_3$) δ 2.18 (br, 1H), 3.77 (s, 3H), 4.52 (s, 2H), 6.30 (d, *J* = 10.4 Hz, 1H), 7.43-7.49 (m, 4H); ^{13}C NMR (100 MHz, $CDCl_3$) δ 51.4, 54.4, 67.1, 83.7, 89.9, 124.0, 124.8, 128.5, 129.0, 132.7, 133.7, 136.9, 144.8, 166.5, 189.5; IR (film) 1761, 1675, 1214 cm^{-1}; HRMS (ESI) exact mass calcd for $C_{15}H_{15}ClNO_4^+$ (M+NH_4) requires *m/z* 308.0684. Found *m/z* 308.0685. The enantiomeric ratio was determined by Daicel Chiralpak AD-H (25 cm), Hexane / IPA = 90 / 10, 1.0 mL/min, λ = 254 nm, t (major) = 32.70 min, t (minor) = 30.07 min.

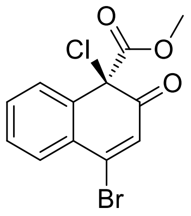

(*R*)-Methyl 4-bromo-1-chloro-2-oxo-1,2-dihydronaphthalene-1-carboxylate (2n)

Yellow foam (55.9 mg, 90% yield, 0.2 mmol scale). Analytical data for **2n**: $[\alpha]_D^{20}$ = -38.4 (c = 1.0 $CHCl_3$, 92% ee); ^{1}H NMR (400 MHz, $CDCl_3$) δ 3.77 (s, 3H), 6.85 (s, 1H), 7.53-7.54 (m, 3H), 7.96-7.98 (m, 1H); ^{13}C NMR (150 MHz, $CDCl_3$) δ 54.4, 67.5, 127.2, 127.7, 128.7, 130.3, 130.4, 132.0, 136.2, 145.6, 166.3, 186.7; IR (film) 1758, 1670, 1237 cm^{-1}; HRMS (ESI) exact mass calcd for $C_{12}H_{12}BrClNO_3^+$ (M+NH_4) requires *m/z* 331.9684. Found *m/z* 331.9686. The enantiomeric ratio was determined by Daicel Chiralpak OD-H (25 cm), Hexane / IPA = 90 / 10, 1.0 mL/min, λ = 254 nm, t (major) = 12.17 min, t (minor) = 19.71 min.

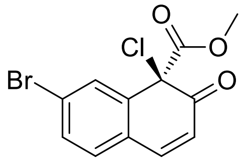

(*R*)-Methyl 7-bromo-1-chloro-2-oxo-1,2-dihydronaphthalene-1-carboxylate (2o)

Yellow foam (53.6 mg, 85% yield, 0.2 mmol scale). Analytical data for **2o**: $[\alpha]_D^{20}$ = -11.8 (c = 1.0 $CHCl_3$, 91% ee); ^{1}H NMR (400 MHz, $CDCl_3$) δ 3.78 (s, 3H), 6.29 (d, *J* = 10.0 Hz, 1H), 7.23 (d, *J* = 8.0 Hz, 1H), 7.44 (d, *J* = 10.0 Hz, 1H), 7.57 (dd, *J* = 8.4, 2.0 Hz, 1H), 7.67 (d, *J* = 2.0 Hz, 1H); ^{13}C NMR (100 MHz, $CDCl_3$) δ 54.5, 66.7, 123.6, 125.6, 127.3, 131.0, 132.1, 133.3, 139.1, 144.5, 166.3, 188.9; IR (film) 1763, 1673, 1218 cm^{-1}; HRMS (ESI) exact mass calcd for $C_{12}H_{12}BrClNO_3^+$ (M+NH_4) requires *m/z* 331.9684. Found *m/z* 331.9686. The enantiomeric ratio was determined by Daicel Chiralpak IC (25 cm), Hexane / IPA = 90 / 10, 1.0 mL/min, λ = 254 nm, t (major) = 20.79 min, t (minor) = 27.06 min.

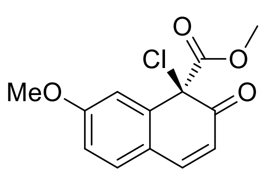

(*R*)-Methyl 1-chloro-7-methoxy-2-oxo-1,2-dihydronaphthalene-1-carboxylate

(2p)

Yellow solid (78.0 mg, 95% yield, 0.3 mmol scale). Analytical data for **2p**: $[\alpha]_D^{20}$ = -55.5 (c = 1.0 $CHCl_3$, 90% ee); ^{1}H NMR (400 MHz, $CDCl_3$) δ 3.76 (s, 3H), 3.85 (s, 3H), 6.13 (d, *J* = 10.0 Hz, 1H), 6.94 (dd, *J* = 8.4, 2.8 Hz, 1H), 7.05 (d, *J* = 2.4 Hz, 1H), 7.32 (d, *J* = 8.4 Hz, 1H), 7.47 (d, *J* = 10.0 Hz, 1H); ^{13}C NMR (100 MHz, $CDCl_3$) δ 54.2, 55.7, 67.2, 115.1, 115.1, 120.5, 121.4, 131.7, 139.4, 145.9, 161.9, 166.8, 189.9; IR (film) 1752, 1662, 1602, 1225 cm^{-1}; HRMS (ESI) exact mass calcd for $C_{13}H_{12}ClO_4^+$ (M+H) requires *m/z* 267.0419. Found *m/z* 267.0421. The enantiomeric ratio was determined by Daicel Chiralpak IC (25 cm), Hexane / IPA = 90 / 10, 1.0 mL/min, λ = 254 nm, t (major) = 41.17 min, t (minor) = 59.60 min.

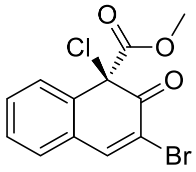

(*R*)-Methyl 3-bromo-1-chloro-2-oxo-1,2-dihydronaphthalene-1-carboxylate (2q)

Yellow foam (83.2 mg, 88% yield, 0.3 mmol scale). Analytical data for 2**q**: $[\alpha]_D^{20}$ = -37.0 (c = 1.0 $CHCl_3$, 73% ee); ^{1}H NMR (400 MHz, $CDCl_3$) δ 3.74 (s, 3H), 7.32 (d, *J* = 6.8 Hz, 1H), 7.42-7.49 (m, 3H), 7.91 (s, 1H); ^{13}C NMR (150 MHz, $CDCl_3$) δ 54.5, 68.0, 119.0, 128.7, 129.0, 129.5, 130.3, 131.3, 136.8, 146.9, 166.1, 183.6; IR (film) 1759, 1679, 1246, 1222 cm^{-1}; HRMS (ESI) exact mass calcd for $C_{12}H_{12}BrClNO_3^+$ (M+NH_4) requires *m/z* 331.9684. Found *m/z* 331.9673. The enantiomeric ratio was determined by Daicel Chiralpak IC (25 cm), Hexane / IPA = 90 / 10, 1.0 mL/min, λ = 254 nm, t (major) = 21.28 min, t (minor) = 27.04 min.

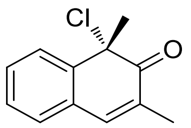

(*R*)-Methyl 1-chloro-3-methyl-2-oxo-1,2-dihydronaphthalene-1-carboxylate (2r)

Yellow liquid (19.3 mg, 94% yield, 0.1 mmol scale, 10 mol% of $(DHQ)_2$PHAL was utilized). Analytical data for **2r**: $[\alpha]_D^{20}$ = 52.4 (c = 1.0 $CHCl_3$, 86% ee); ^{1}H NMR (400 MHz, $CDCl_3$) δ 2.01 (s, 3H), 2.07 (s, 3H), 7.23-7.26 (m, 2H), 7.32-7.41 (m, 2H), 7.71 (d, *J* = 7.6 Hz, 1H); ^{13}C NMR (100 MHz, $CDCl_3$) δ 16.1, 28.2, 65.4, 127.7, 128.7,

128.8, 129.0, 129.5, 131.5, 140.9, 141.6, 194.7; IR (film) 1669, 1261, 756 cm^{-1}; HRMS (ESI) exact mass calcd for $C_{12}H_{12}OCl^+$ (M+H) requires *m/z* 207.0571. Found *m/z* 207.0575. The enantiomeric ratio was determined by Daicel Chiralpak IC (25 cm), Hexane / IPA = 49 / 1, 0.5 mL/min, λ = 254 nm, t (major) = 25.53 min, t (minor) = 23.29 min.

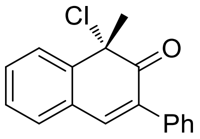

(*S*)-1-Chloro-1-methyl-3-phenylnaphthalen-2(1H)-one (2s)

Yellow liquid (23.8 mg, 89% yield, 0.1 mmol scale, 10 mol% of $(DHQ)_2PHAL$ was utilized). Analytical data for **2s**: $[\alpha]_D^{20}$ = 212.9 (c = 1.0 $CHCl_3$, 82% ee); ^1H NMR (400 MHz, $CDCl_3$) δ 2.13 (s, 3H), 7.37-7.47 (m, 7H), 7.54-7.56 (m, 2H), 7.77 (d, *J* = 7.2 Hz, 1H); ^{13}C NMR (100 MHz, $CDCl_3$) δ 26.6, 66.2, 127.5, 128.4, 128.4, 128.5, 129.2, 129.4, 129.9, 130.1, 134.7, 135.1, 140.3, 141.2, 193.0; IR (film) 1663, 1361, 761 cm^{-1}; HRMS (ESI) exact mass calcd for $C_{17}H_{14}OCl^+$ (M+H) requires *m/z* 269.0728. Found *m/z* 269.0721. The enantiomeric ratio was determined by Daicel Chiralpak AD-H (25 cm), Hexane / IPA = 60 / 1, 0.61 mL/min, λ = 254 nm, t (major) = 23.18 min, t (minor) = 21.43 min.

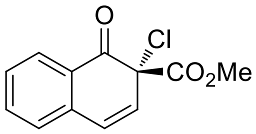

(*R*)-Methyl 2-chloro-1-oxo-1,2-dihydronaphthalene-2-carboxylate (2t)

The reaction was carried out at -70 °C in $CHCl_3/CCl_4$ (1.0 mL : 1.0 mL) with 10 mol% of $(DHQD)_2PYR$. Yellow solid (66.5 mg, 94% yield, 0.3 mmol scale). Analytical data for **2t**: $[\alpha]_D^{20}$ = -116.6 (c = 1.0 $CHCl_3$, 90% ee); ^1H NMR (400 MHz, $CDCl_3$) δ 3.79 (s, 3H), 6.22 (d, *J* = 9.6 Hz, 1H), 6.72 (d, *J* = 9.6 Hz, 1H), 7.29 (d, *J* = 7.6 Hz, 1H), 7.45 (t, *J* = 7.6 Hz, 1H), 7.65 (t, *J* = 7.6 Hz, 1H), 8.08 (d, *J* = 7.6 Hz, 1H); ^{13}C NMR (100 MHz, $CDCl_3$) δ 54.2, 65.4, 127.4, 128.2, 128.3, 128.4, 128.9, 129.6, 135.7, 136.1, 166.5, 189.2; IR (film) 1756, 1683, 1216 cm^{-1}; HRMS (ESI) exact mass calcd for $C_{12}H_{10}ClO_3^+$ (M+H) requires *m/z* 237.0313. Found *m/z* 237.0312. The enantiomeric

ratio was determined by Daicel Chiralpak OD-H (25 cm), Hexane / IPA = 90 / 10, 1.0 mL/min, λ = 254 nm, t (major) = 9.23 min, t (minor) = 10.27 min.

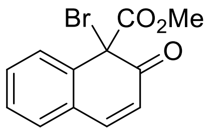

Methyl 1-bromo-2-oxo-1,2-dihydronaphthalene-1-carboxylate (2u)

The reaction was carried out with 1.2 equiv. of 1,3-dibromo-5,5-dimethylhydantoin. Pale yellow solid (80.9 mg, 96% yield, 9% ee, 0.3 mmol scale). **2u** is sensitive to proton solvent. Analytical data for **2u**: ^{1}H NMR (400 MHz, $CDCl_3$) δ 3.78 (s, 3H), 6.28 (d, J = 9.6 Hz, 1H), 7.36-7.49 (m, 5H); ^{13}C NMR (100 MHz, $CDCl_3$) δ 54.4, 60.2, 123.1, 127.9, 129.1, 130.0, 130.1, 137.9, 145.1, 166.6, 189.9; IR (film) 2951, 1750, 1671, 1237 cm^{-1}; HRMS (ESI) exact mass calcd for $C_{12}H_9BrO_3^+$ (M+NH_4) requires m/z 298.0073. Found m/z 298.0075. The enantiomeric ratio was determined by Daicel Chiralpak OD-H (25 cm), Hexane / IPA = 90 / 10, 1.0 mL/min, λ = 254 nm, t (major) = 14.07 min, t (minor) = 19.85 min.

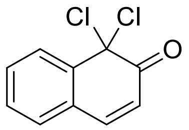

1,1-Dichloronaphthalen-2(1H)-one (2v)

The reaction was carried out at rt with 10 mol% of DMAP. White solid (63.5 mg, 100% yield, 0.3 mmol scale). Analytical data for **2v**: ^{1}H NMR (400 MHz, $CDCl_3$) δ 6.33 (d, J = 10.0 Hz, 1H), 7.32 (dd, J_1 = 7.6, J_2 = 1.2 Hz, 1H), 7.41-7.46 (m, 2H), 7.52 (m, td, J_1 = 7.6, J_2 = 1.2 Hz,, 1H), 8.06 (dd, J_1 = 8.0, J_2 = 0.8 Hz,, 1H). ^{13}C NMR (100 MHz, $CDCl_3$) δ 80.5, 122.6, 127.0, 129.6, 129.6, 130.7, 131.3, 140.7, 144.9, 185.9; IR (film) 3359, 3085, 1683, 1231 cm^{-1}; HRMS (EI) exact mass calcd for $C_{10}H_6Cl_2O^+$ requires m/z 211.9790. Found m/z 211.9800.

2.7 Transformations of 2a and 2t.

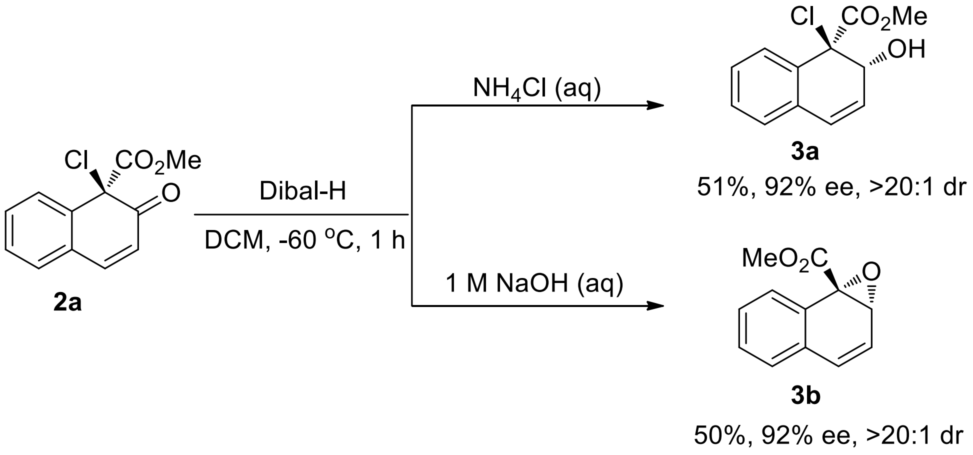

To a solution of **2a** (70.8 mg, 0.3 mmol) in DCM (2.0 mL), Dibal-H (0.4 mL, 1.5 M in toluene) was added dropwise at -60 °C under Ar atmosphere. After the reaction was complete (monitored by TLC), saturated NH_4Cl aqueous solution or 1 M NaOH aqueous solution (2.0 mL) was added. The aqueous layer was extracted with DCM. The organic layer was washed with brine, dried over Na_2SO_4, filtered and then concentrated. The residue was purified by silica gel column chromatography (ethyl acetate/petroleum ether/Et_3N = 2/40/1, v/v/v) to afford the product.

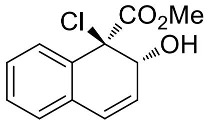

(1*R*,2*R*)-Methyl 1-chloro-2-hydroxy-1,2-dihydronaphthalene-1-carboxylate (3a)

Yellow solid (36.2 mg, 51% yield, 0.3 mmol scale). Analytical data for **3a**: $[\alpha]_D^{20}$ = -60.0 (c = 1.0 $CHCl_3$, 92% ee); ^{1}H NMR (400 MHz, $CDCl_3$) δ 3.53 (d, *J* = 9.6 Hz, 1H), 3.67 (s, 3H), 4.79 (d, *J* = 8.8 Hz, 1H), 6.03 (dd, *J* = 9.6, 2.8 Hz, 1H), 6.38 (dd, *J* = 9.6, 2.0 Hz, 1H), 7.02-7.04 (m, 1H), 7.22-7.28 (m, 2H), 7.11 (dd, *J* = 6.4, 3.2 Hz, 1H); ^{13}C NMR (100 MHz, $CDCl_3$) δ 53.6, 72.4, 74.8, 127.1, 127.9, 128.3, 128.8, 129.6, 130.6, 132.1, 132.3, 169.7; IR (film) 3444, 1721, 1267, 1208 cm^{-1}; HRMS (DART) exact mass calcd for $C_{12}H_{15}O_3NCl^+$ (M+NH_4) requires *m/z* 256.0735. Found *m/z* 256.0732. The enantiomeric ratio was determined by Daicel Chiralpak AD-H (25 cm), Hexane / IPA = 90 / 10, 1.0 mL/min, λ = 254 nm, t (major) = 9.25 min, t (minor) = 10.15 min.

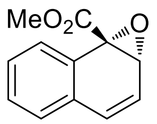

(1a*R*,7b*S*)-Methyl 1a,7b-dihydronaphtho[1,2-b]oxirene-7b-carboxylate (3b)

Yellow solid (30.3 mg, 50% yield, 0.3 mmol scale). Analytical data for **3b**: $[\alpha]_D^{20}$ = 124.9 (c = 1.0 $CHCl_3$, 92% ee); ^{1}H NMR (400 MHz, $CDCl_3$) δ 3.83 (s, 3H), 4.13-4.14 (m, 1H), 6.30 (dd, *J* = 9.6, 3.6 Hz, 1H), 6.76 (dd, *J* = 9.6, 1.2 Hz, 1H), 7.25-7.35 (m, 3H), 7.68 (dd, *J* = 7.2, 1.2 Hz, 1H); ^{13}C NMR (100 MHz, $CDCl_3$) δ 52.8, 58.9, 59.9, 122.5, 128.2, 129.0, 129.2, 129.2, 129.8, 131.8, 133.2, 168.6; IR (film) 1727, 1211, 1020 cm^{-1}; HRMS (DART) exact mass calcd for $C_{12}H_{11}O_3^+$ (M+H) requires *m/z* 203.0703. Found *m/z* 203.0701. The enantiomeric ratio was determined by Daicel Chiralpak AD-H (25 cm), Hexane / IPA = 90 / 10, 1.0 mL/min, λ = 254 nm, t (major) = 13.07 min, t (minor) = 11.61 min.

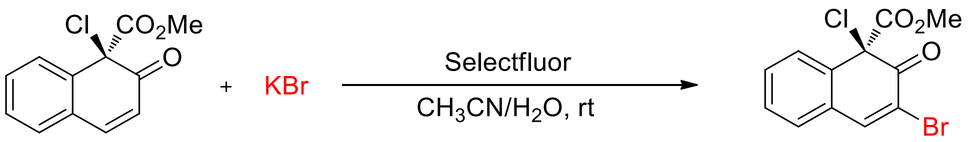

2a, 91% ee　　**2q**, 74%, 89% ee

To a stirred mixture of **2a** (23.6 mg, 0.1 mmol) and KBr (30.0 mg, 0.25 mmol) in CH_3CN (1.0 mL) and water (40 μL), Selectfluor (70.8 mg, 0.2 mmol) was added. Then the reaction mixture was stirred at room temperature until the reaction was complete (monitored by TLC). The reaction was quenched by the addition of saturated Na_2SO_3 aqueous solution (3.0 mL). The aqueous layer was extracted with ethyl acetate. The organic layer was washed with brine, dried over Na_2SO_4, filtered and then concentrated. The residue was purified by silica gel column chromatography (ethyl acetate/petroleum ether = 1/20, v/v) to afford **2q** (23.2 mg, 74% yield, 89% ee).

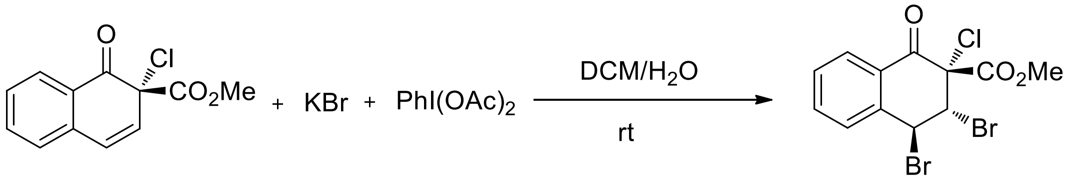

2t　　**3c**

To a solution of **2t** (47.3 mg, 0.2 mmol) in DCM (1.0 mL) and water (1.0 mL), KBr (71.4 mg, 0.6 mmol) and PhI(OAc)$_2$ (88.2 mg, 0.2 mmol) were added successively.

Then the reaction mixture was stirred at room temperature until the reaction was complete (monitored by TLC). The reaction was quenched by the addition of saturated $NaHCO_3$ aqueous solution (3.0 mL). The aqueous layer was extracted with DCM. The organic layer was washed with brine, dried over Na_2SO_4, filtered and then concentrated. The residue was purified by silica gel column chromatography (ethyl acetate/petroleum ether = 1/40, v/v) to afford the product.

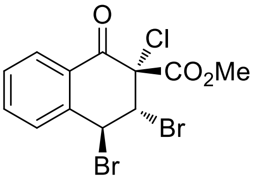

(2*R*,3*S*,4*S*)-Methyl 3,4-dibromo-2-chloro-1-oxo-1,2,3,4-tetrahydronaphthalene-2-carboxylate (3c)

White solid (67.1 mg, 85% yield, 0.2 mmol scale). Analytical data for **3c**: $[\alpha]_D^{20}$ = 26.1 (c = 1.0 $CHCl_3$, 88% ee); ^{1}H NMR (400 MHz, $CDCl_3$) δ 3.95 (s, 3H), 5.33 (d, *J* = 9.2 Hz, 1H), 5.69 (d, *J* = 9.2 Hz, 1H), 7.52 (t, *J* = 7.6 Hz, 1H), 7.72 (t, *J* = 7.2 Hz, 1H), 7.87 (d, *J* = 8.0 Hz, 1H), 8.06 (d, *J* = 7.6 Hz, 1H); ^{13}C NMR (100 MHz, $CDCl_3$) δ 50.6, 54.5, 56.5, 75.2, 127.2, 128.6, 129.6, 132.0, 135.7, 139.2, 164.6, 184.3; IR (film) 1737, 1698, 1274 cm^{-1}; HRMS (ESI) exact mass calcd for $C_{12}H_{13}Br_2ClNO_3^+$ (M+NH_4) requires *m/z* 411.8945. Found *m/z* 411.8943. The enantiomeric ratio was determined by Daicel Chiralpak OD-H (25 cm), Hexane / IPA = 90 / 10, 1.0 mL/min, λ = 254 nm, t (major) = 8.88 min, t (minor) = 10.72 min.

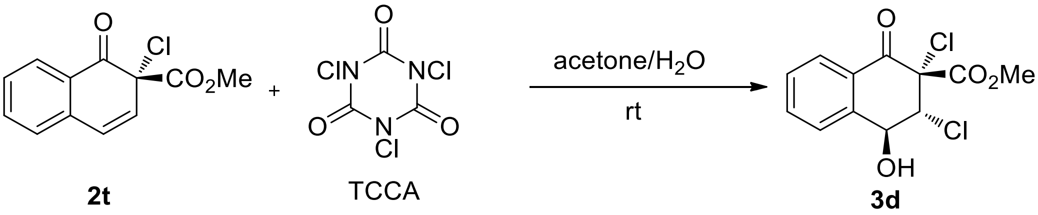

To a solution of **2t** (46.4 mg, 0.2 mmol) in acetone (1.0 mL) and water (0.2 mL), TCCA (46.4 mg, 0.2 mmol) was added. Then the reaction mixture was stirred at room temperature until the reaction was complete (monitored by TLC). The reaction was quenched by the addition of saturated Na_2SO_3 aqueous solution (3 mL). The aqueous layer was extracted with ethyl acetate. The organic layer was washed with brine, dried

over Na_2SO_4, filtered and then concentrated. The residue was purified by silica gel column chromatography (ethyl acetate/petroleum ether = 1/6, v/v) to afford the product.

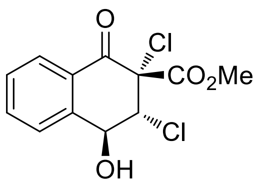

(2*R*,3*R*,4*S*)-Methyl 2,3-dichloro-4-hydroxy-1-oxo-1,2,3,4-tetrahydronaphthalene-2-carboxylate (3d)

White foam (39.8 mg, dr = 11:1, 70% yield, 0.2 mmol scale). Analytical data for **3d**: $[\alpha]_D^{20}$ = 18.1 (c = 1.0 $CHCl_3$, 86% ee); ^1H NMR (400 MHz, $CDCl_3$) δ 3.17 (d, J = 4.8 Hz, 1H), 3.95 (s, 3H), 4.94 (d, J = 9.2 Hz, 1H), 5.17 (dd, J = 9.2, 4.8 Hz, 1H), 7.53 (t, J = 7.6 Hz, 1H), 7.76 (td, J = 7.2, 1.2 Hz, 1H), 7.87 (d, J = 8.0 Hz, 1H), 8.10 (dd, J = 8.0, 1.2 Hz, 1H); ^{13}C NMR (100 MHz, $CDCl_3$) δ 54.5, 65.8, 70.2, 74.7, 127.1, 128.9, 129.1, 135.8, 136.0, 141.0, 164.9, 185.0; IR (film) 3498, 1749, 1694, 1279, 1243 cm^{-1}; HRMS (ESI) exact mass calcd for $C_{12}H_{14}Cl_2NO_4^+$ (M+NH_4) requires *m/z* 306.0294. Found *m/z* 306.0294. The enantiomeric ratio was determined by Daicel Chiralpak AD-H (25 cm), Hexane / IPA = 80 / 3, 0.83 mL/min, λ = 254 nm, t (major) = 48.66 min, t (minor) = 63.02 min.

3.1 Mechanistic Investigations

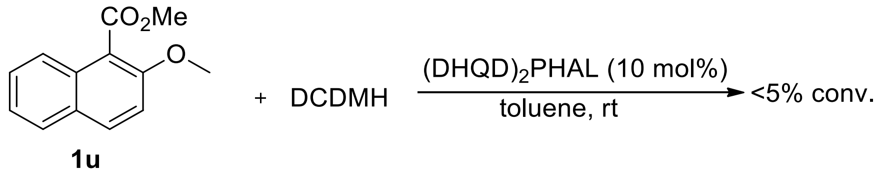

To a Schlenk tube, DCDMH (47.3 mg, 0.24 mmol), $(DHQD)_2PHAL$ (15.6 mg, 0.02 mmol) and toluene (1.0 mL) were added. Then **1u** (43.2 mg, 0.2 mmol) was added in one portion. Very low conversion of **1u** was observed and no product **2a** was detected after the reaction mixture was stirred for 20 h at rt.

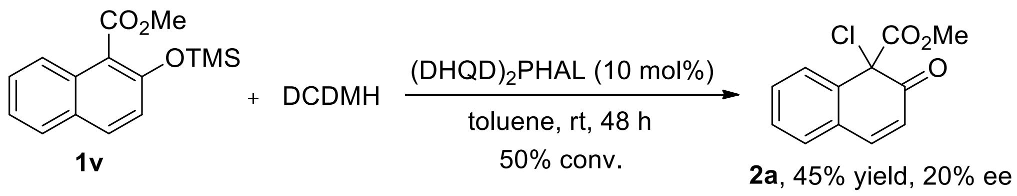

To a Schlenk tube, DCDMH (78.8 mg, 0.4 mmol), $(DHQD)_2PHAL$ (15.6 mg, 0.02 mmol) and toluene (1.0 mL) were added. Then **1v** (54.8 mg, 0.2 mmol) was added in one portion. After stirred for 48 h at rt, the reaction was quenched by the addition of saturated Na_2SO_3 aqueous solution (3.0 mL). The organic layer was extracted with ethyl acetate, washed with brine, dried over Na_2SO_4, filtered and then concentrated. The residue was purified by silica gel column chromatography (ethyl acetate/petroleum ether = 1/20, v/v) to afford the product **2a** (21.5 mg, 45% yield, 20% ee).

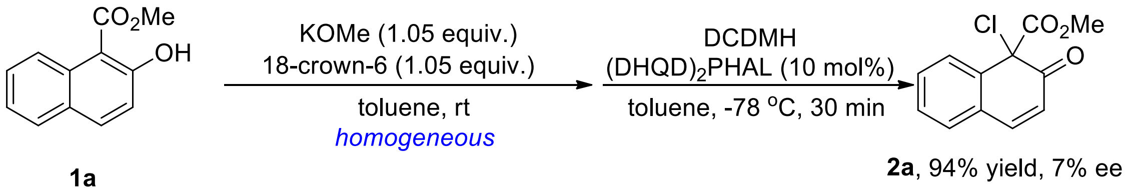

To a flame-dried Schlenk tube, **1a** (60.6 mg, 0.3 mmol), 18-crown-6 (82.2 mg, 0.32 mmol) and toluene (2.0 mL) were added. Then potassium methoxide (22.2 mg, 0.32 mmol) was added. After stirred for 0.5 h at rt, a homogeneous solution was formed. To another Schlenk tube, DCDMH (71.0 mg, 0.36 mmol), $(DHQD)_2PHAL$ (23.4 mg, 0.03 mmol) and toluene (1.0 mL) were added. After stirred for 10 min at -78 ^{o}C, the previously prepared homogeneous solution of **1a** was added. The reaction was quenched by the addition of saturated Na_2SO_3 aqueous solution (3.0 mL) after stirred for 30 min at -78 ^{o}C. The organic layer was extracted with ethyl acetate, washed with brine, dried over Na_2SO_4, filtered and then concentrated. The residue was purified by silica gel column chromatography (ethyl acetate/petroleum ether = 1/20, v/v) to afford the product **2a** (66.3 mg, 94% yield, 7% ee).

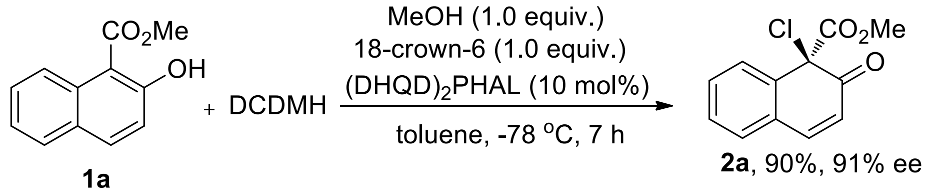

To a Schlenk tube, DCDMH (23.5 mg, 0.12 mmol), $(DHQD)_2PHAL$ (7.8 mg, 0.01

mmol), methanol (4.0 μL, 3.5 mg), 18-crown-6 (26.3 mg, 0.1 mmol) and toluene (1.0 mL) were added successively. After stirred for 10 min at -78 °C, **1a** (20.2 mg, 0.1 mmol) was added. Then the reaction mixture was stirred at -78 °C until the reaction was complete (monitored by TLC). The reaction was quenched by the addition of saturated Na_2SO_3 aqueous solution (3.0 mL) after stirred for 30 min at -78 °C. The organic layer was extracted with ethyl acetate, washed with brine, dried over Na_2SO_4, filtered and then concentrated. The residue was purified by silica gel column chromatography (ethyl acetate/petroleum ether = 1/20, v/v) to afford the product **2a** (21.5 mg, 90% yield, 91% ee).

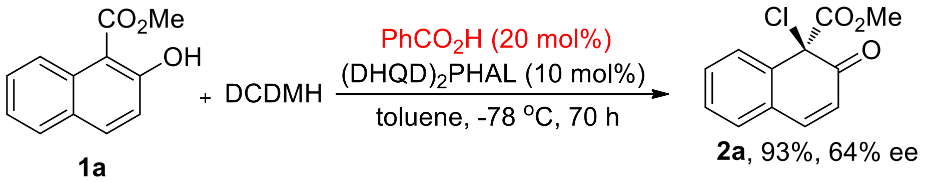

To a Schlenk tube, DCDMH (70.9 mg, 0.36 mmol), $(DHQD)_2PHAL$ (23.4 mg, 0.03 mmol), benzoic acid (7.3 mg, 0.06 mmol) and toluene (2.0 mL) were added successively. After stirred for 10 min at -78 °C, **1a** (60.6 mg, 0.3 mmol) was added. Then the reaction mixture was stirred at -78 °C until the reaction was complete (monitored by TLC). The reaction was quenched by the addition of saturated Na_2SO_3 aqueous solution (3.0 mL). The organic layer was extracted with ethyl acetate, washed with brine, dried over Na_2SO_4, filtered and then concentrated. The residue was purified by silica gel column chromatography (ethyl acetate/petroleum ether = 1/20, v/v) to afford the product **2a** (65.8 mg, 93% yield, 64% ee).

4. Crystal data

In order to determine the absolute configuration of the products, the crystal of enantiopure **2d** was obtained by slow evaporation in hexane and EA and a single crystal X-ray analysis determined its configuration as *R* (Fig. 1) (CCDC 1048302).

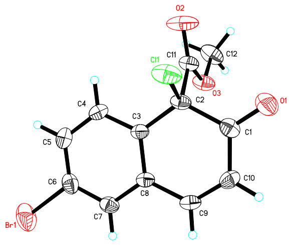

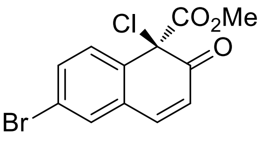

(*R*)-**2d**

Fig. 1

Table 1. Crystal data and structure refinement for (*R*)-2d.

Identification code	cd21437
Empirical formula	C12 H8 Br Cl O3
Formula weight	315.54
Temperature	293(2) K
Wavelength	0.71073 A
Crystal system, space group	Orthorhombic, P2(1)2(1)2(1)
Unit cell dimensions	a = 7.4500(9) A alpha = 90 deg.
	b = 7.7960(9) A beta = 90 deg.
	c = 21.193(3) A gamma = 90 deg.
Volume	1230.9(3) A^3
Z, Calculated density	4, 1.703 Mg/m^3
Absorption coefficient	3.548 mm^-1
F(000)	624
Crystal size	0.211 x 0.165 x 0.123 mm
Theta range for data collection	2.78 to 26.00 deg.
Limiting indices	-9<=h<=9, -9<=k<=8, -26<=l<=25
Reflections collected / unique	7410 / 2418 [R(int) = 0.0475]
Completeness to theta = 26.00	99.9 %
Absorption correction	Empirical
Max. and min. transmission	1.00000 and 0.45967
Refinement method	Full-matrix least-squares on F^2
Data / restraints / parameters	2418 / 0 / 156
Goodness-of-fit on F^2	1.026

Final R indices [I>2sigma(I)]	R1 = 0.0386, wR2 = 0.0941
R indices (all data)	R1 = 0.0494, wR2 = 0.0988
Absolute structure parameter	0.007(12)
Extinction coefficient	0.0013(14)
Largest diff. peak and hole	0.530 and -0.519 e.A^-3

In order to determine the absolute configuration of **2t**, the crystal of enantiopure **2t** was obtained by slow evaporation in hexane and Et_2O and a single crystal X-ray analysis determined its configuration as *R* (Fig. 2) (CCDC 1048128).

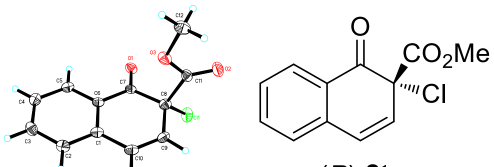

(*R*)-**2t**

Fig. 2

Table 2. Crystal data and structure refinement for (*R*)-**2t**.

Identification code	dm14320	
Empirical formula	C12 H9 Cl O3	
Formula weight	236.64	
Temperature	133(2) K	
Wavelength	0.71073 Å	
Crystal system	Orthorhombic	
Space group	P 21 21 21	
Unit cell dimensions	a = 9.632(2) Å	a= 90°.
	b = 9.726(2) Å	b= 90°.
	c = 11.729(3) Å	g = 90°.
Volume	1098.7(4) $Å^3$	
Z	4	
Density (calculated)	1.431 Mg/m^3	
Absorption coefficient	0.335 mm^{-1}	
F(000)	488	

Crystal size	0.211 x 0.176 x 0.123 mm^3
Theta range for data collection	2.721 to 25.494 °.
Index ranges	-11<=h<=11, -11<=k<=11, -11<=l<=14
Reflections collected	7270
Independent reflections	2040 [R(int) = 0.0550]
Completeness to theta = 25.242 °	99.4 %
Absorption correction	Semi-empirical from equivalents
Max. and min. transmission	0.7457 and 0.4633
Refinement method	Full-matrix least-squares on F^2
Data / restraints / parameters	2040 / 0 / 147
Goodness-of-fit on F^2	1.073
Final R indices [I>2sigma(I)]	R1 = 0.0437, wR2 = 0.1154
R indices (all data)	R1 = 0.0471, wR2 = 0.1182
Absolute structure parameter	0.11(5)
Largest diff. peak and hole	0.432 and -0.233 e.$Å^{-3}$

5. References

1. (a) Bobrov, S.; Cai, C.; Katritzky, A. R.; Singh, S. K. *J. Org. Chem*. **2006**, *71*, 3364. (b) Aoyama, T.; Okutome, T.; Nakayama, T.; Yaegashi, T.; Matsui, R.; Nunomura, S.; Kurumi, Y.; Fujii, S. *Chem. Pharm. Bull*. **1985**, *33*, 1458. (c) Oguma, T.; Katsuki, T. *J. Am. Chem. Soc.* **2012**, *134*, 20017.

6. Copies of NMR spectra

Compound **1c**'s NMR Spectra

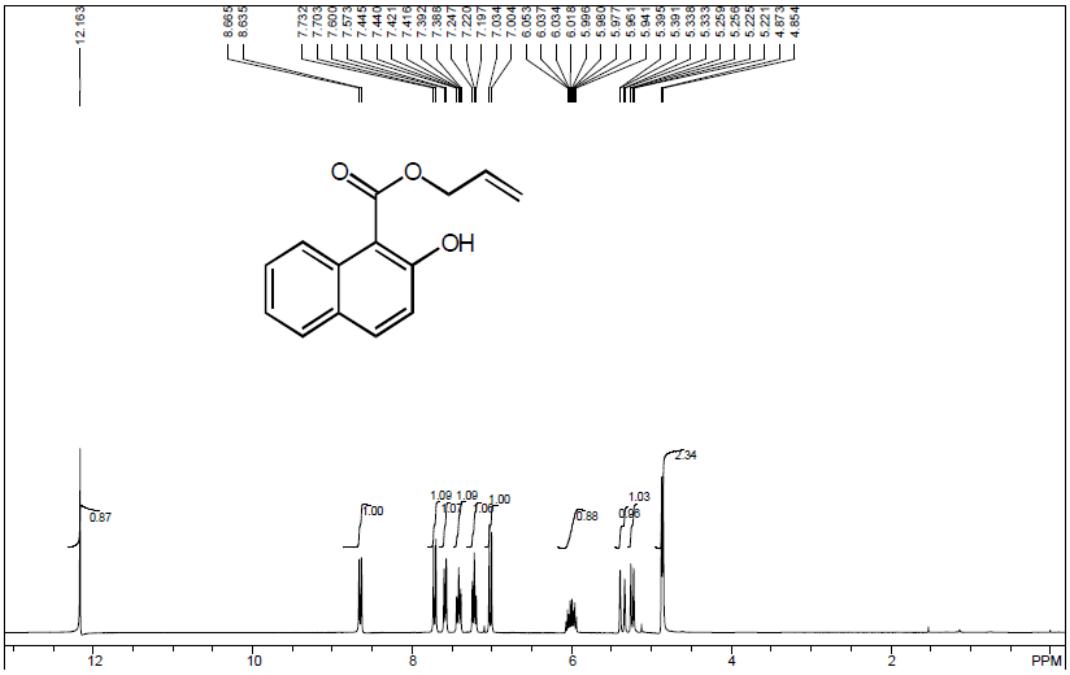

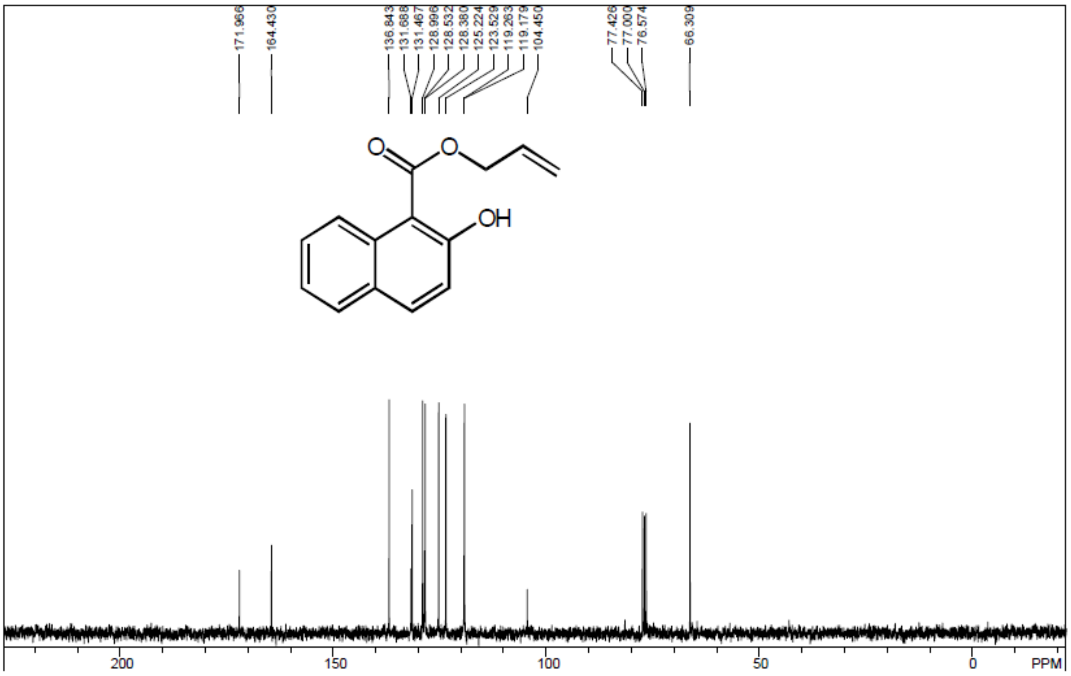

Compound **1f**'s NMR Spectra

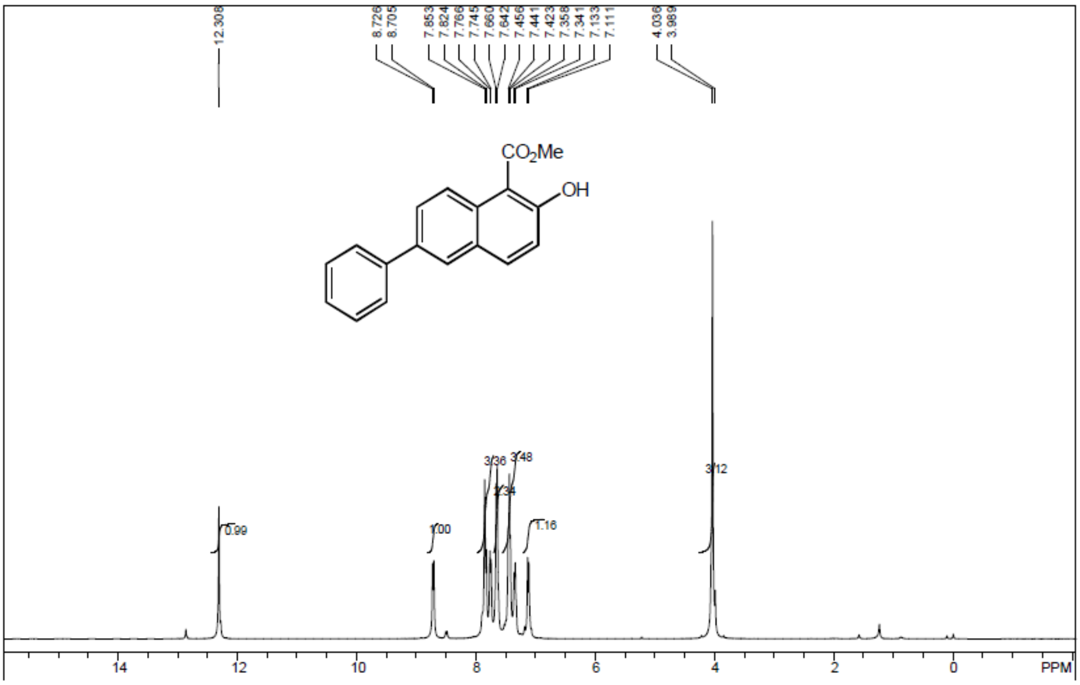

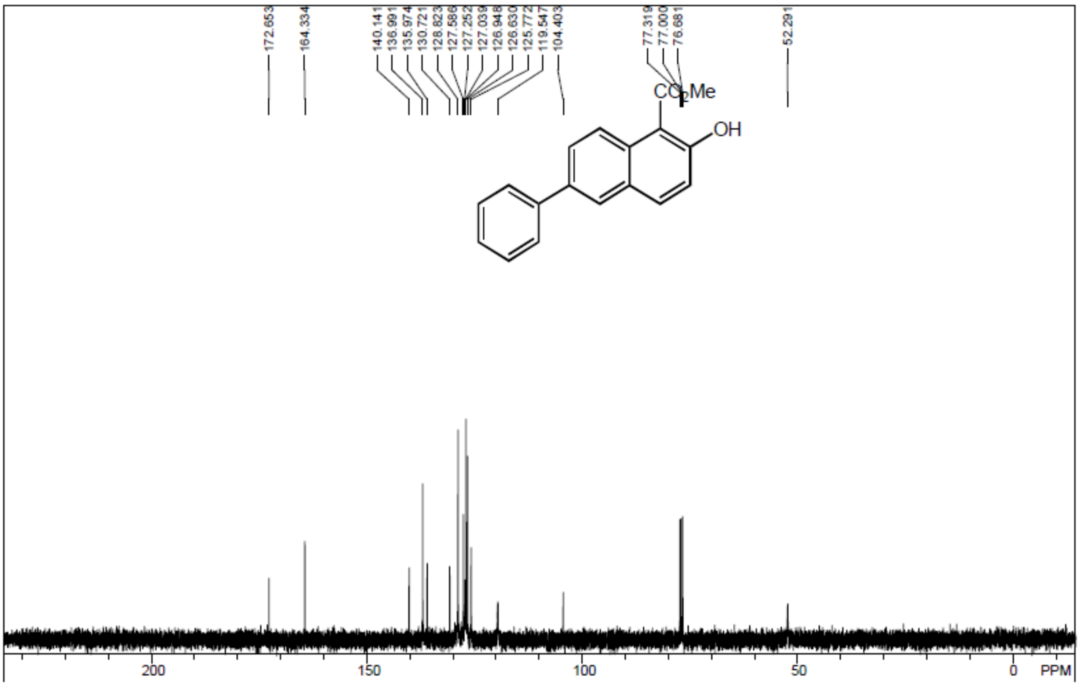

Compound **1g**’s NMR Spectra

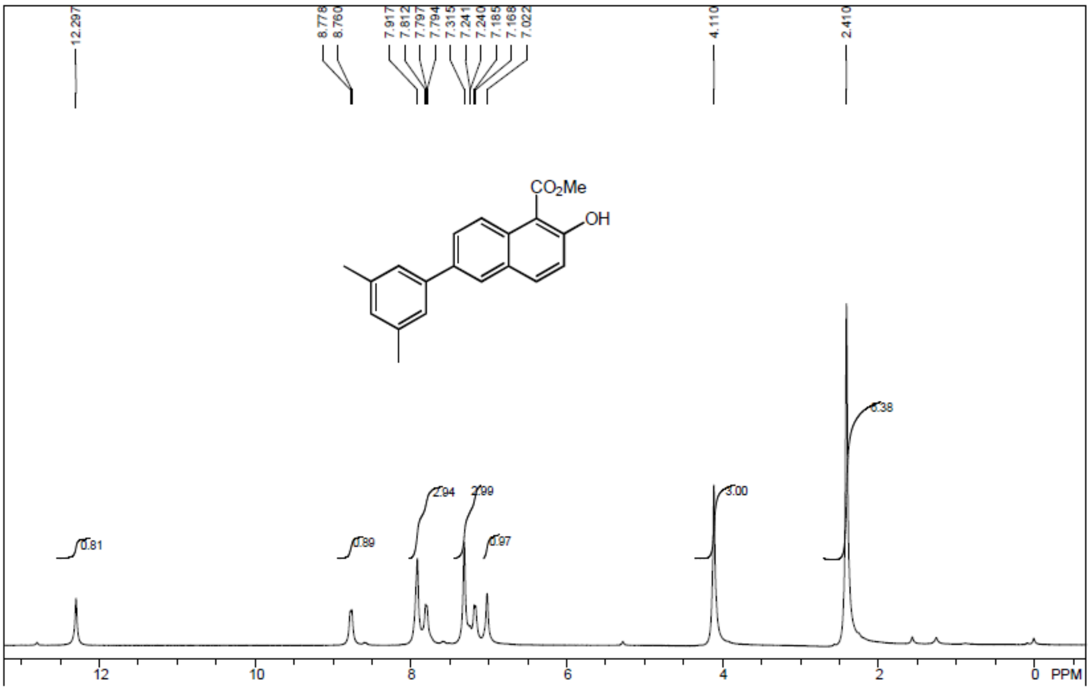

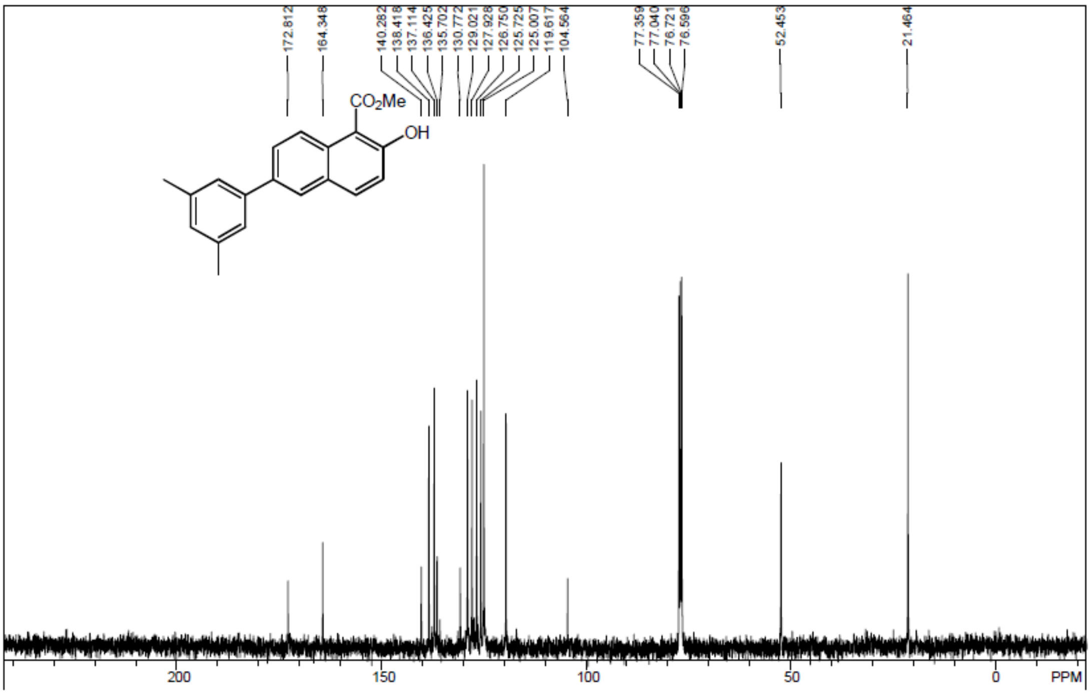

Compound **1h**'s NMR Spectra

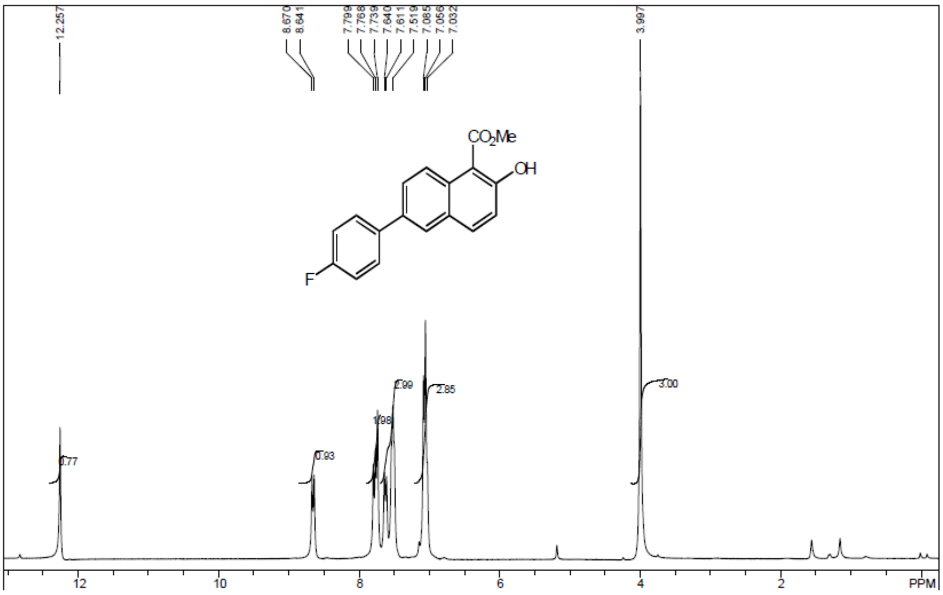

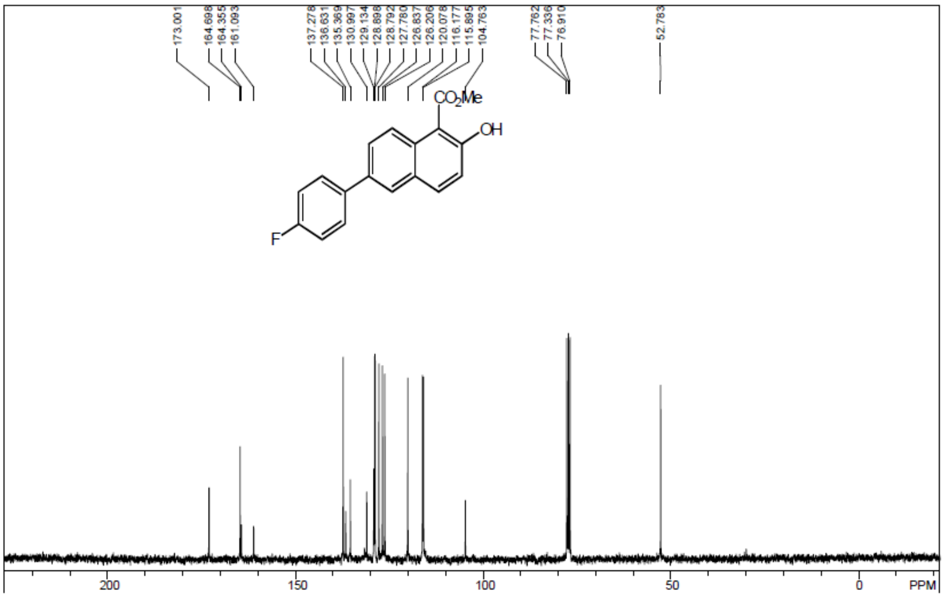

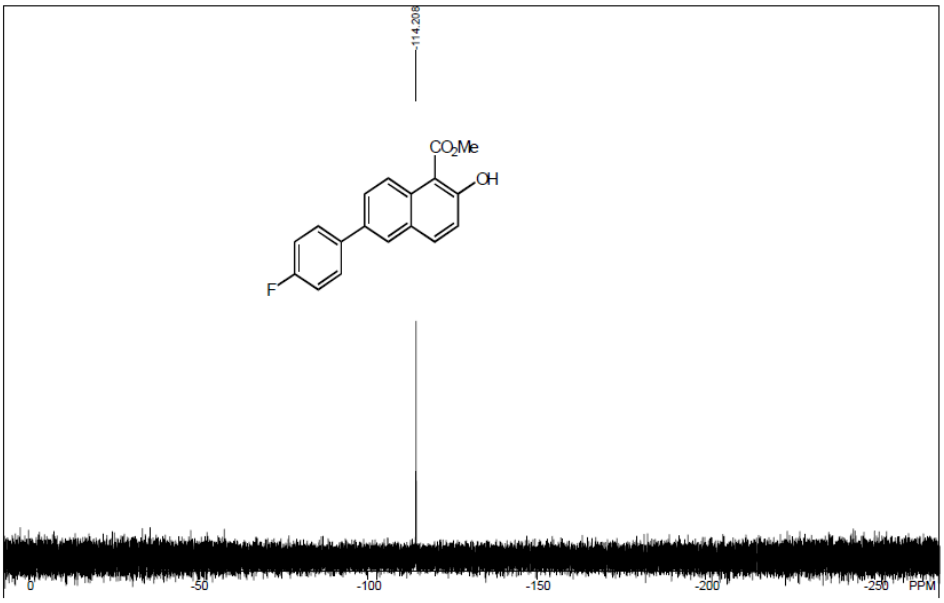

Compound **1i**'s NMR Spectra

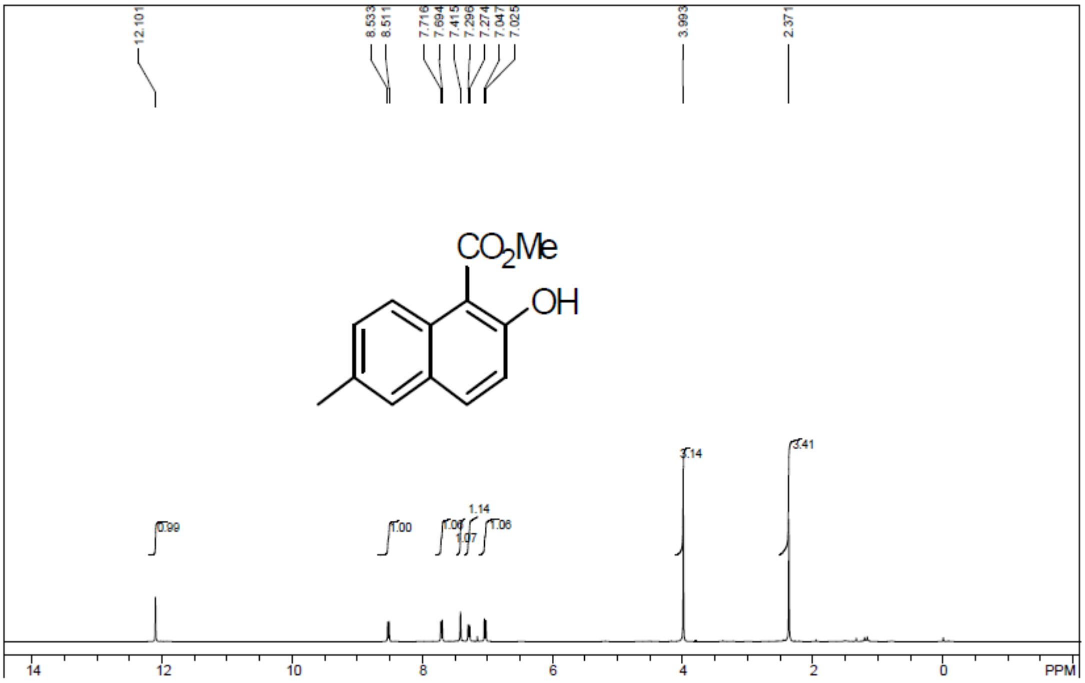

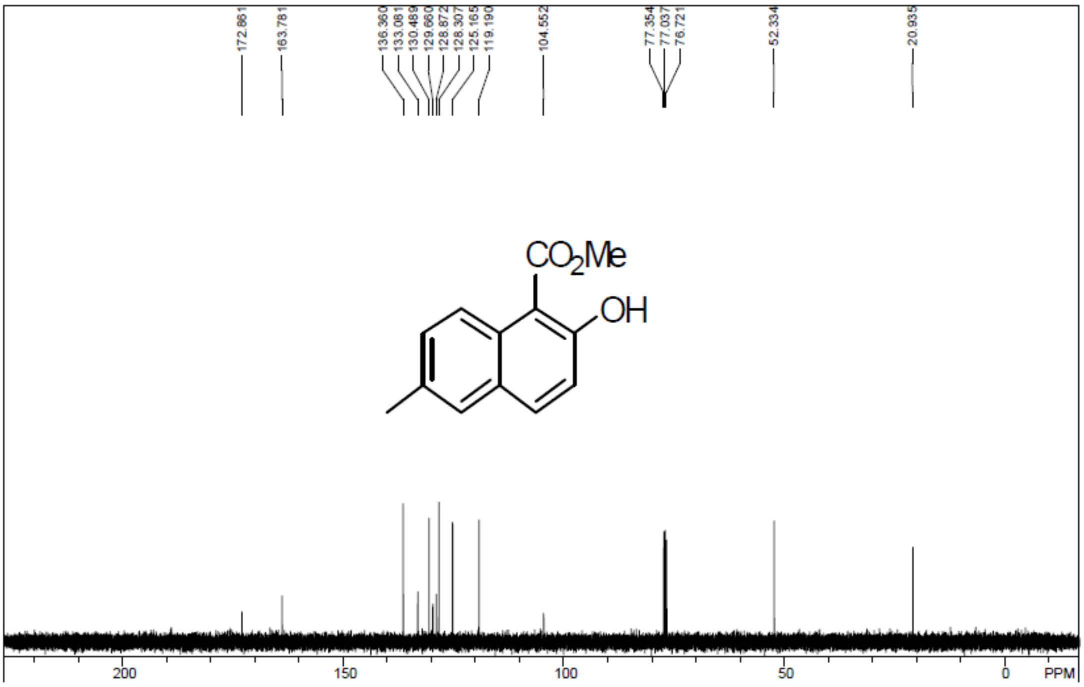

Compound **1j**'s NMR Spectra

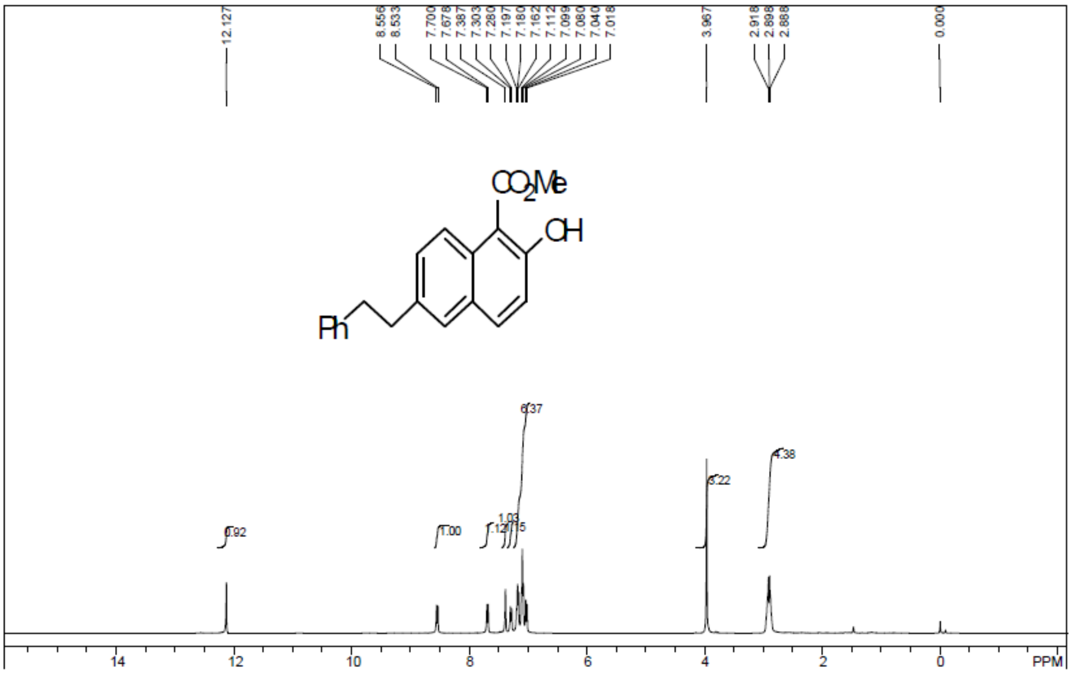

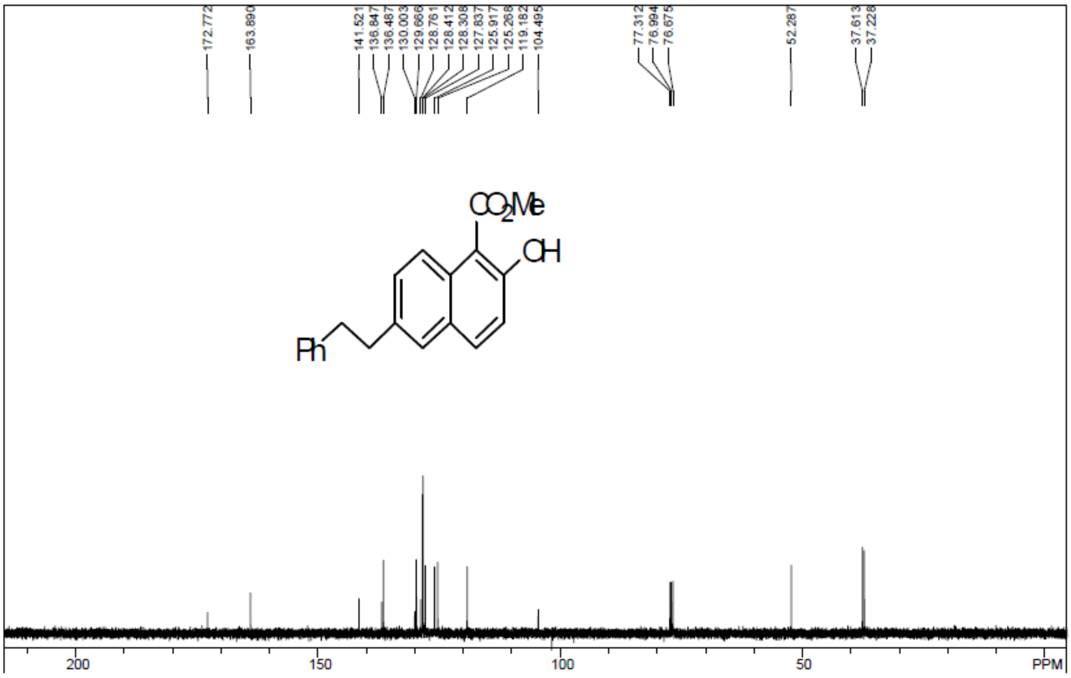

Compound **1k**'s NMR Spectra

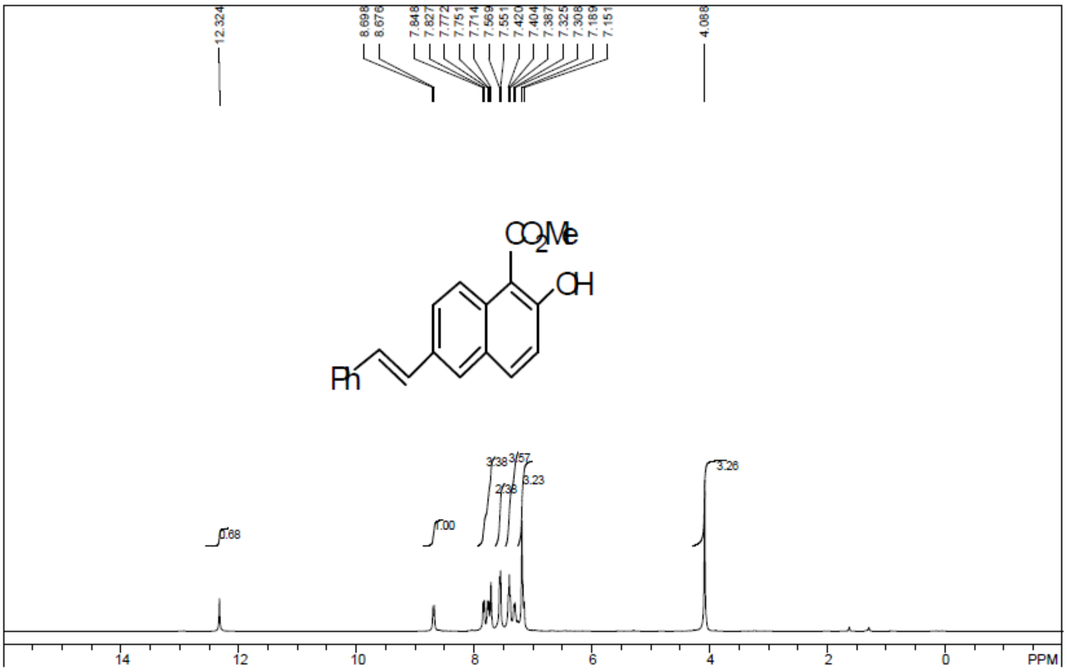

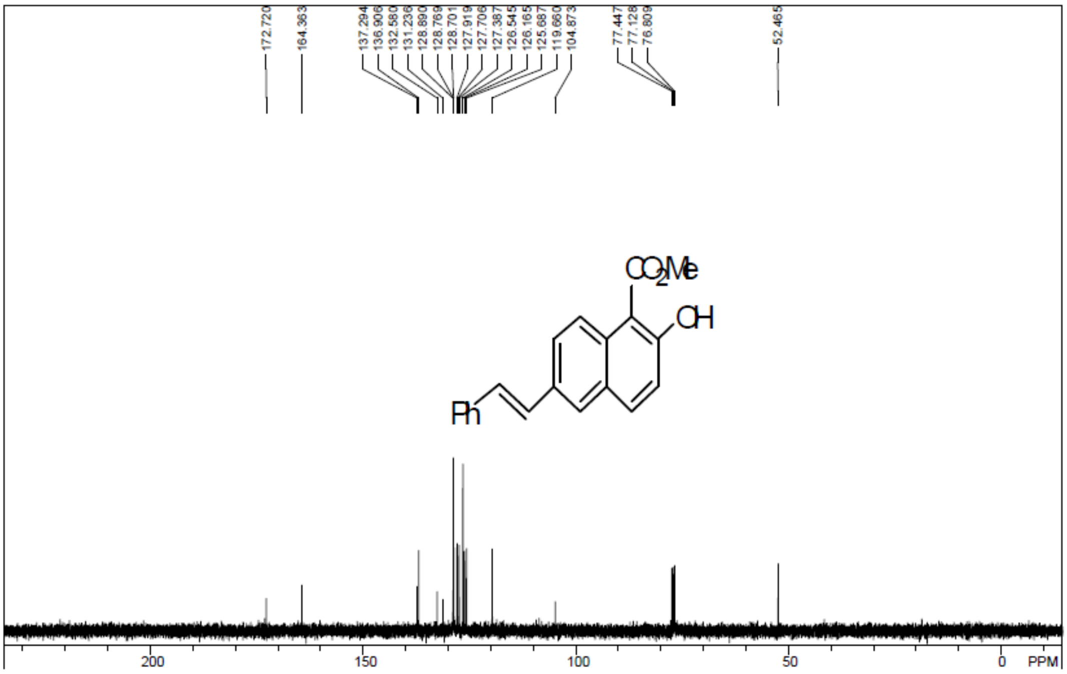

Compound **1l**'s NMR Spectra

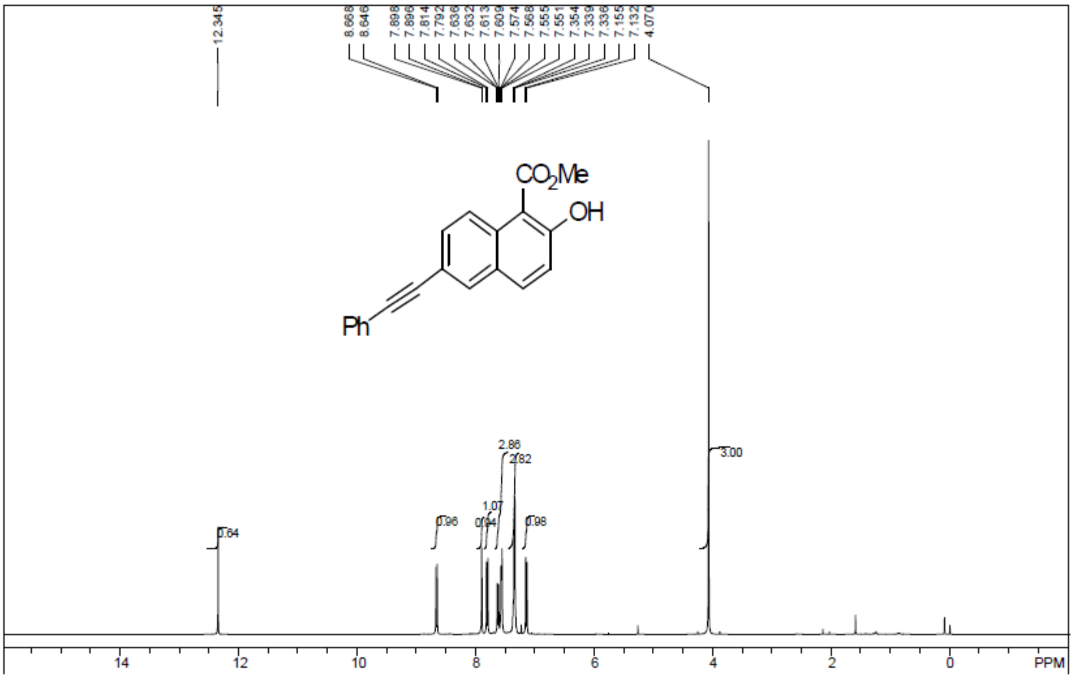

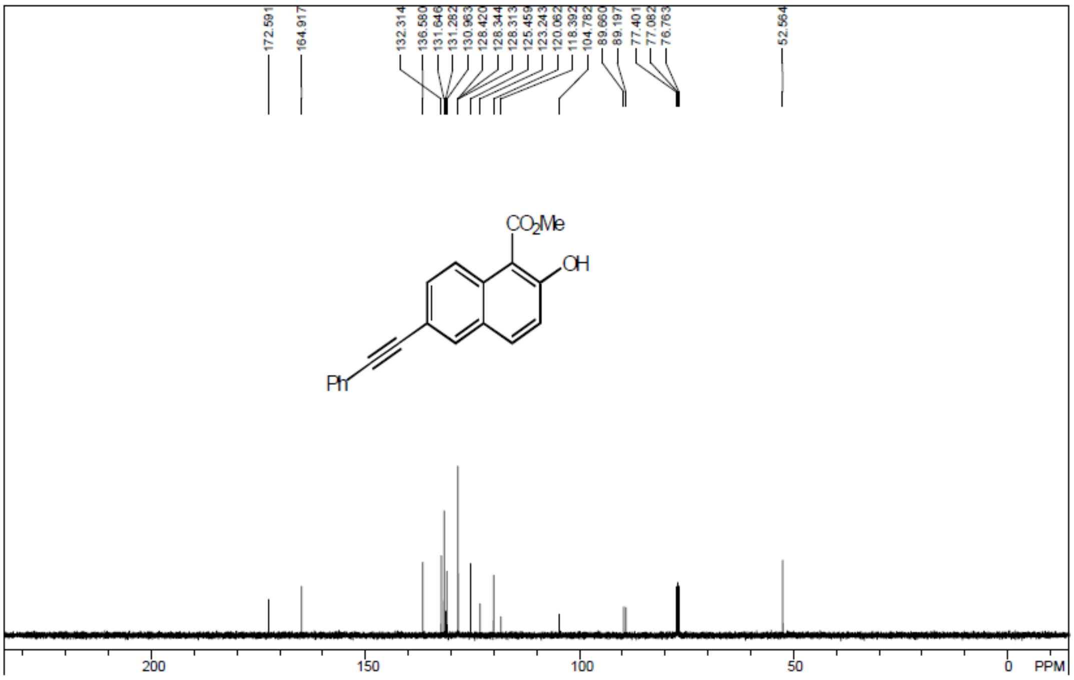

Compound **1m**'s NMR Spectra

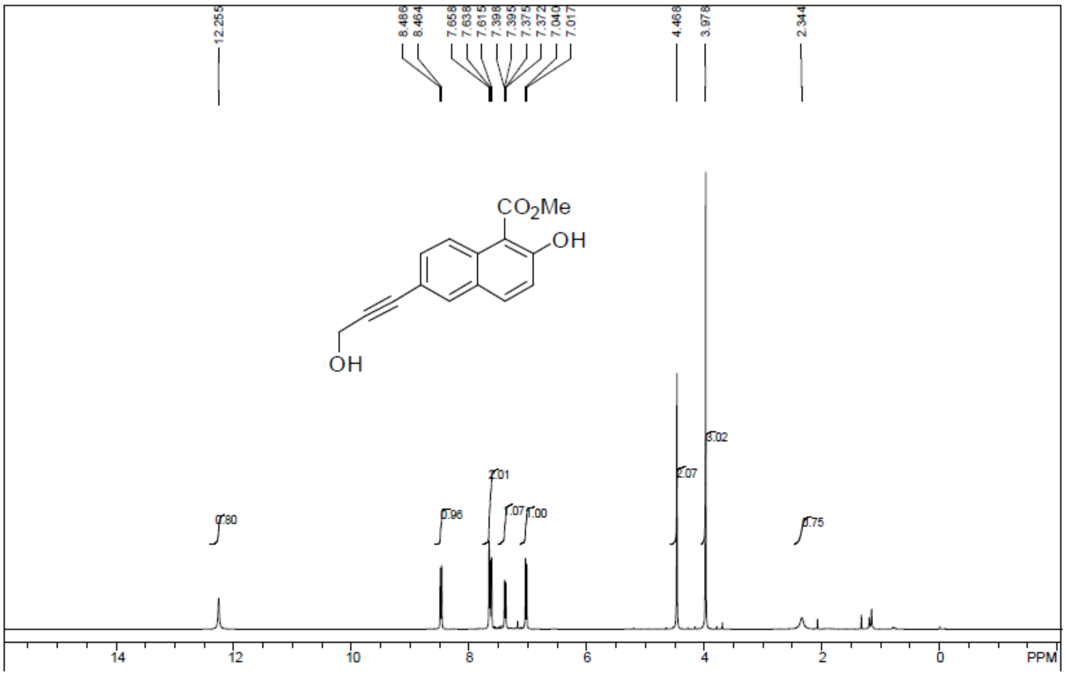

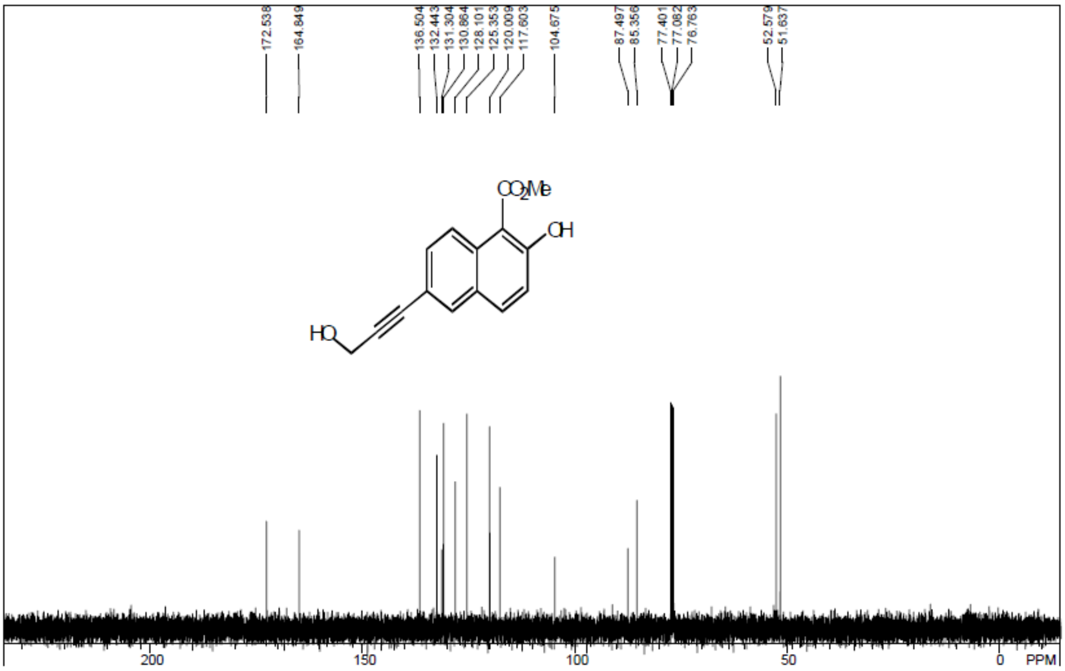

Compound **1n**'s NMR Spectra

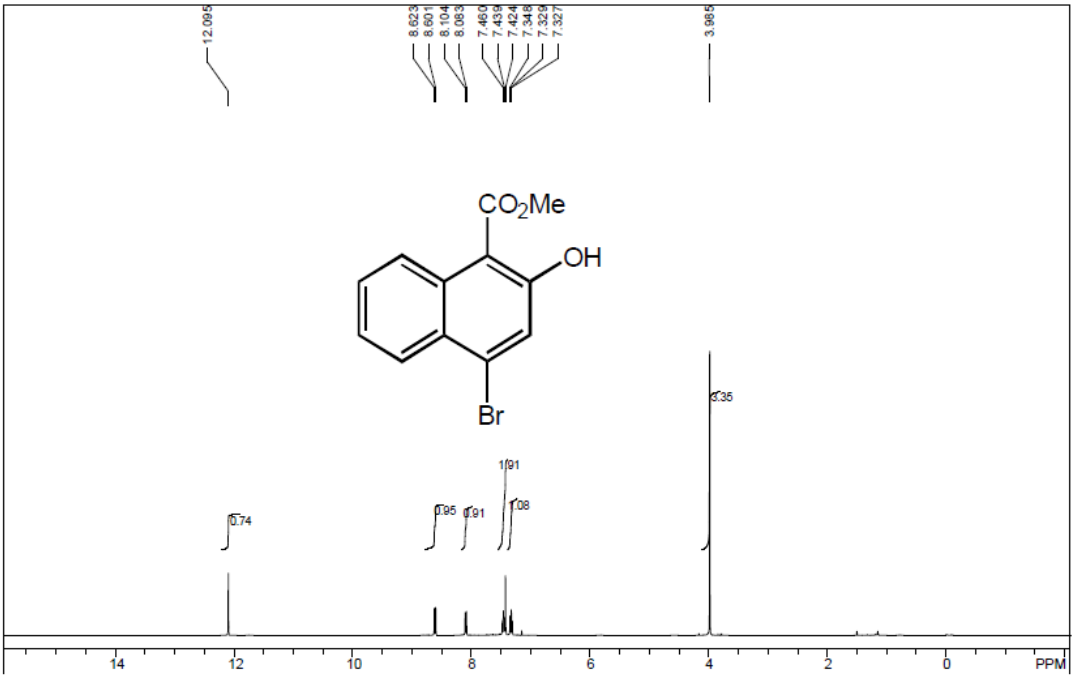

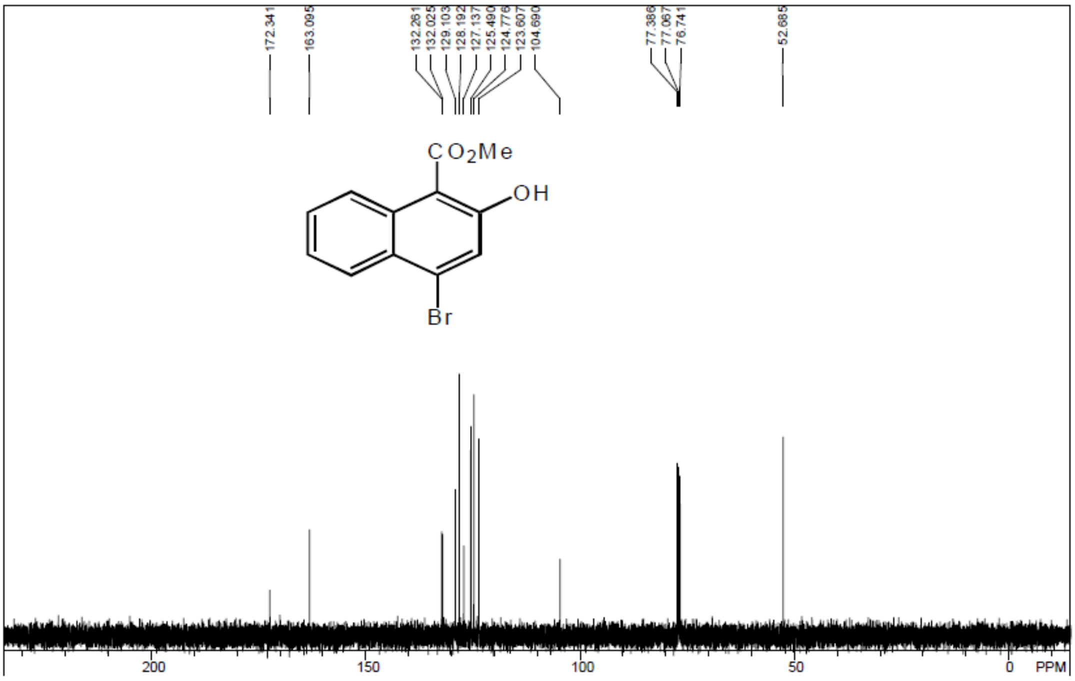

Compound **1o**'s NMR Spectra

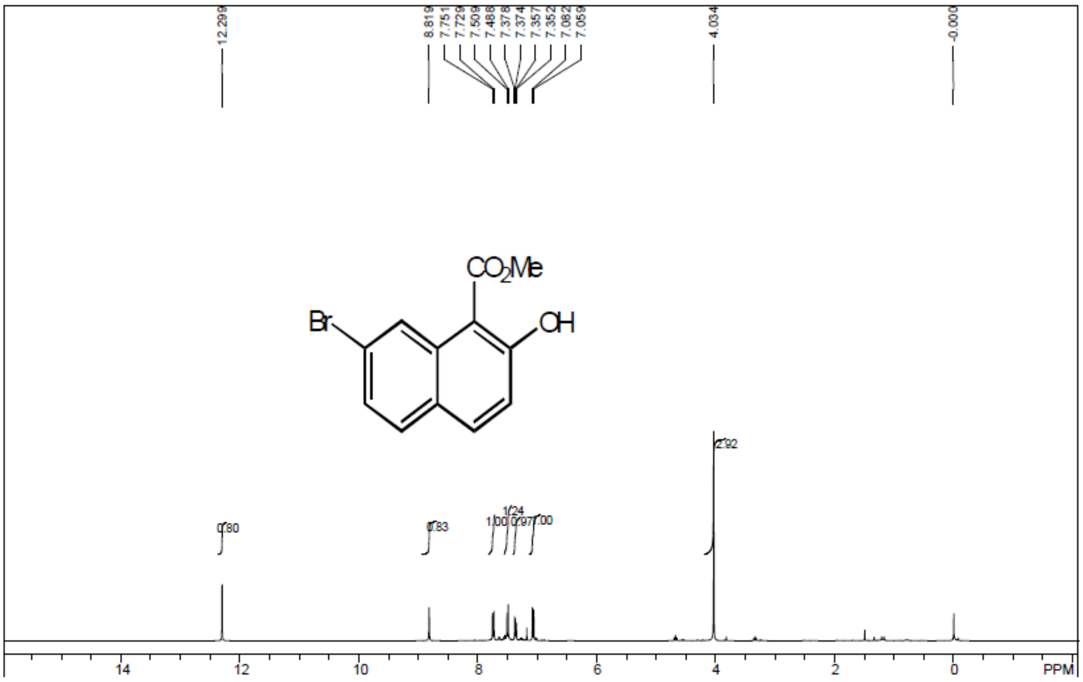

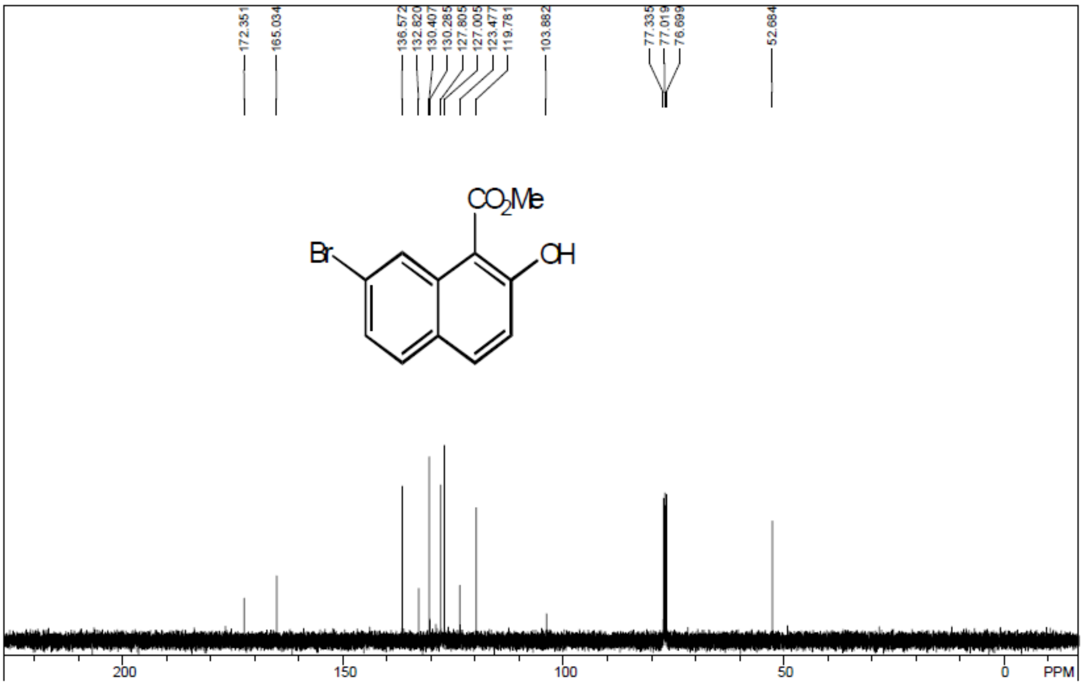

Compound **1p**'s NMR Spectra

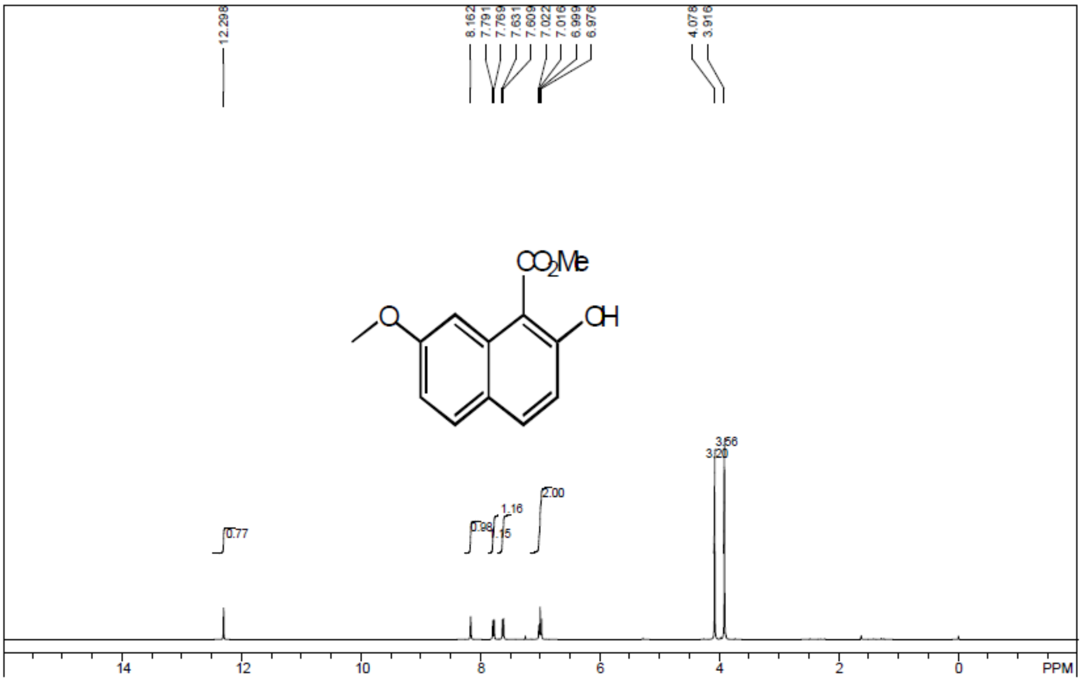

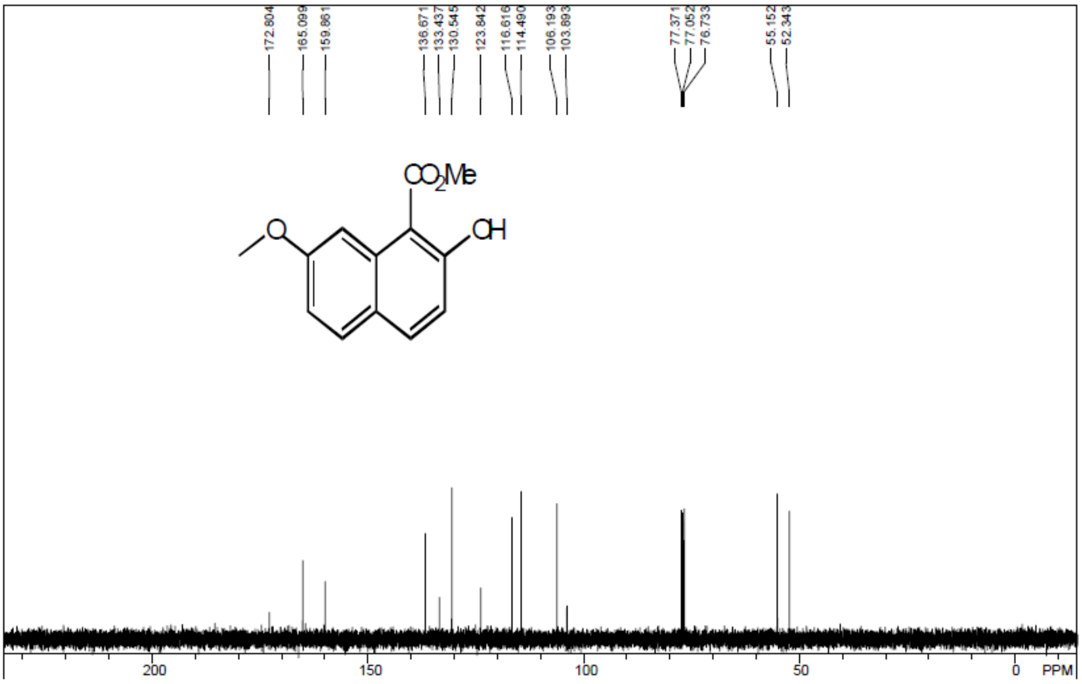

Compound **1q**'s NMR Spectra

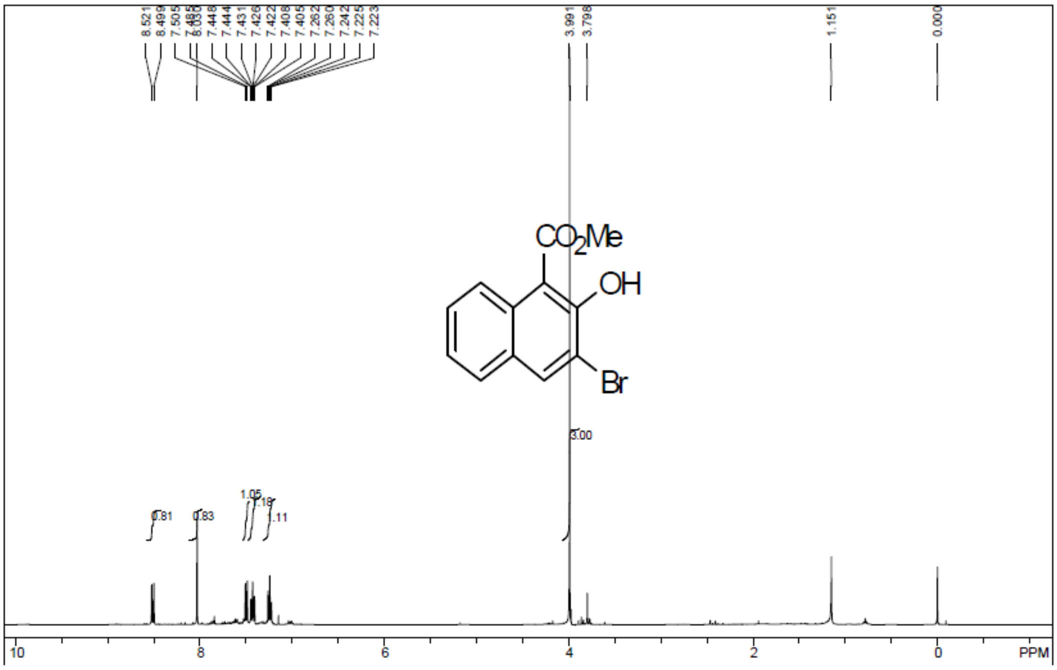

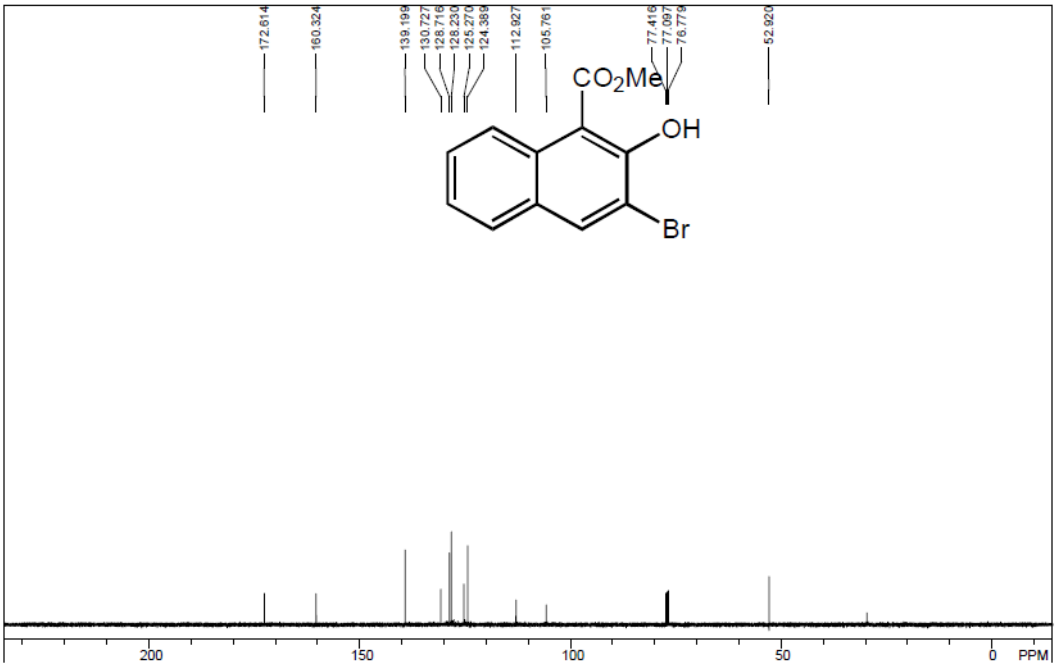

Compound **2a**'s NMR Spectra

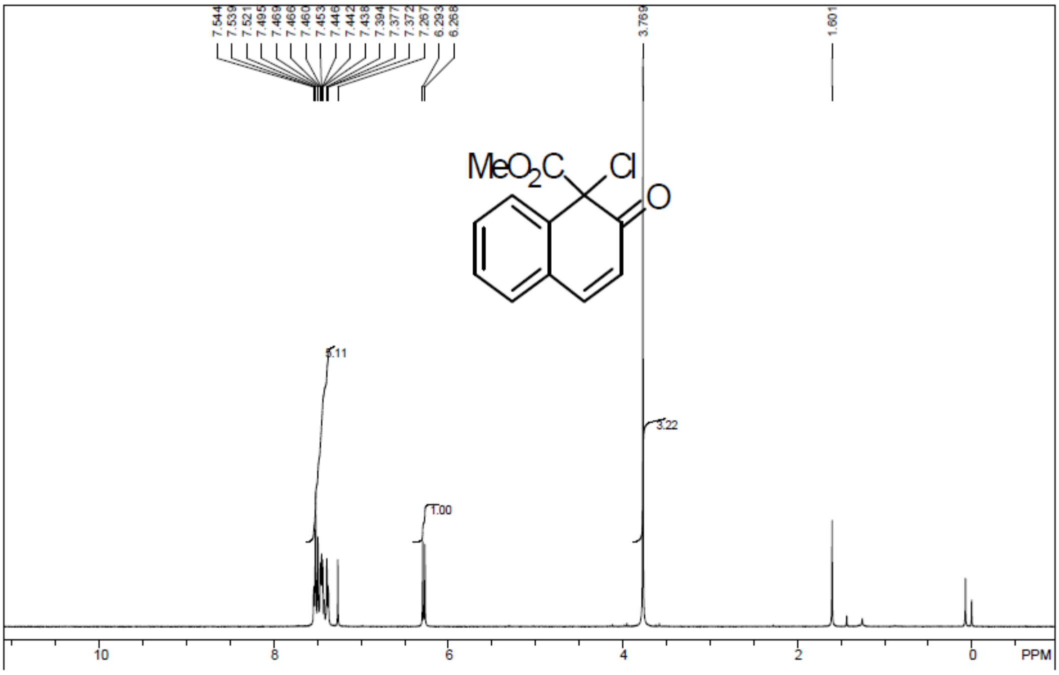

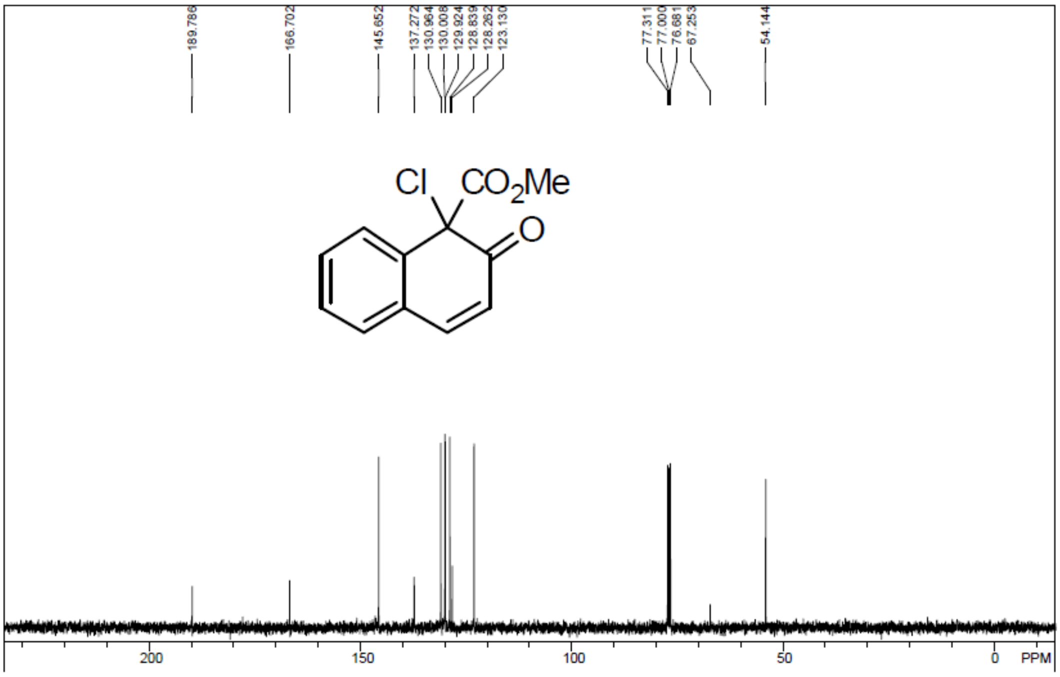

Compound **2b**’s NMR Spectra

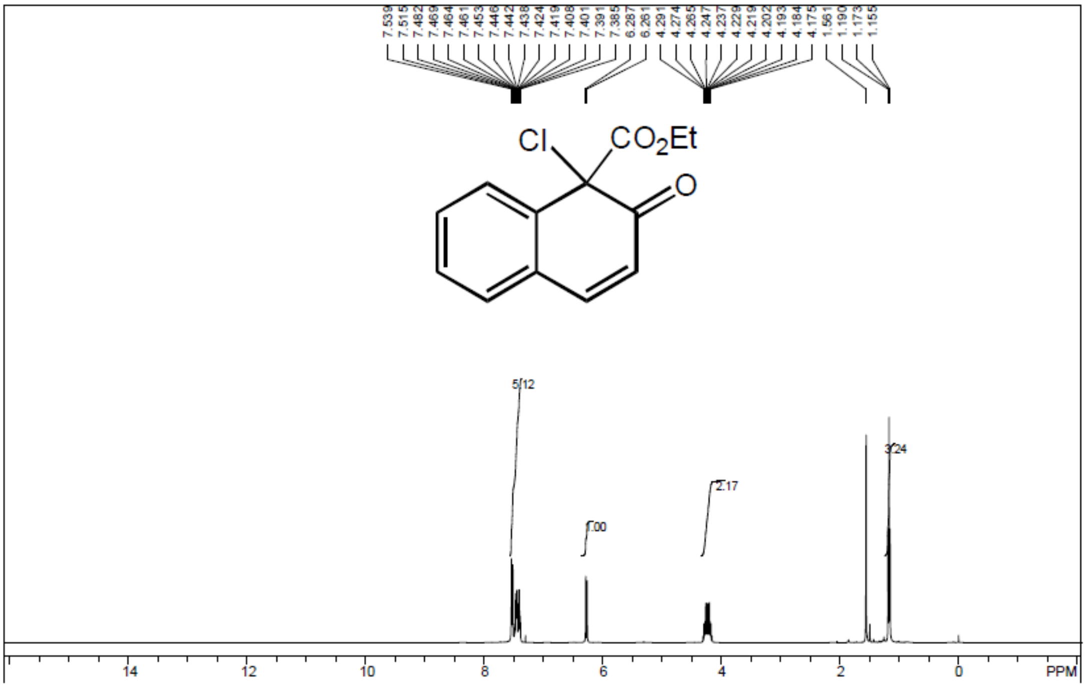

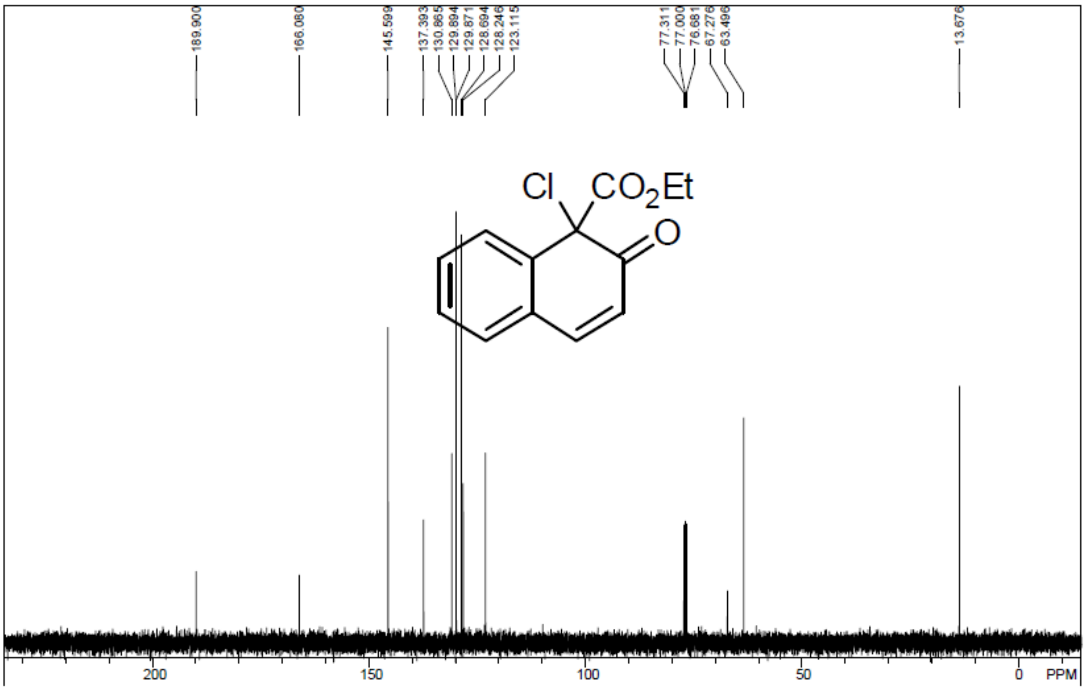

Compound **2c**'s NMR Spectra

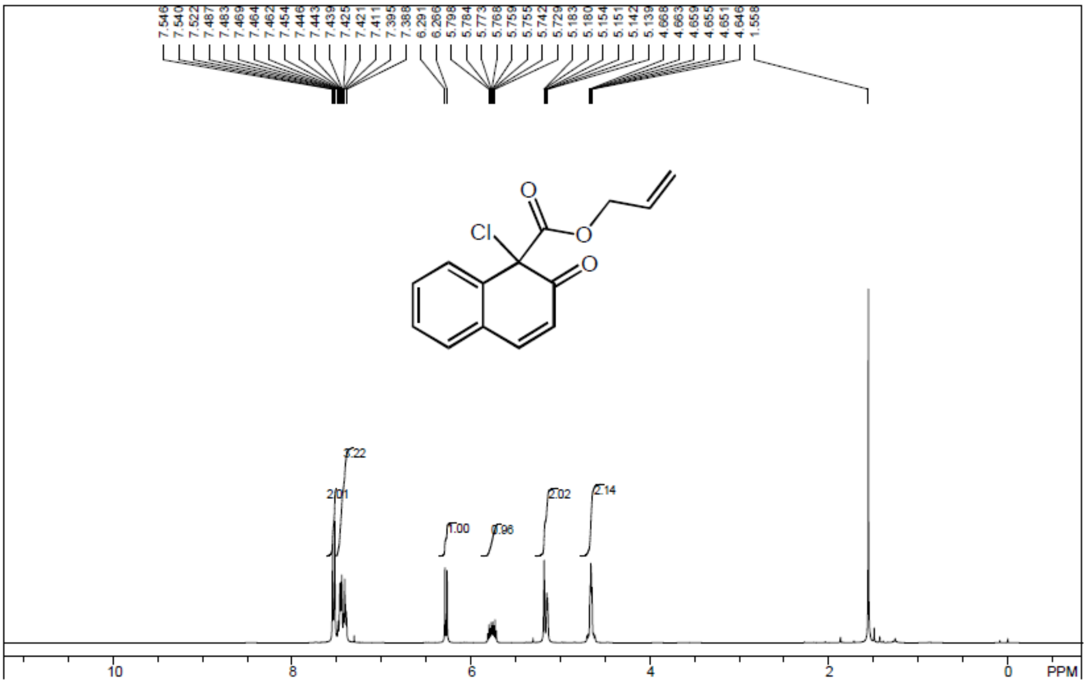

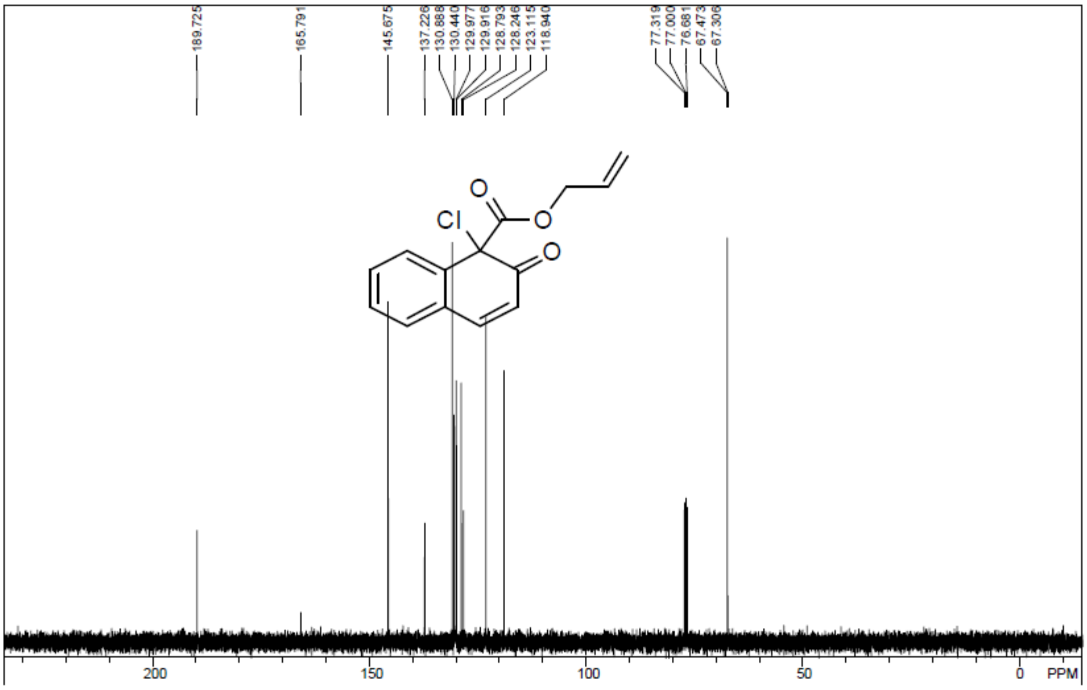

Compound **2d**'s NMR Spectra

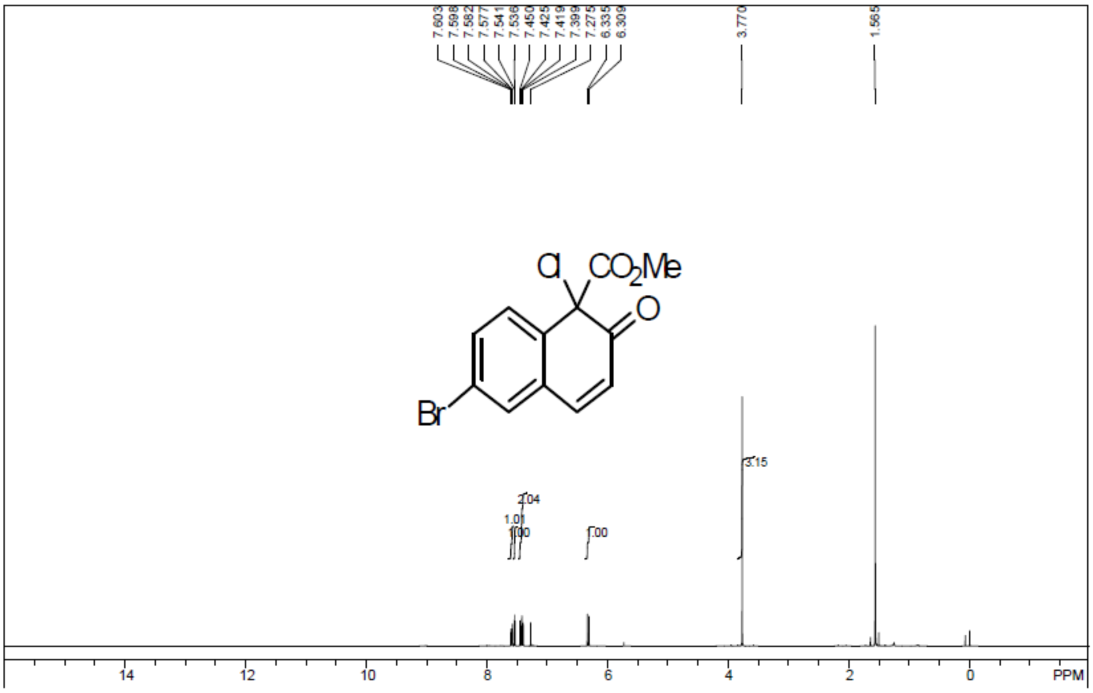

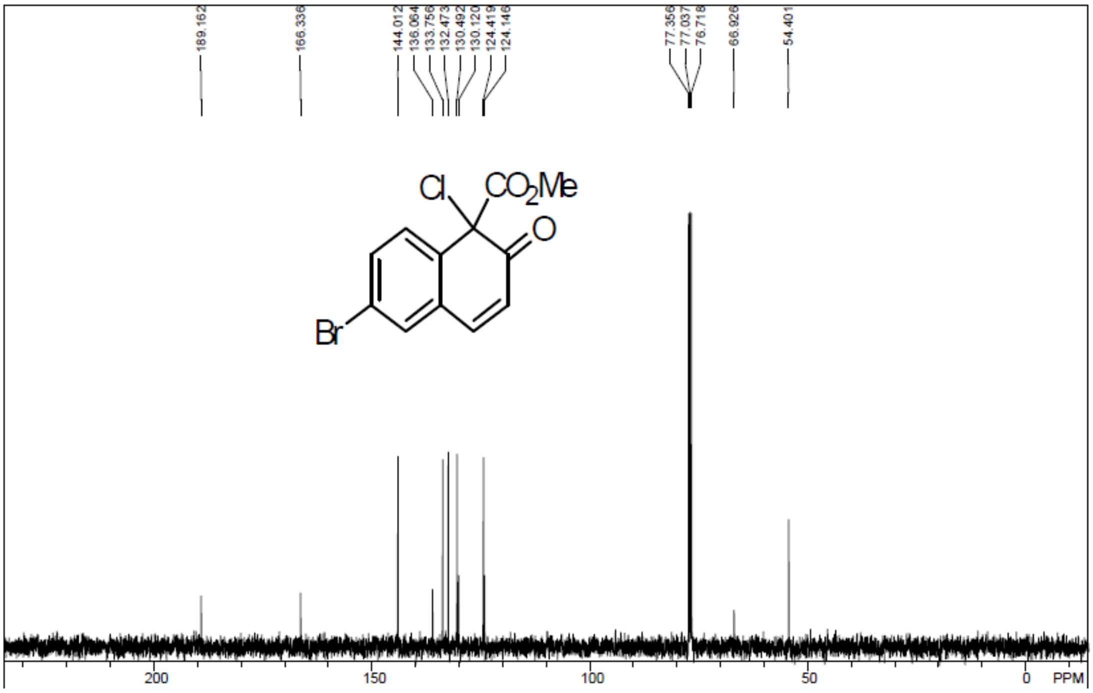

Compound **2e**'s NMR Spectra

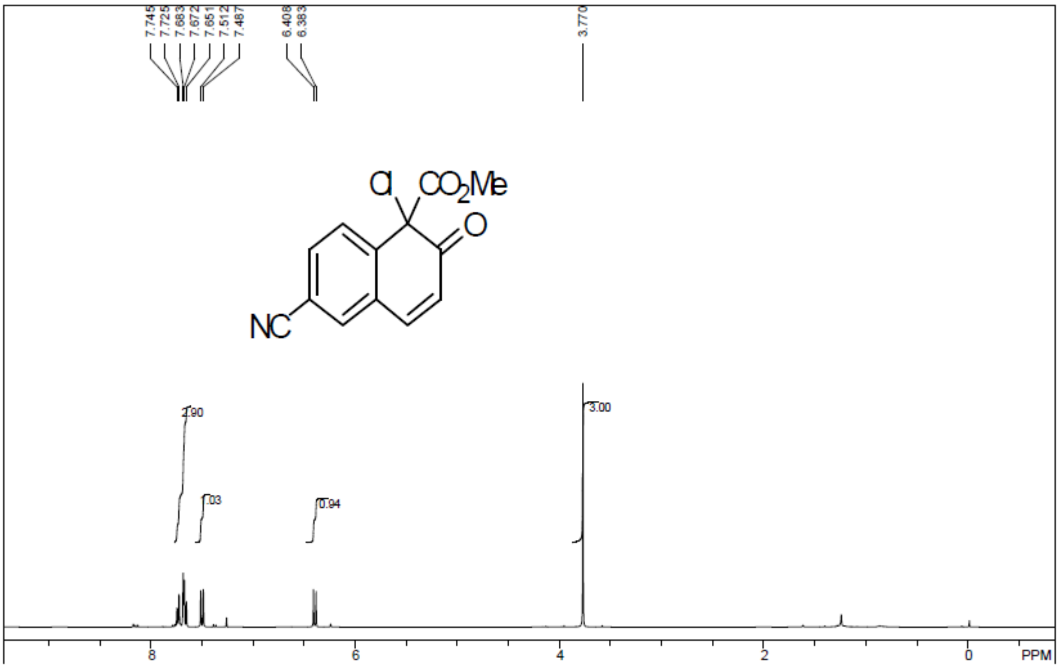

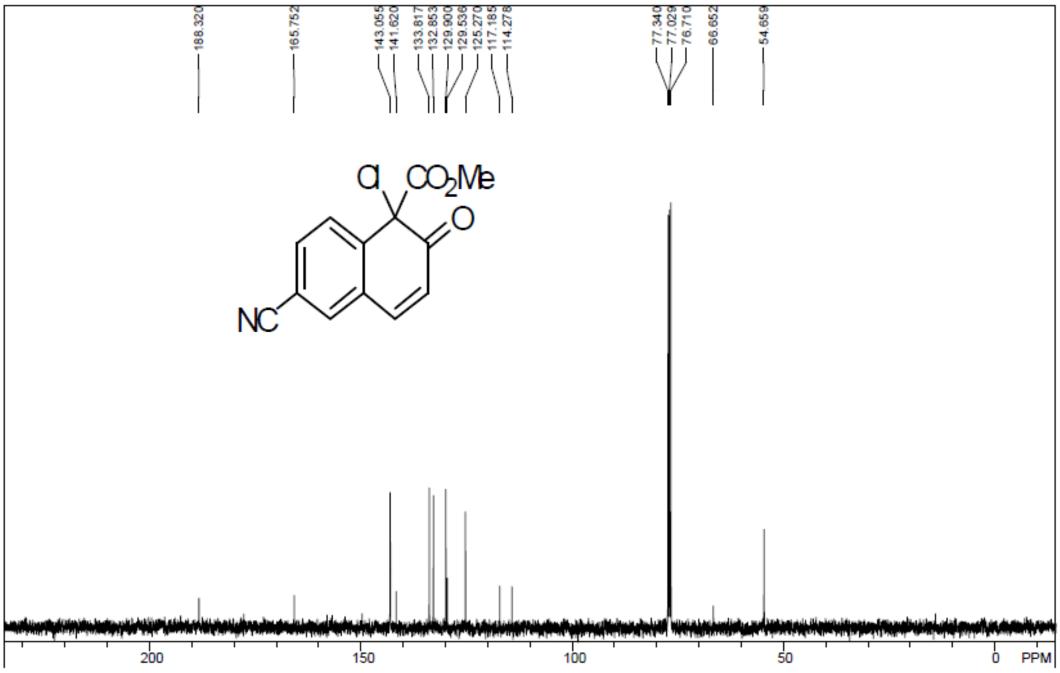

Compound **2f**'s NMR Spectra

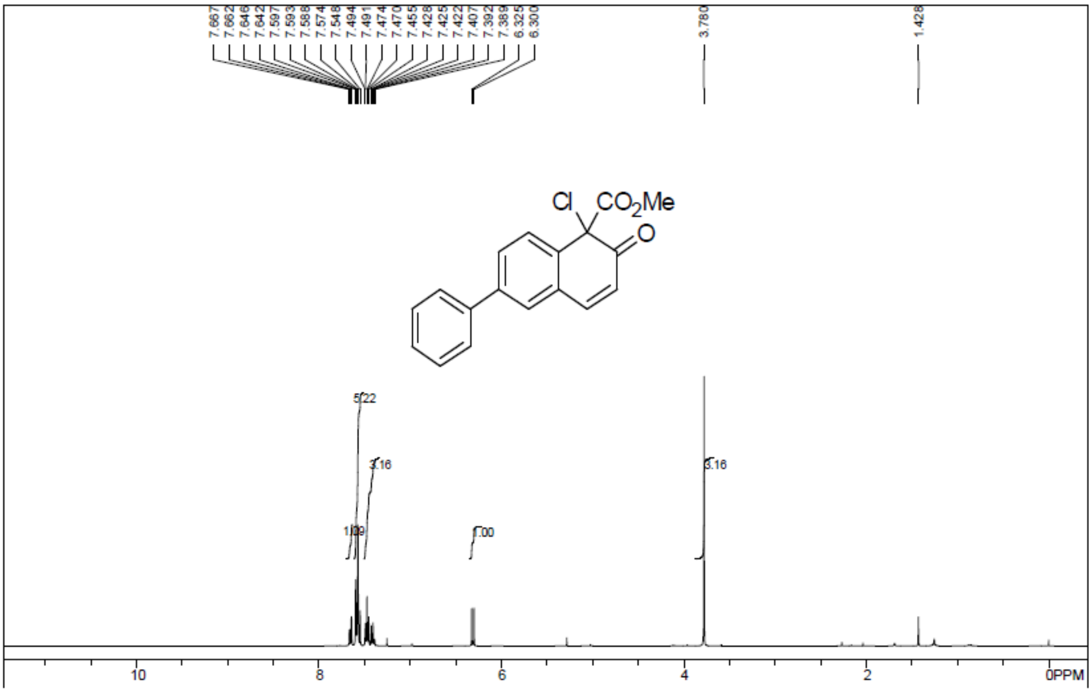

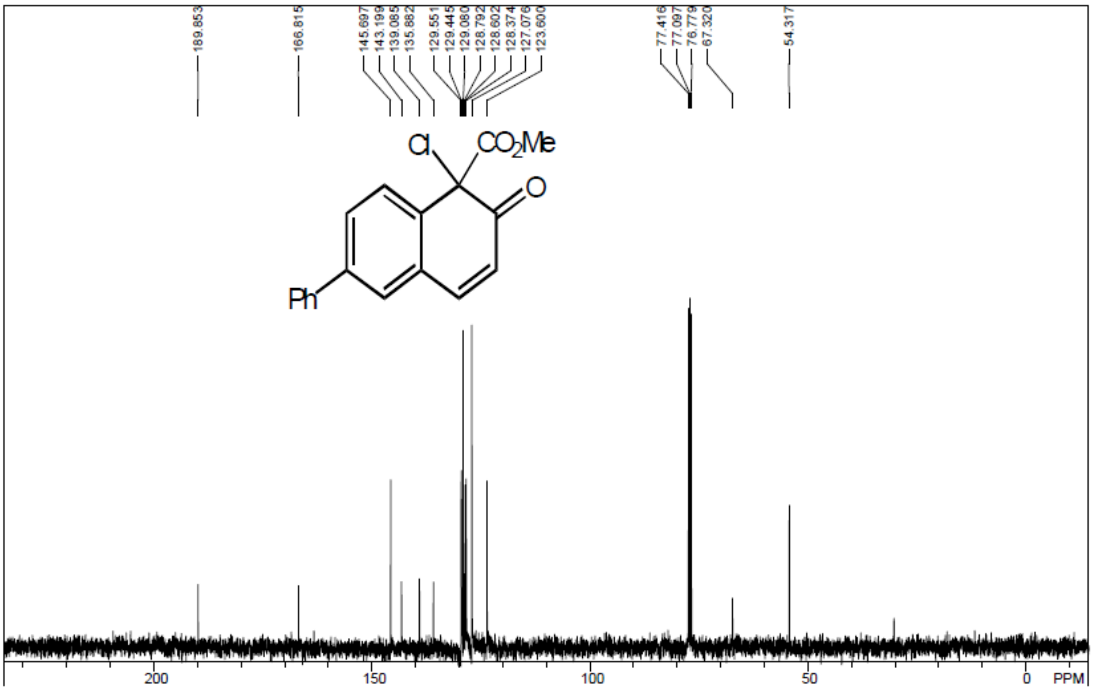

Compound **2g**'s NMR Spectra

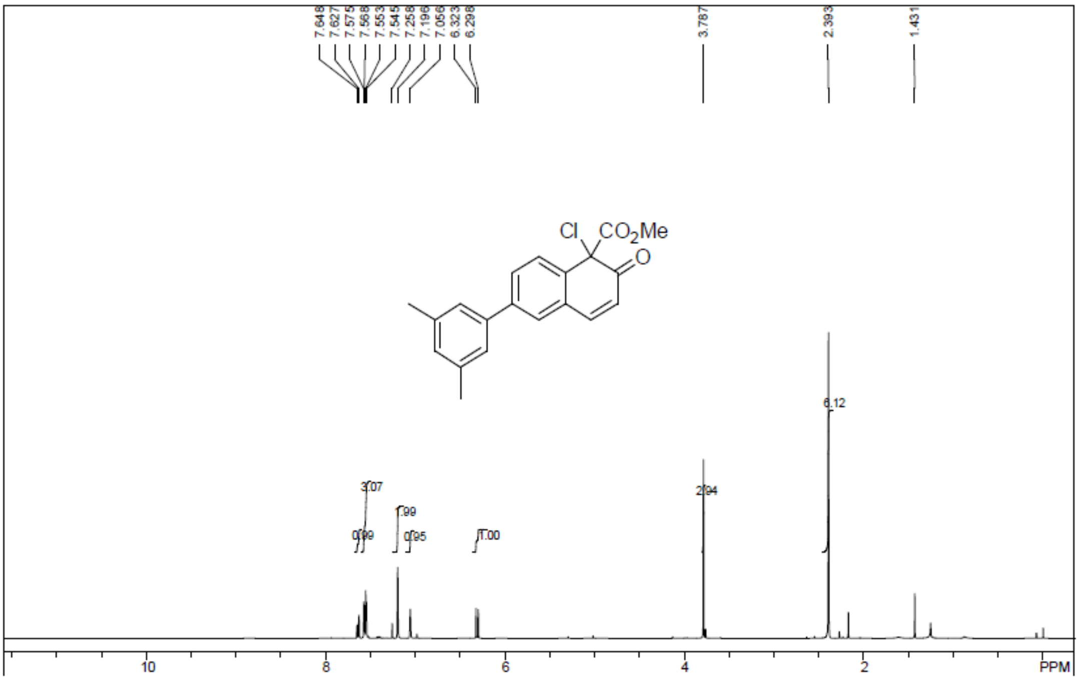

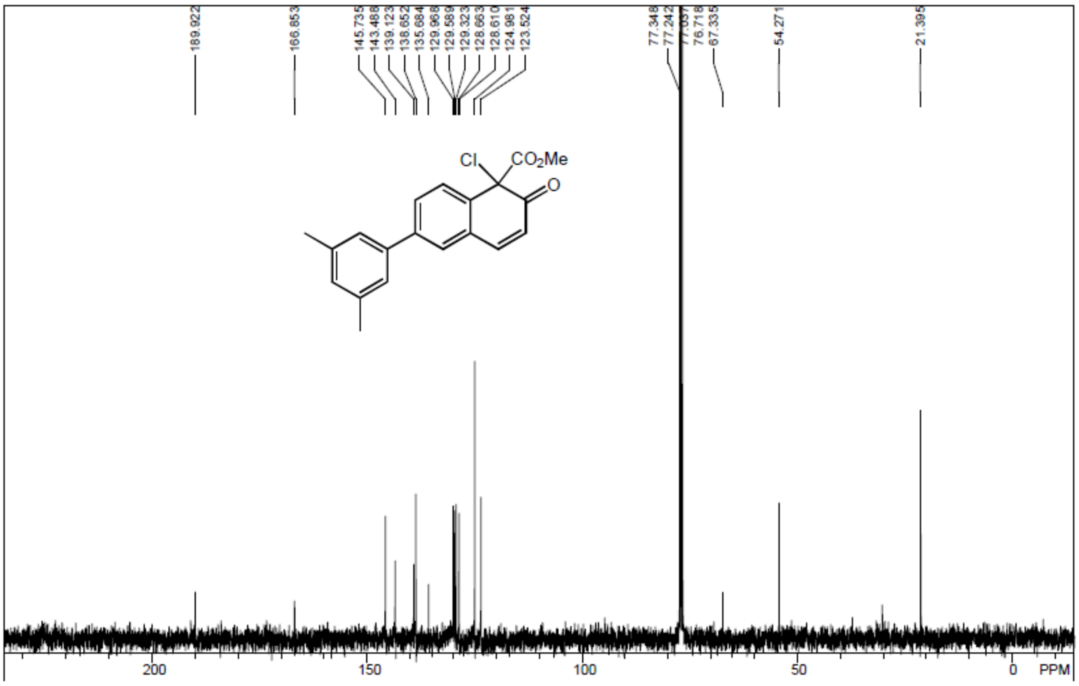

Compound **2h**'s NMR Spectra

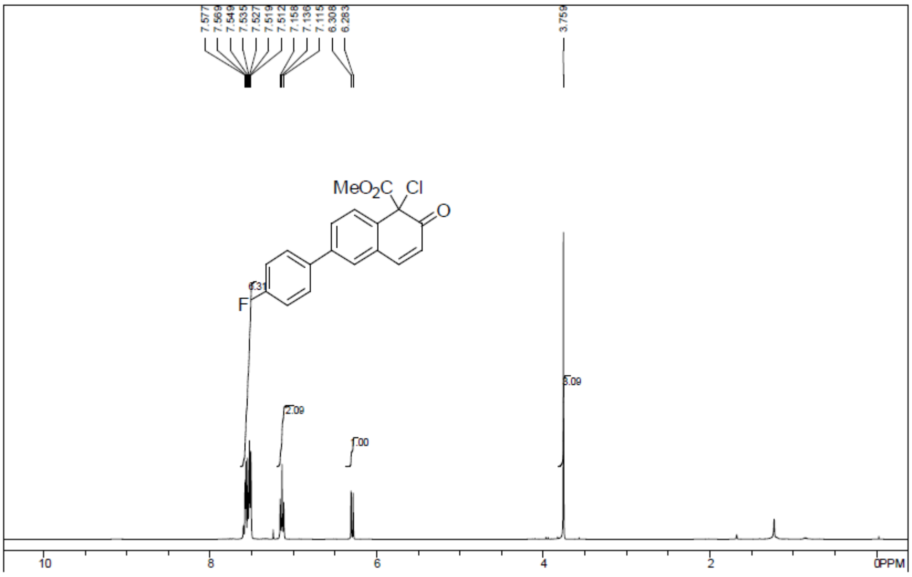

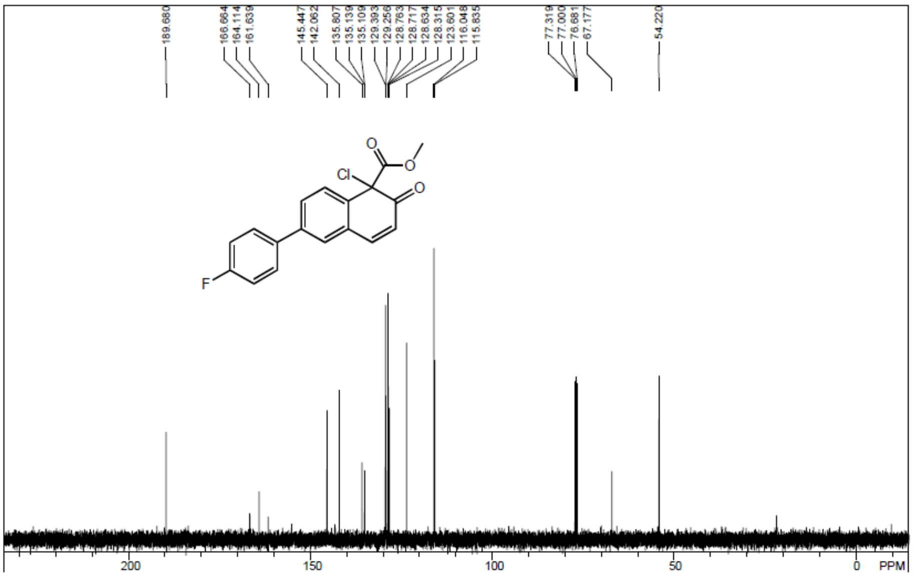

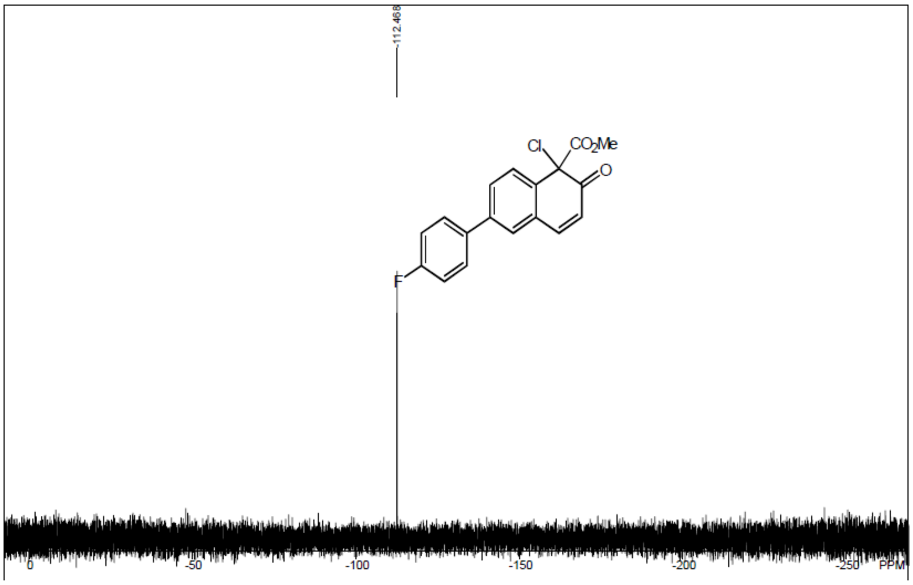

Compound **2i**'s NMR Spectra

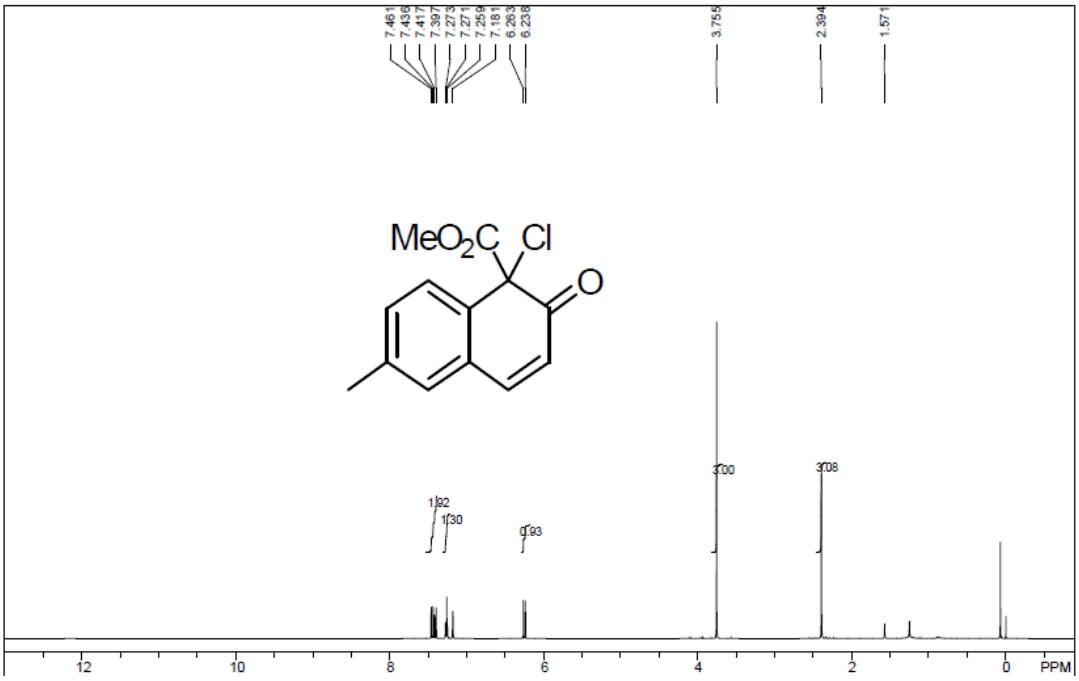

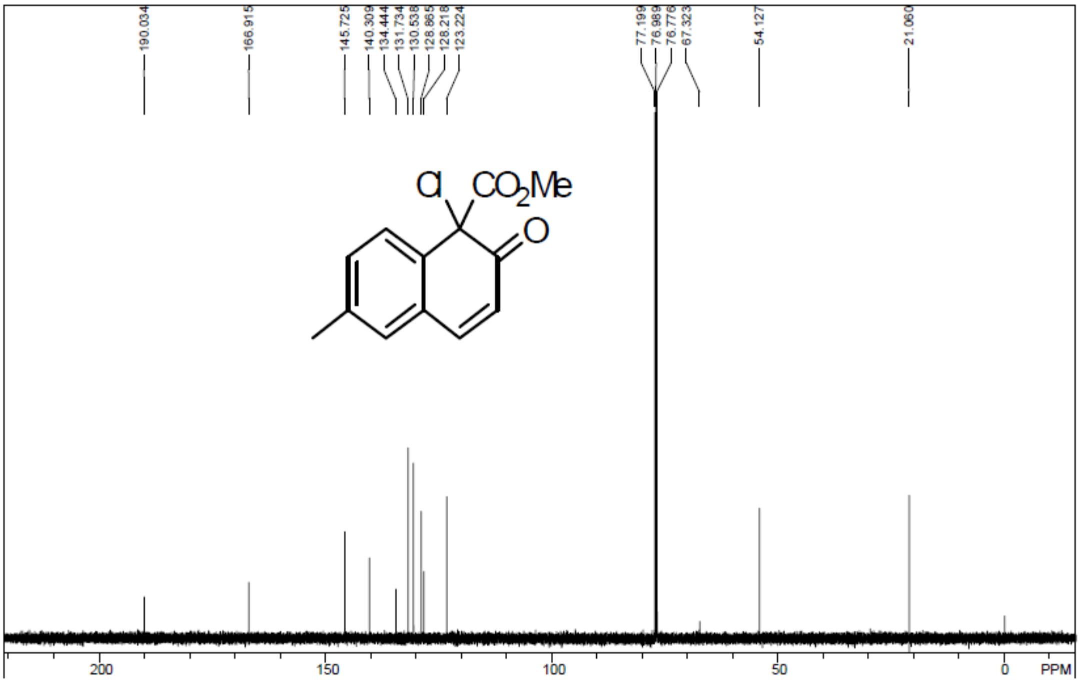

Compound **2j**’s NMR Spectra

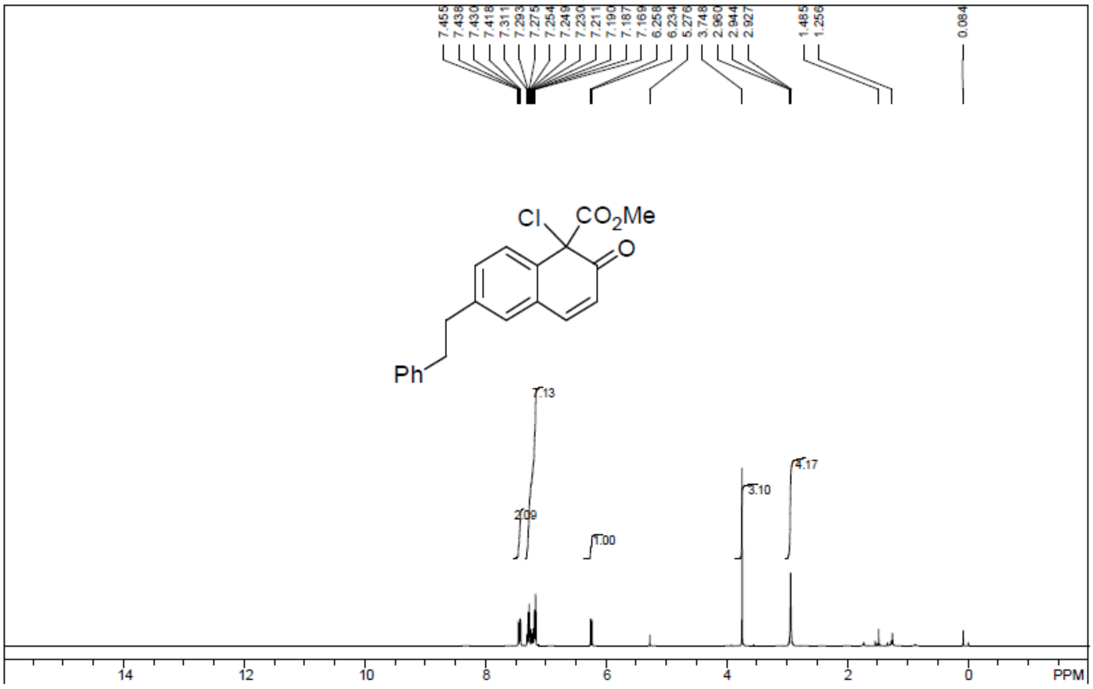

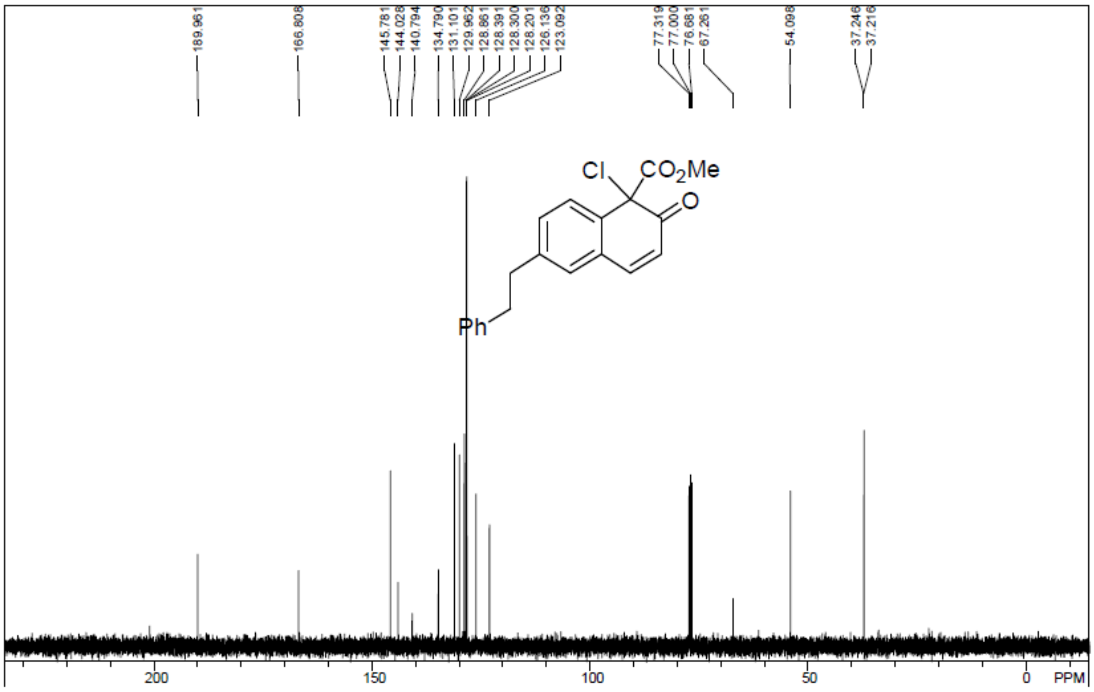

Compound **2k**'s NMR Spectra

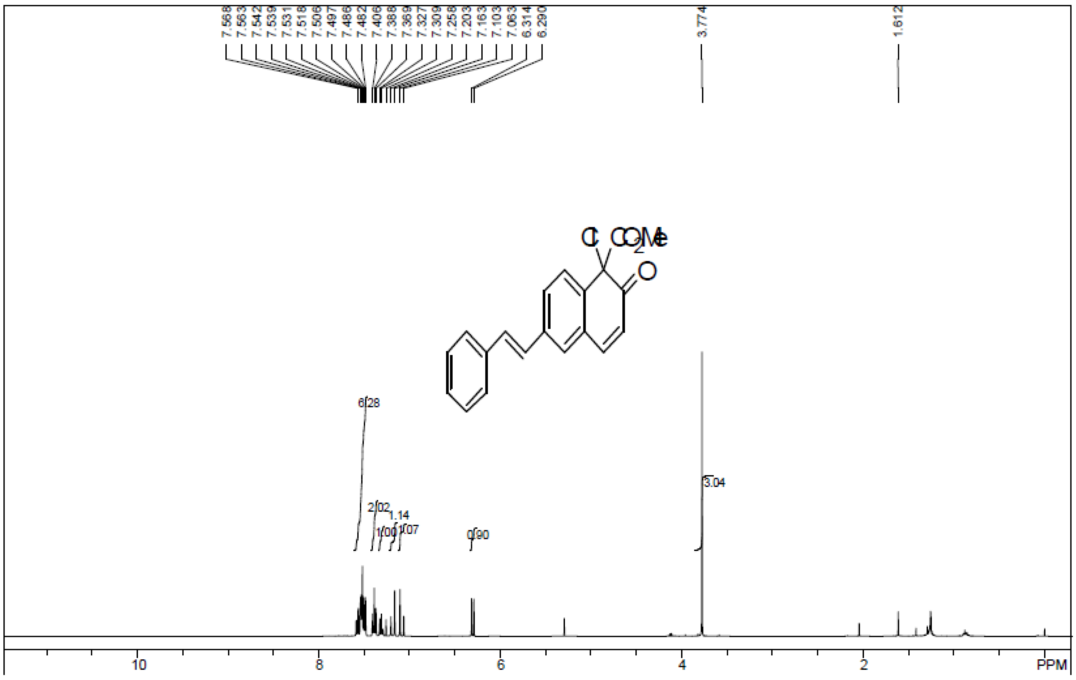

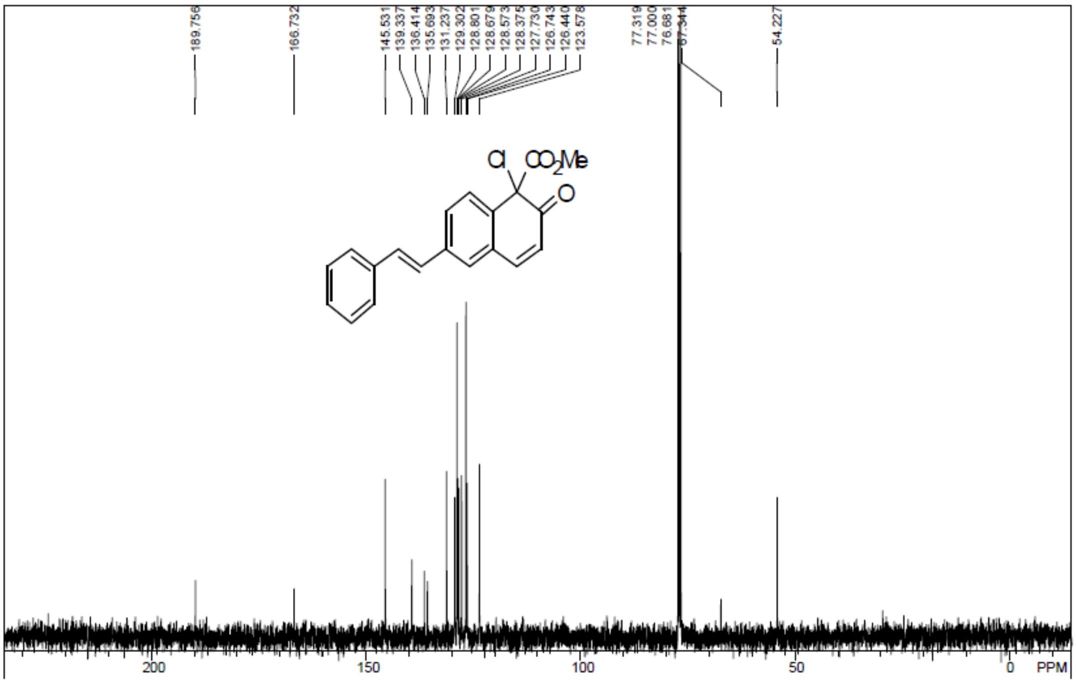

Compound **2l**'s NMR Spectra

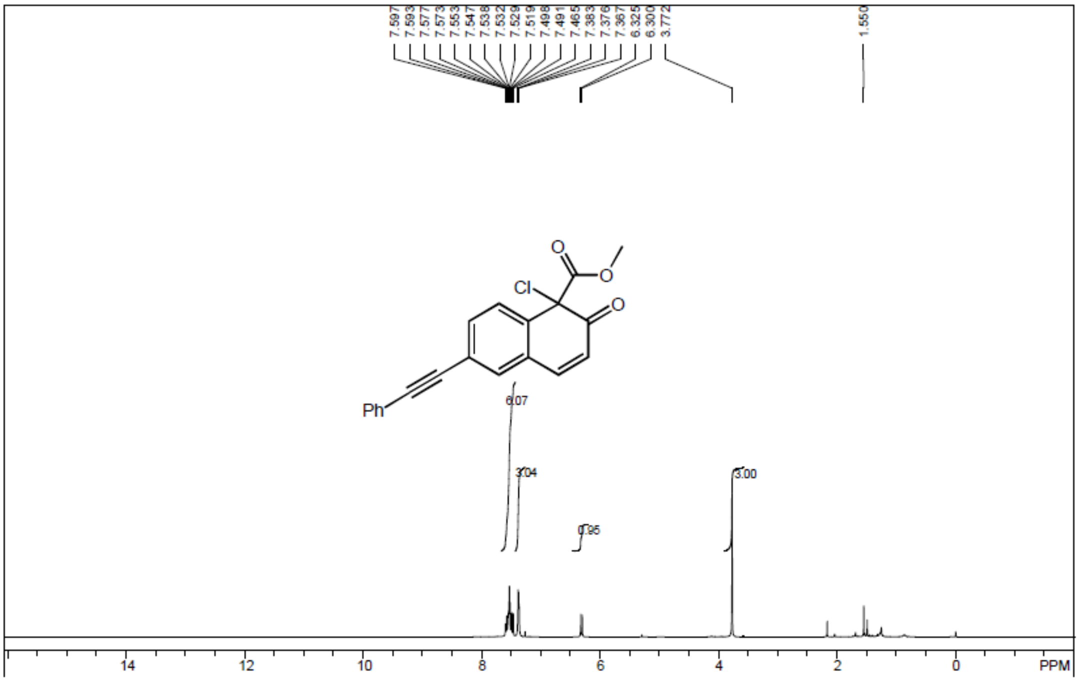

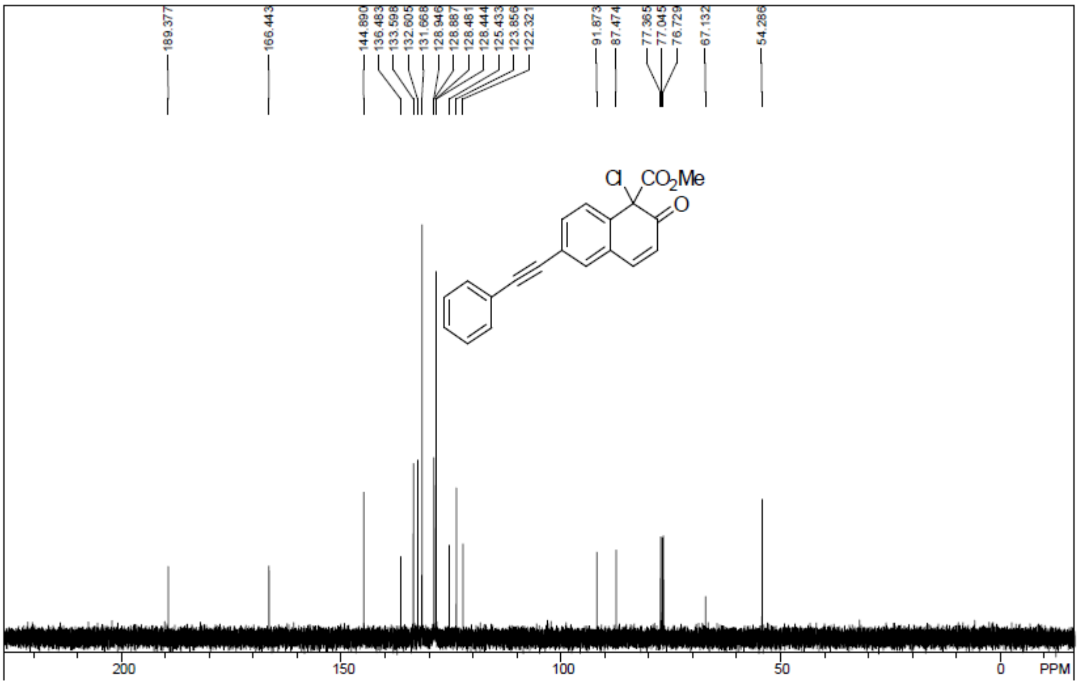

Compound **2m**'s NMR Spectra

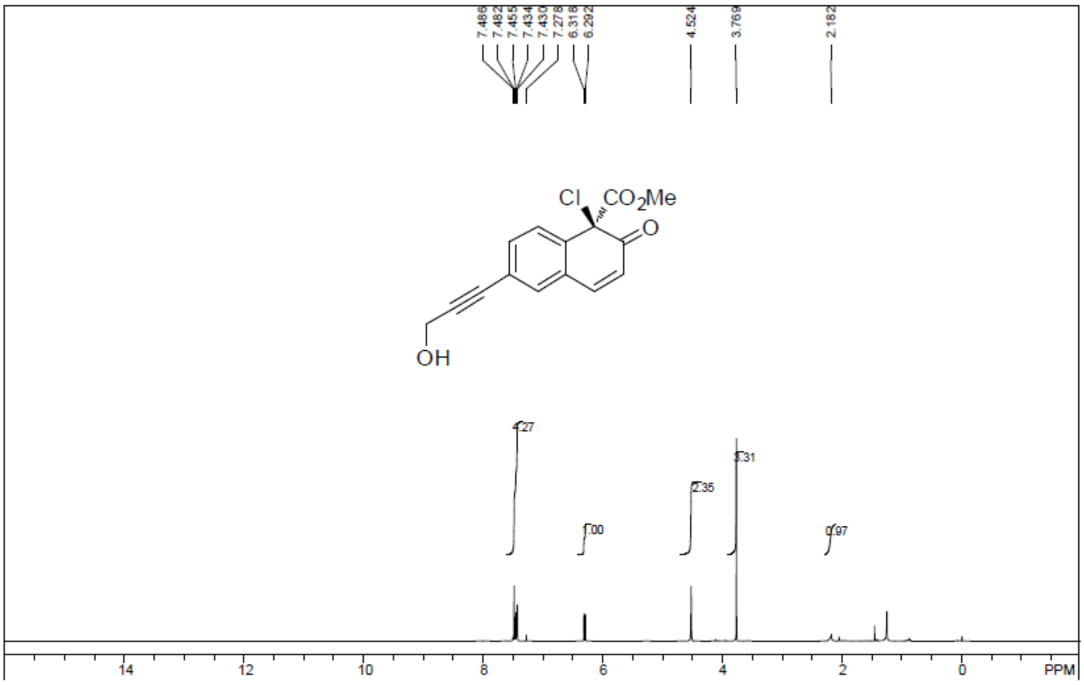

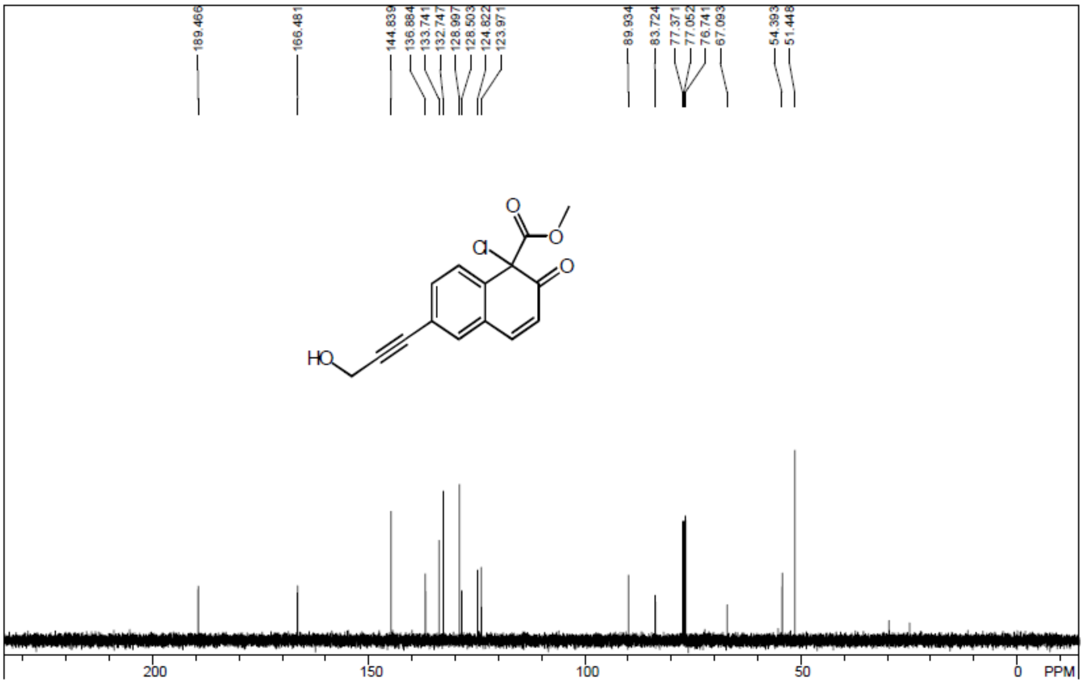

Compound **2n**'s NMR Spectra

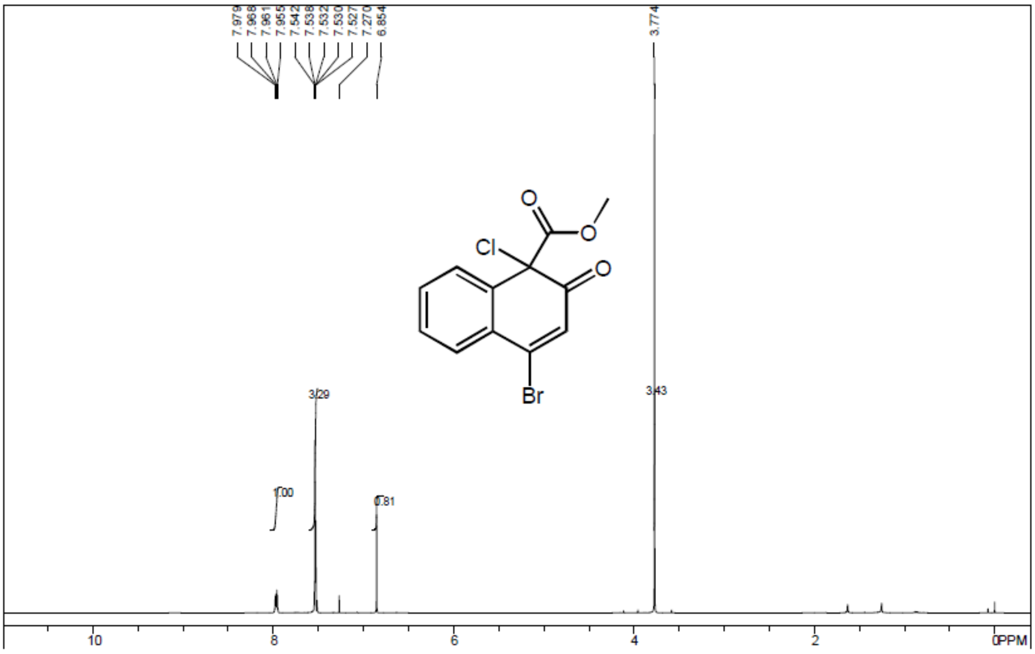

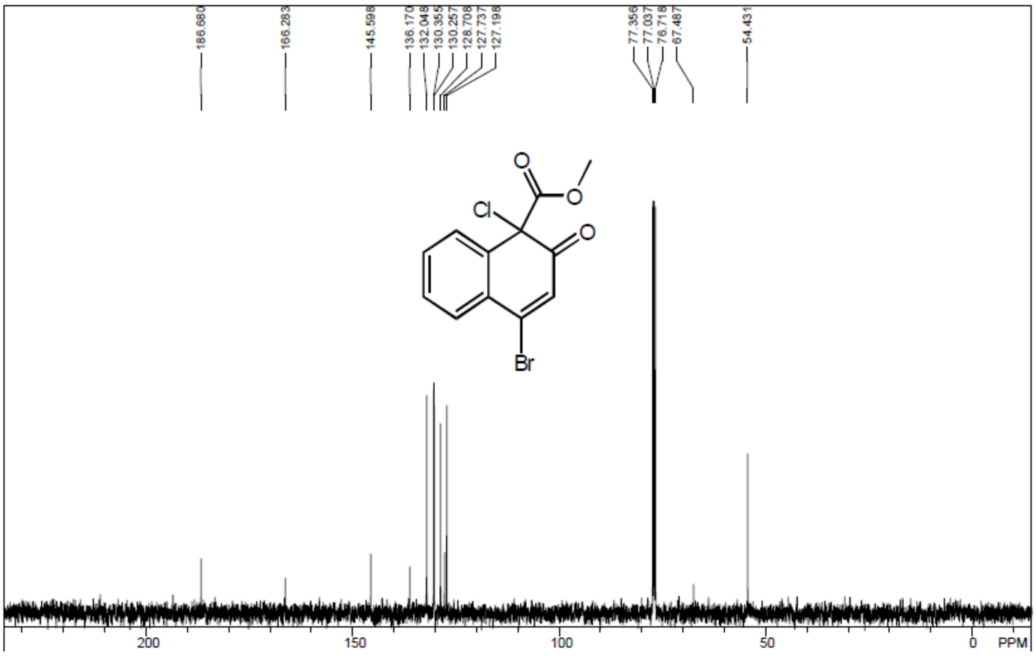

Compound **2o**'s NMR Spectra

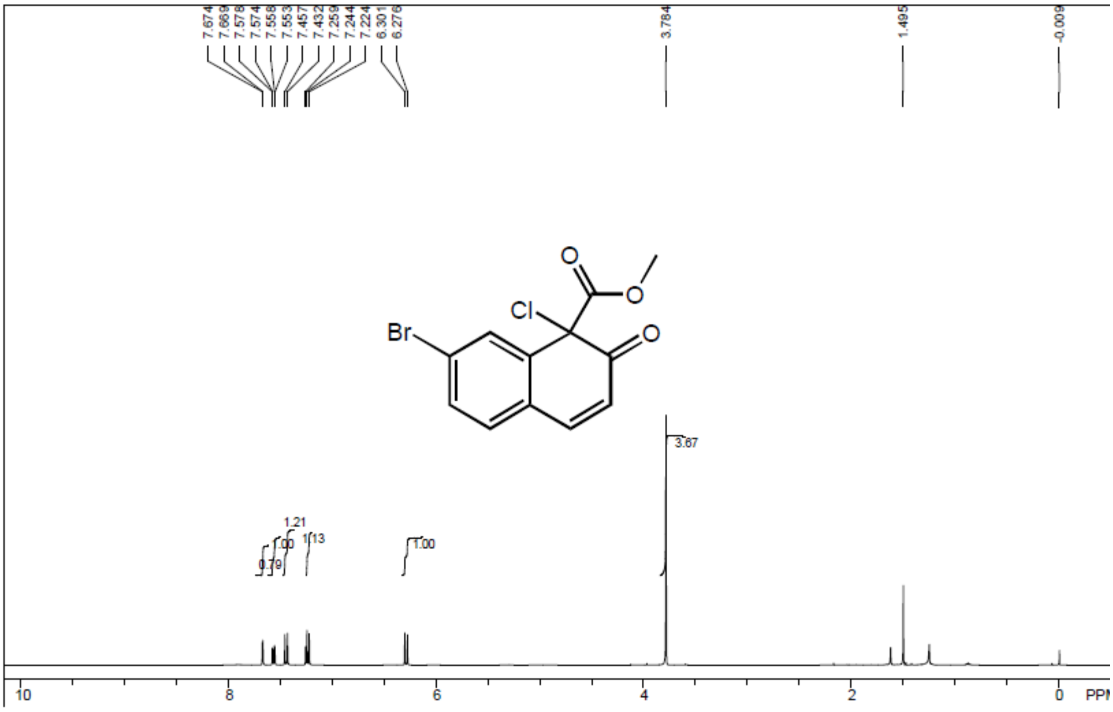

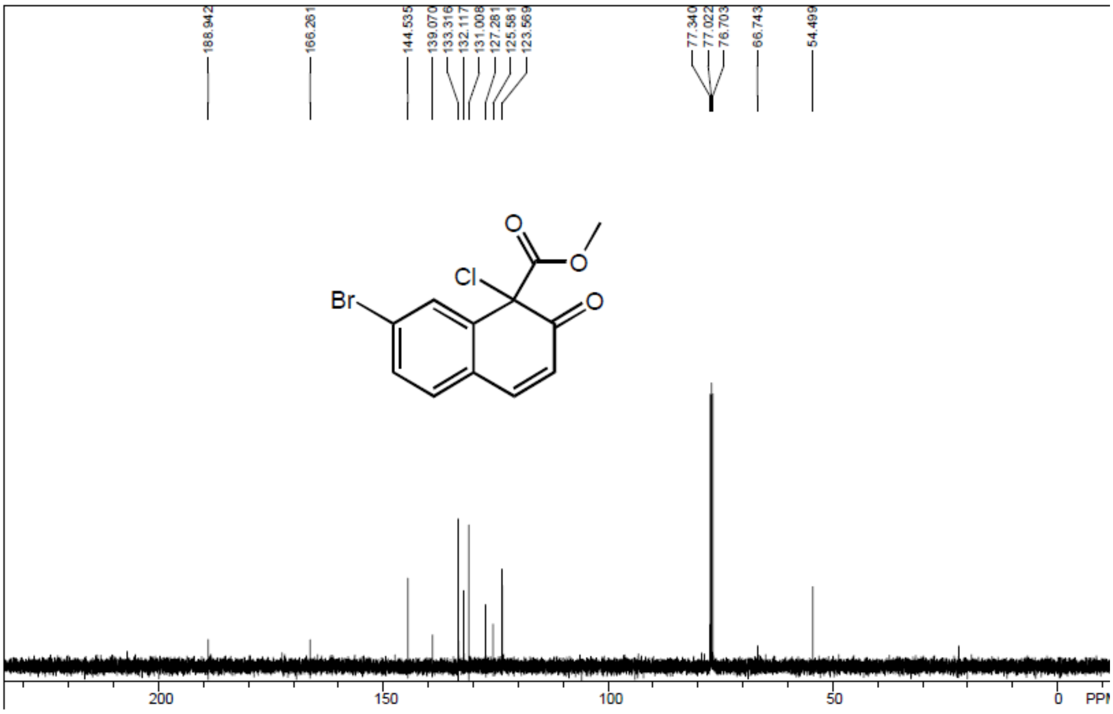

Compound **2p**’s NMR Spectra

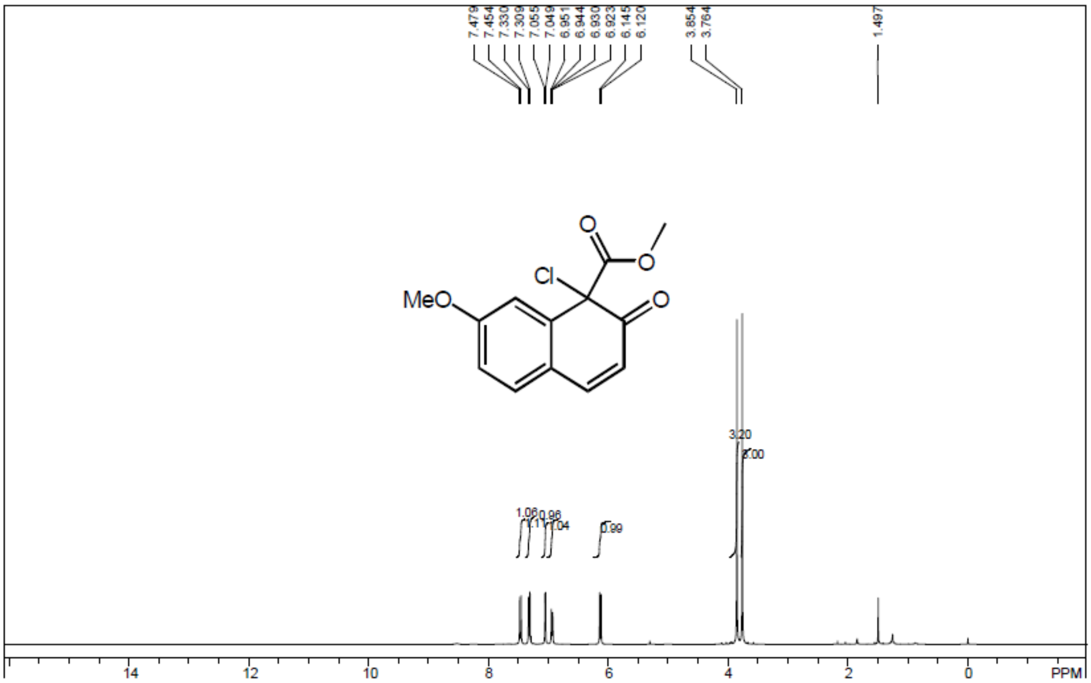

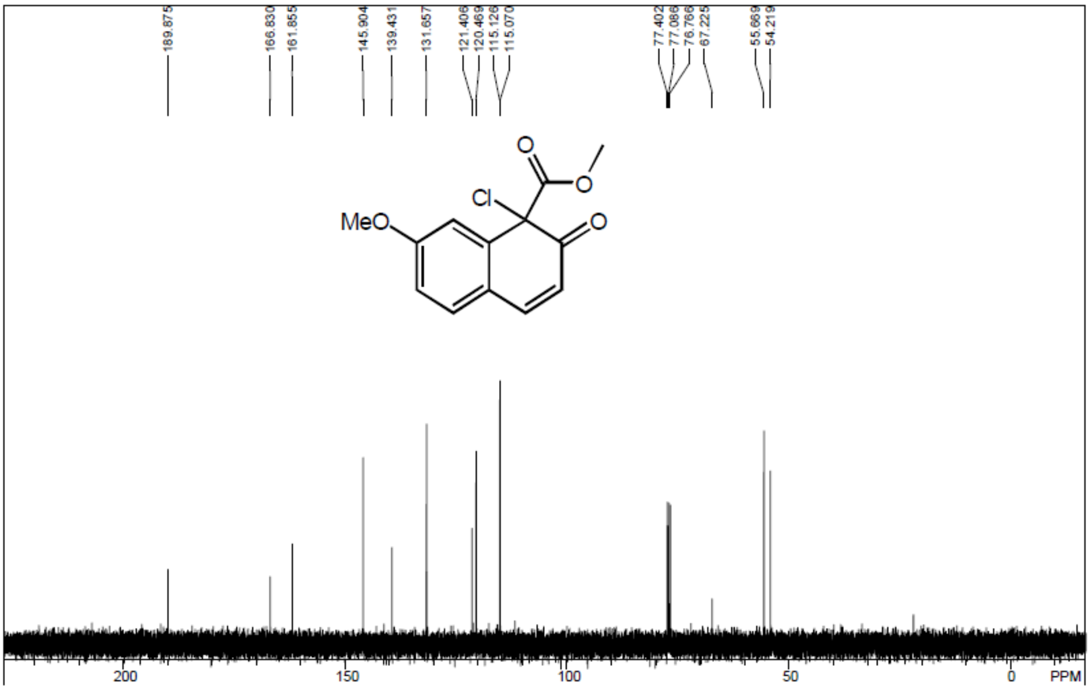

Compound **2q**'s NMR Spectra

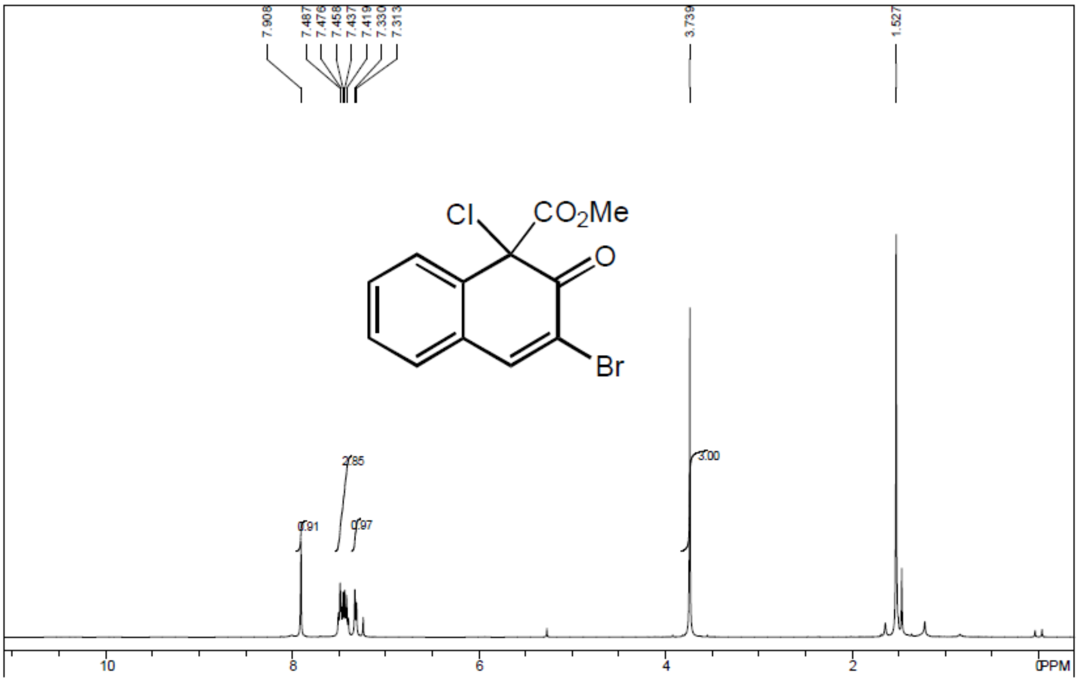

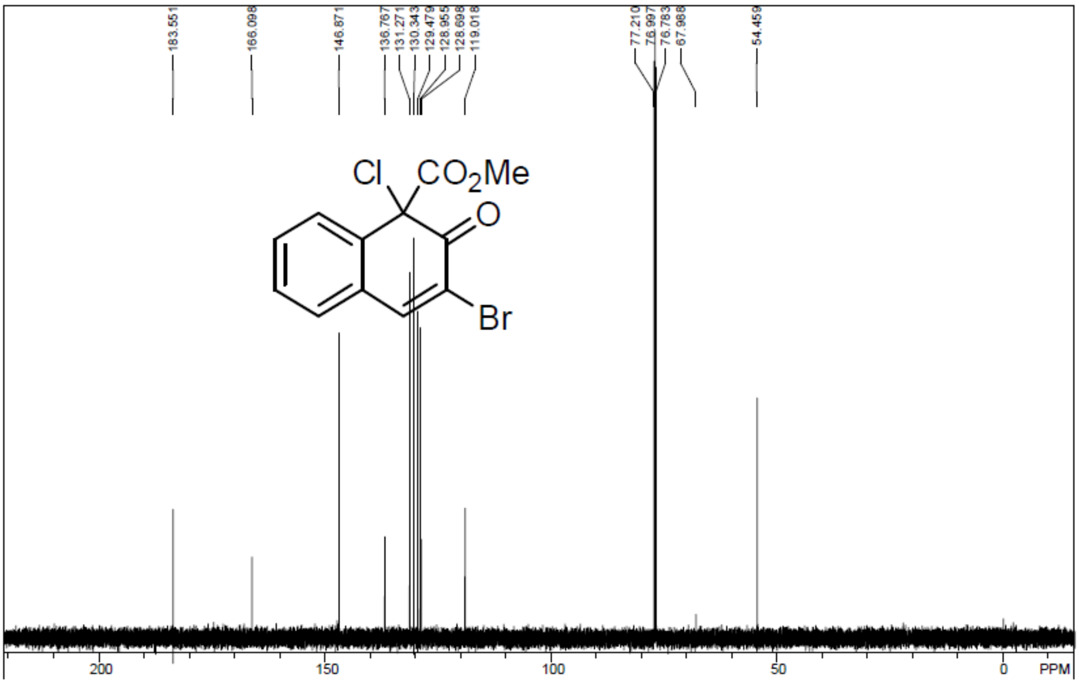

Compound **2r**’s NMR Spectra

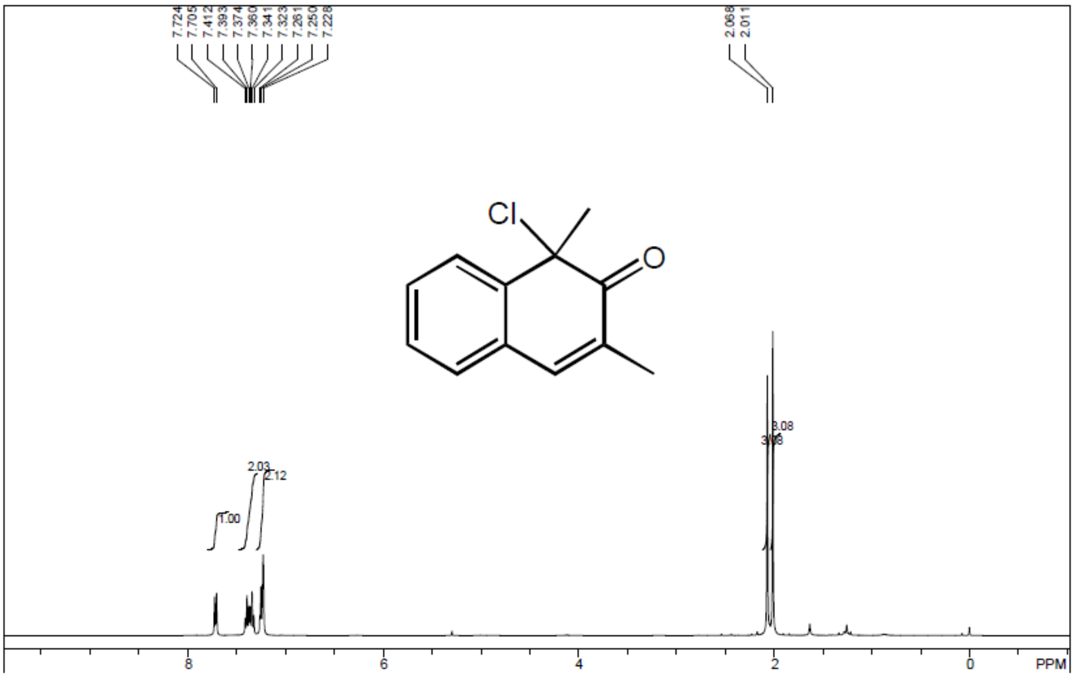

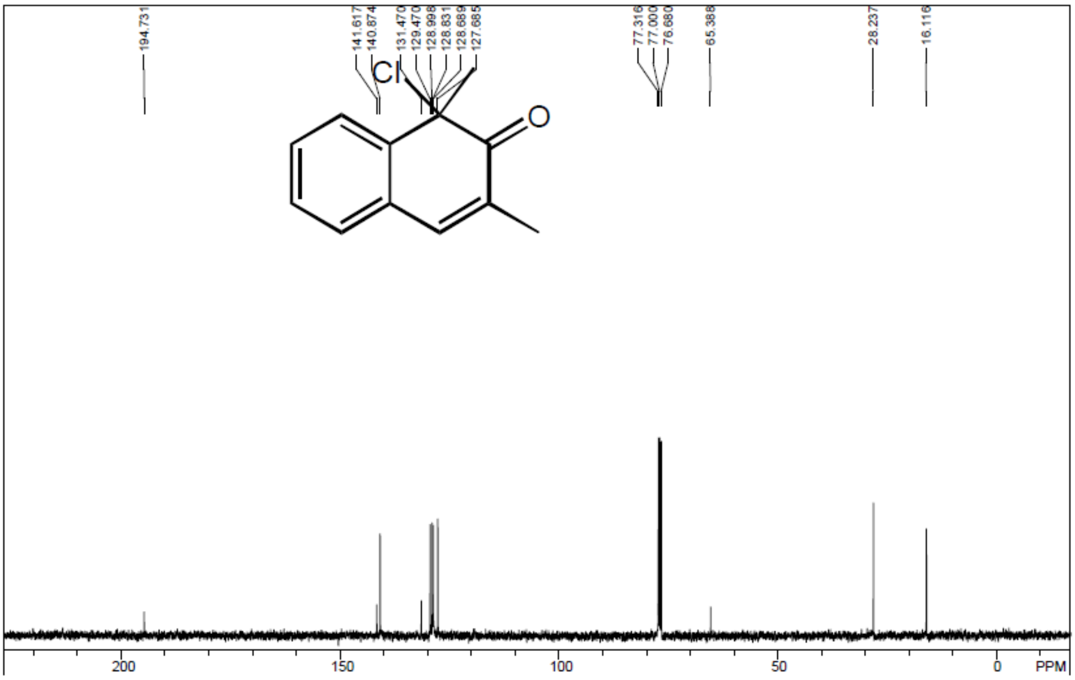

Compound **2s**'s NMR Spectra

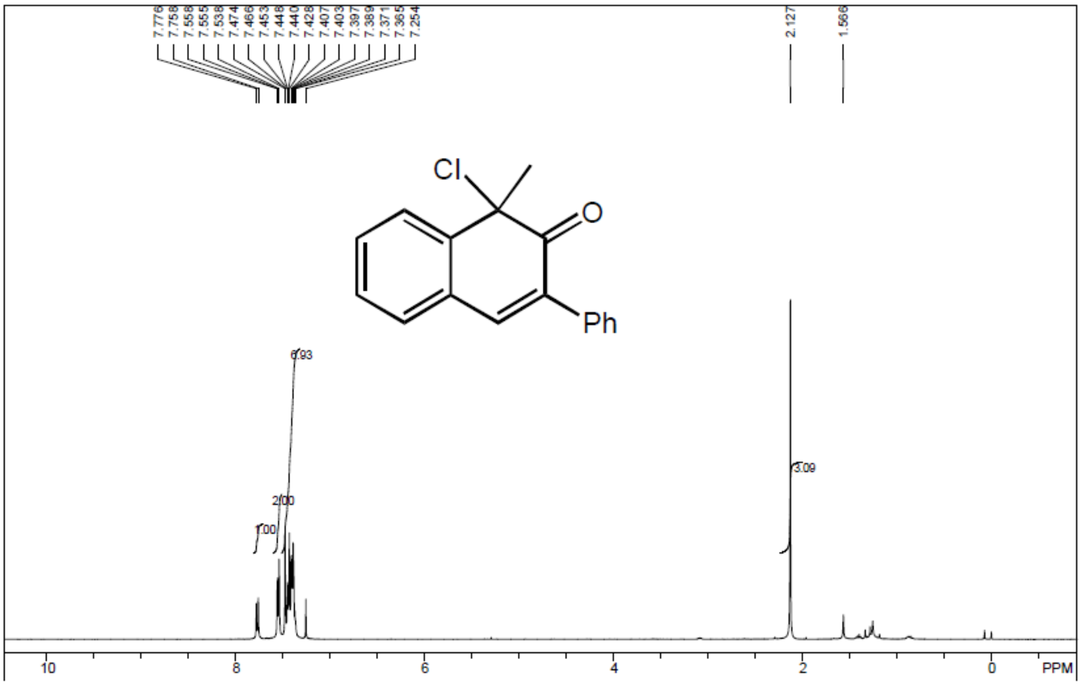

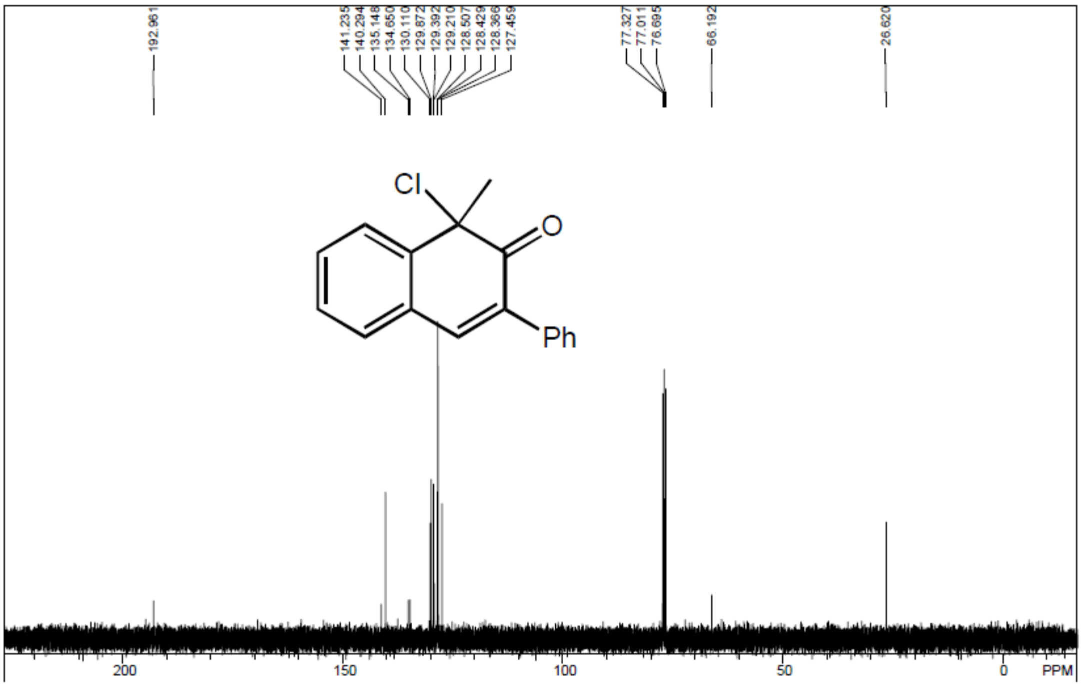

Compound **2t**'s NMR Spectra

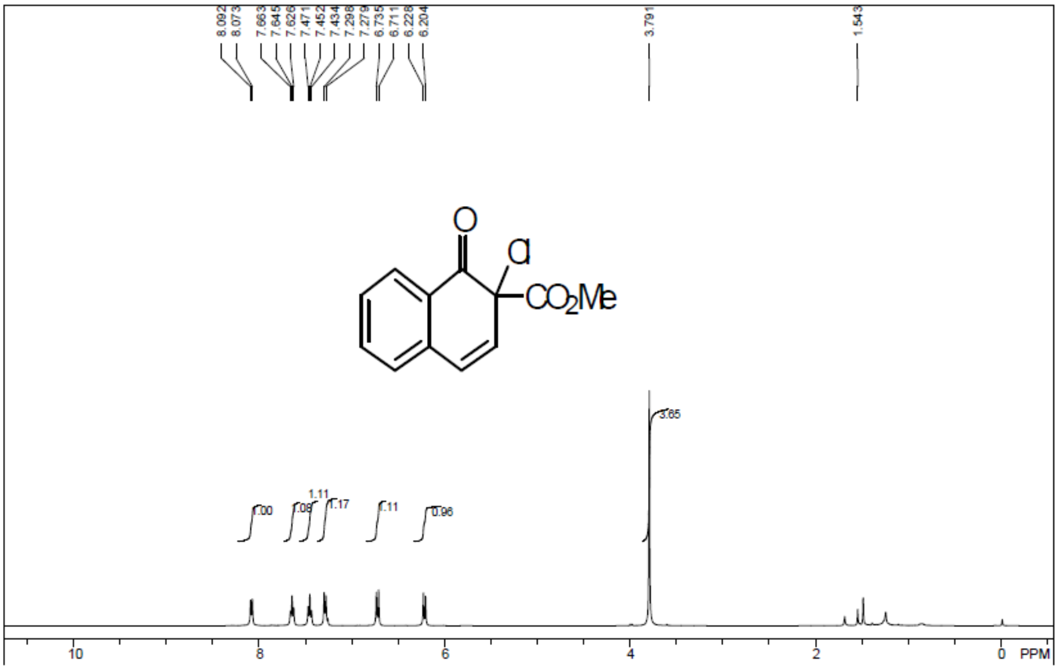

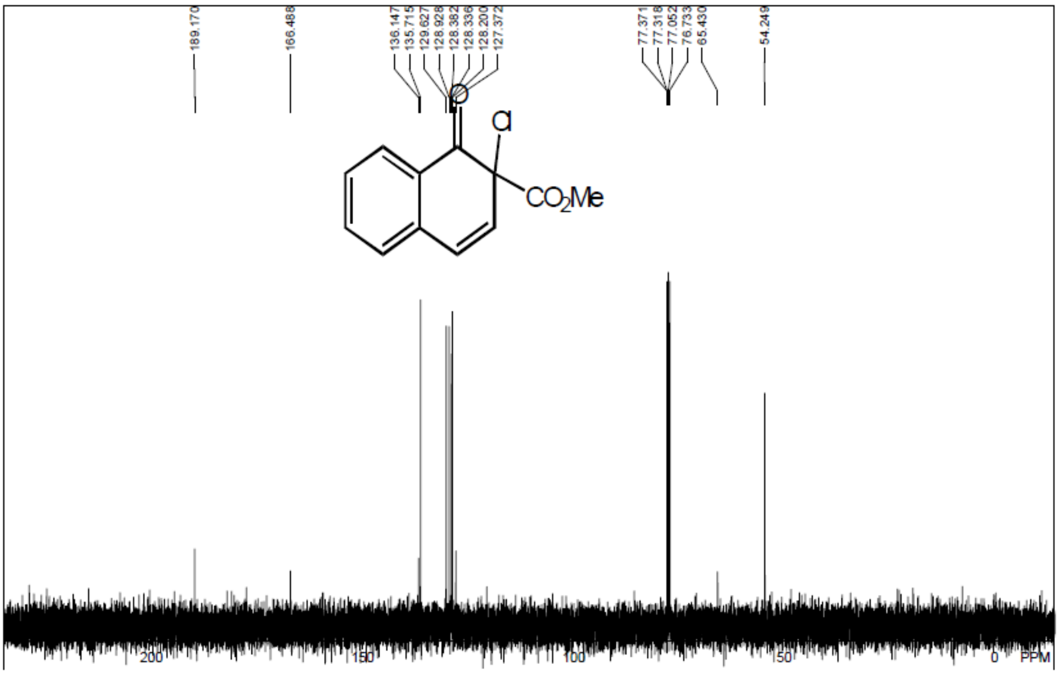

Compound **2u**'s NMR Spectra

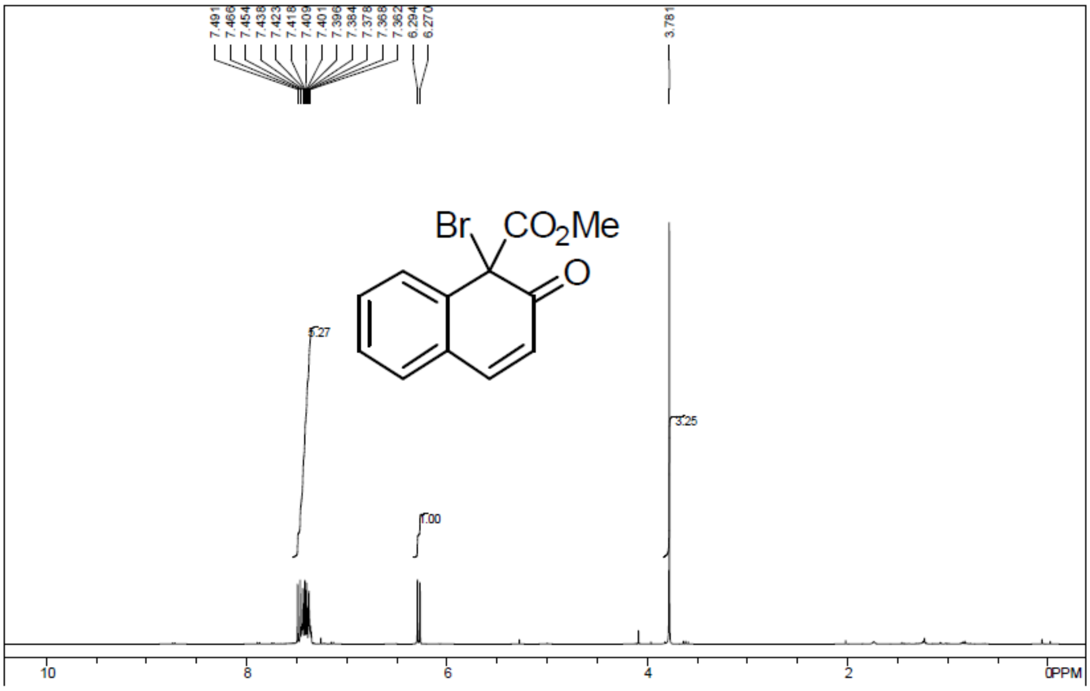

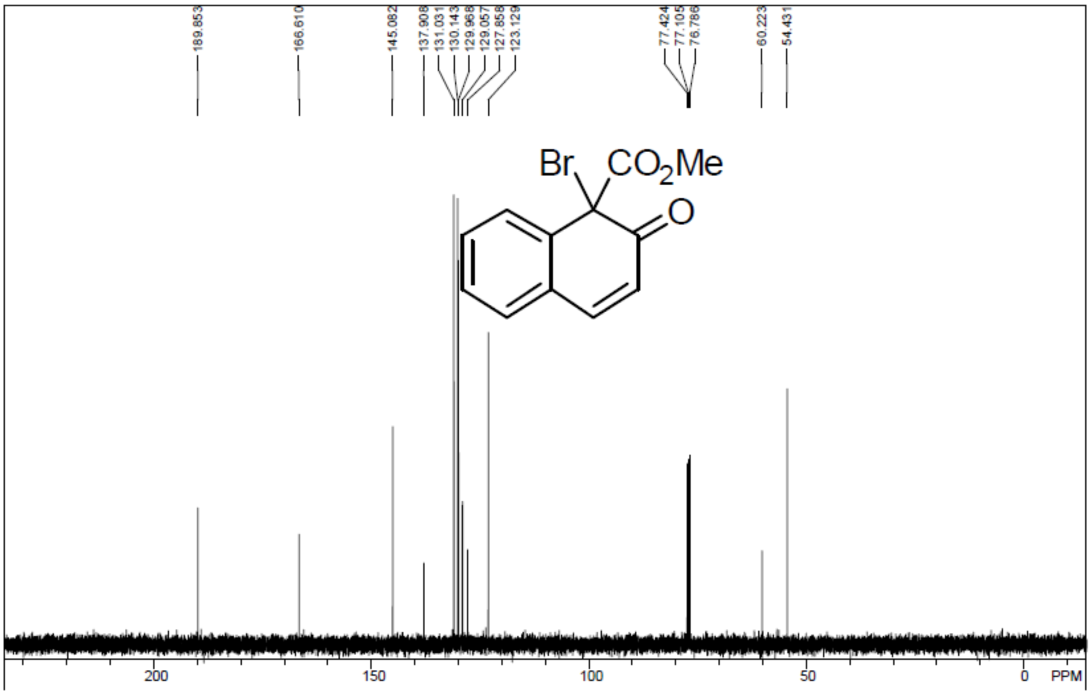

Compound **2v**'s NMR Spectra

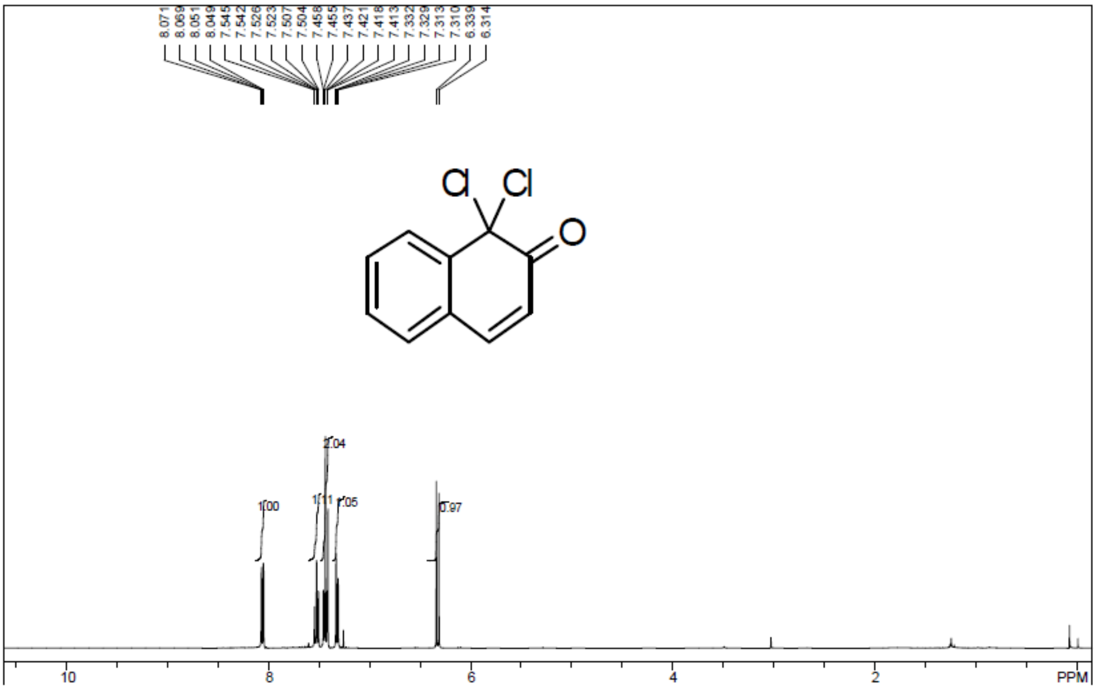

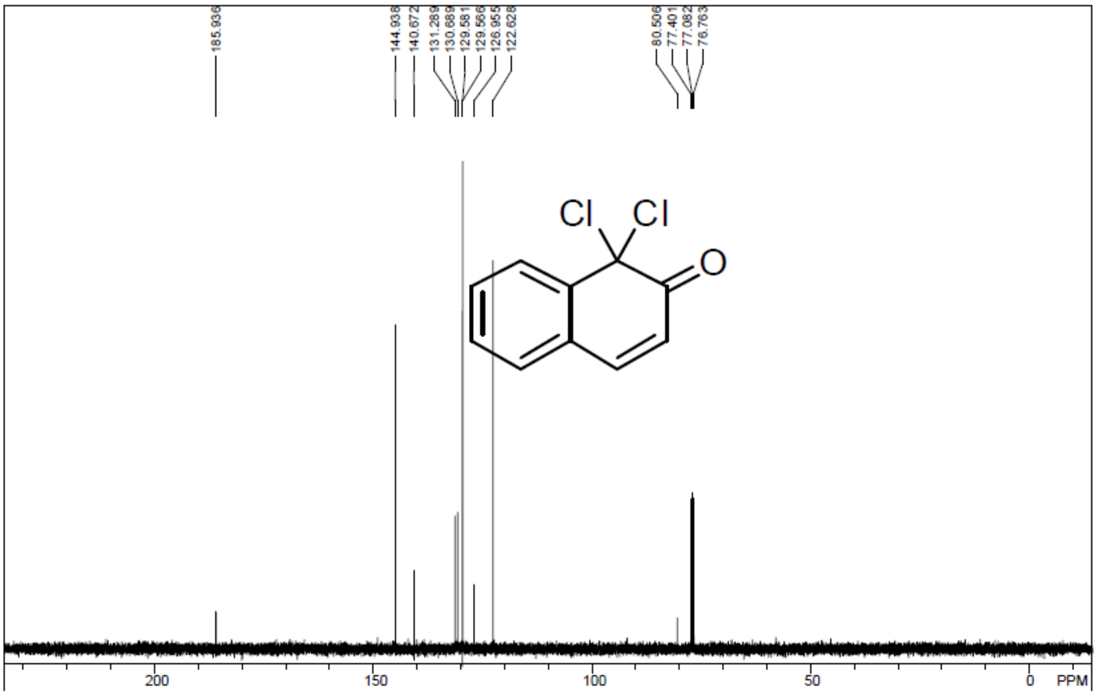

Compound **3a**'s NMR Spectra

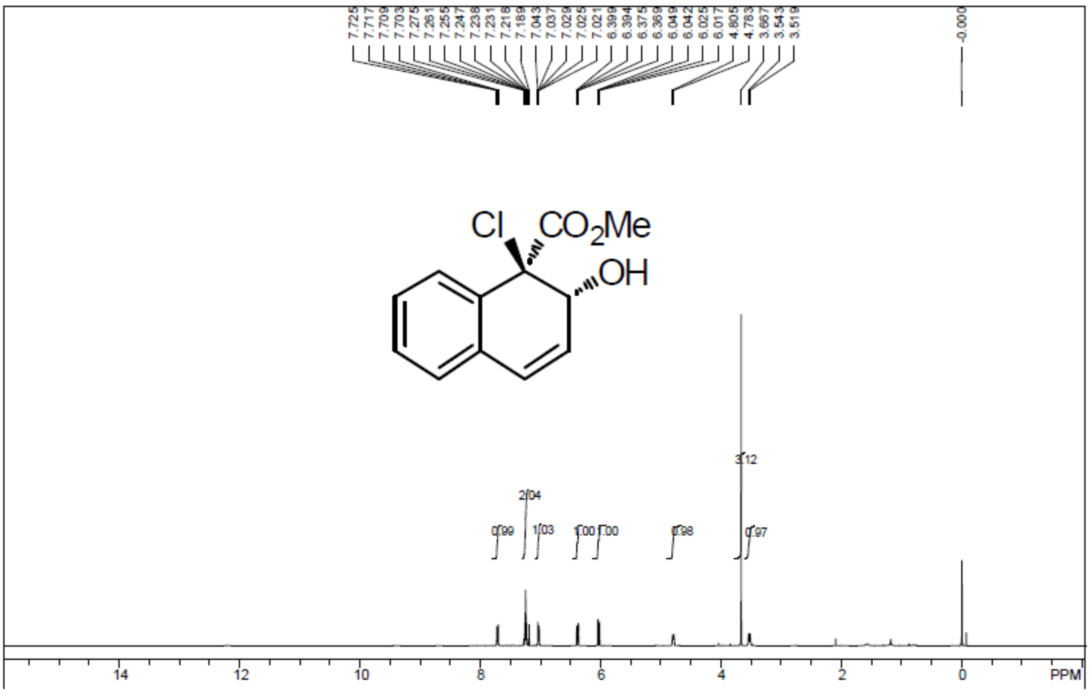

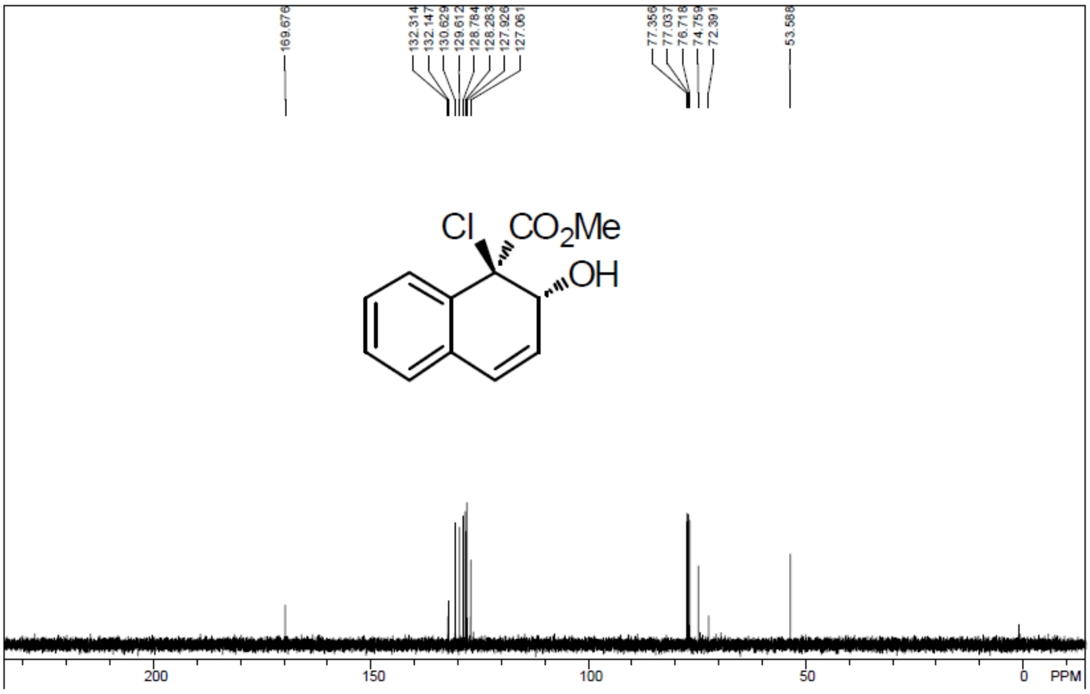

Compound **3b**'s NMR Spectra

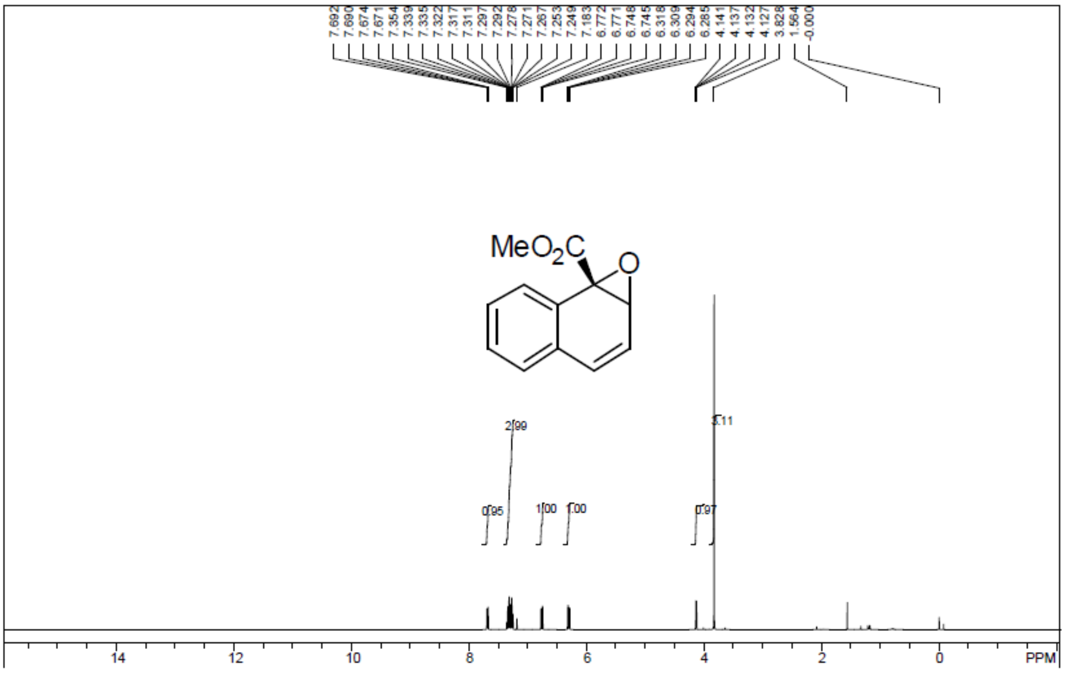

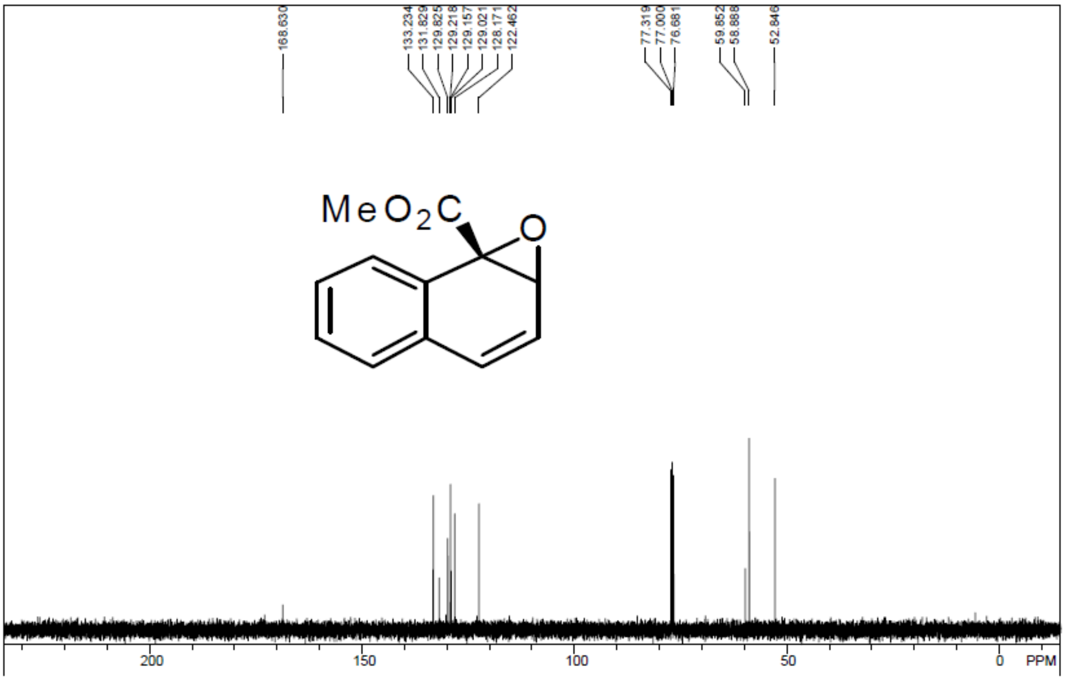

Compound **3c**'s NMR Spectra

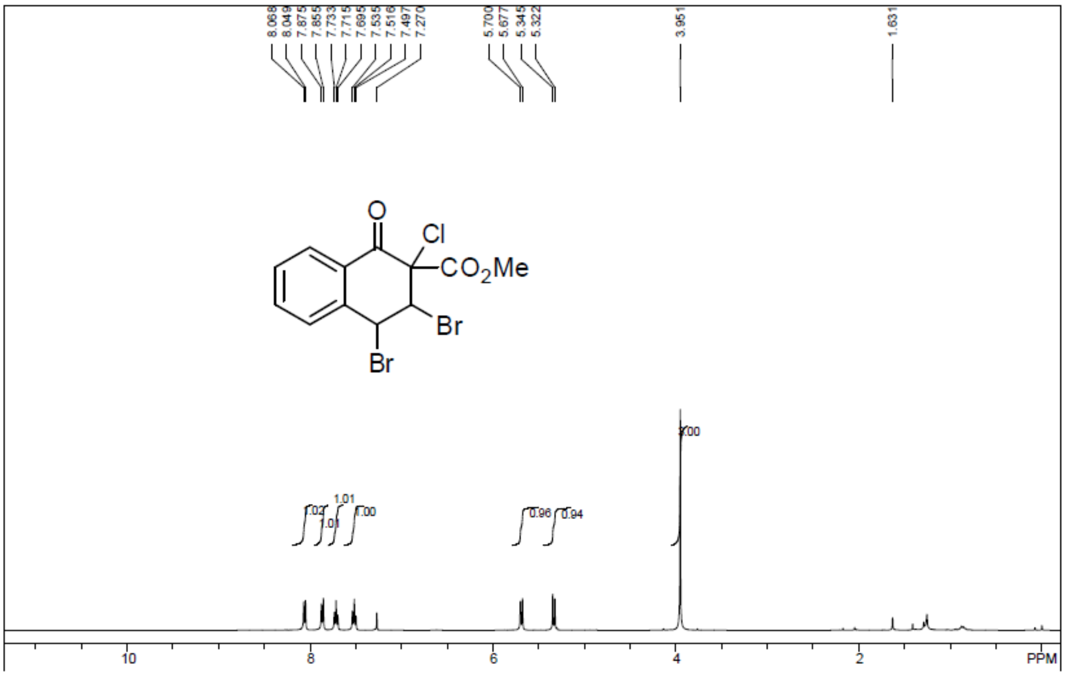

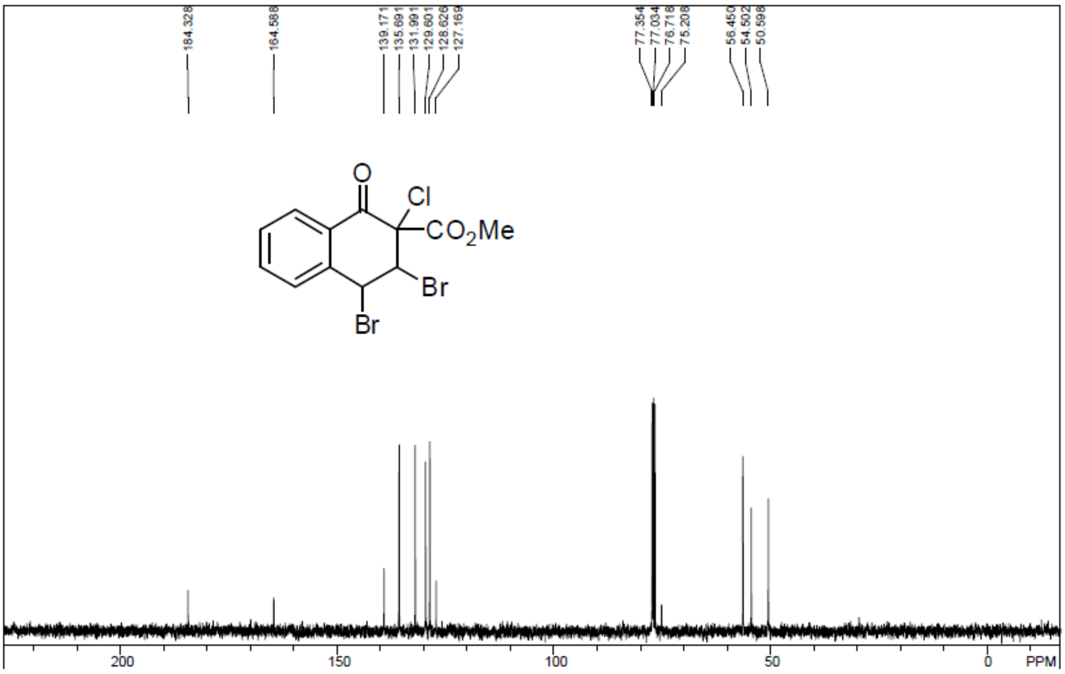

Sample Name:

Archive directory:
/export/home/omc/vnmrsys/data
Sample directory:
yq-21-98-1_03Jun2014
FidFile: NOESY

Pulse Sequence: NOESY
Solvent: CDCl3
Data collected on: Jun 3 2014

Operator: omc
VNMRS-600 "OMC-NMR600"

Relax. delay 2.000 sec
Mixing 0.800 sec
Acq. time 0.142 sec
Width 3597.1 Hz
2D Width 3597.1 Hz
32 repetitions
2 x 128 increments
OBSERVE H1, 300.0268138 MHz
DATA PROCESSING
Line broadening 3.0 Hz
Gauss apodization 0.045 sec
F1 DATA PROCESSING
Gauss apodization 0.033 sec
FT size 2048 x 2048
Total time 4 hr, 56 min

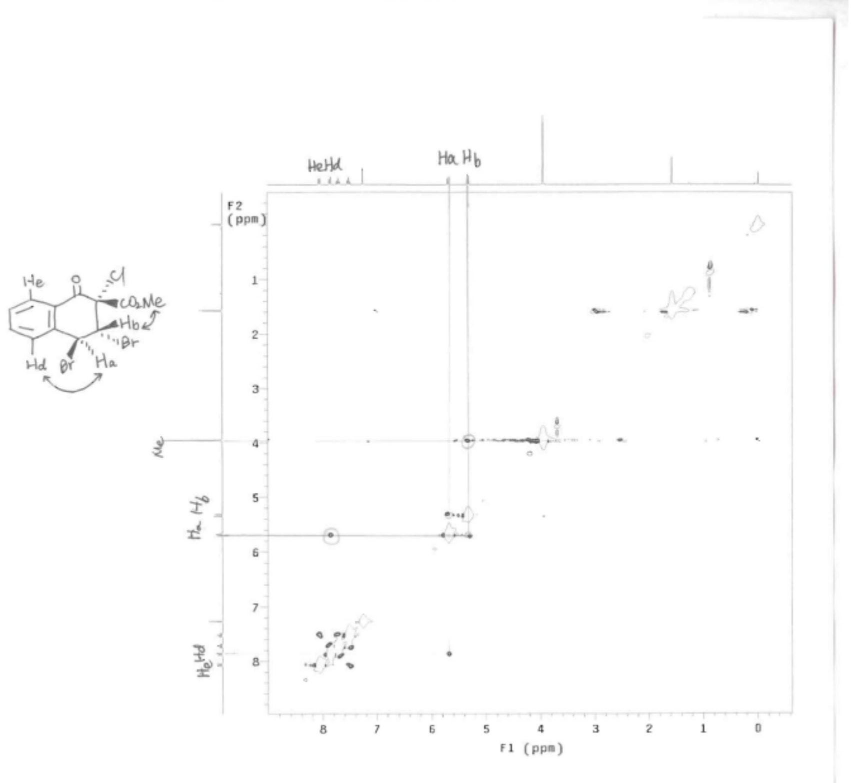

Compound **3d**'s NMR Spectra

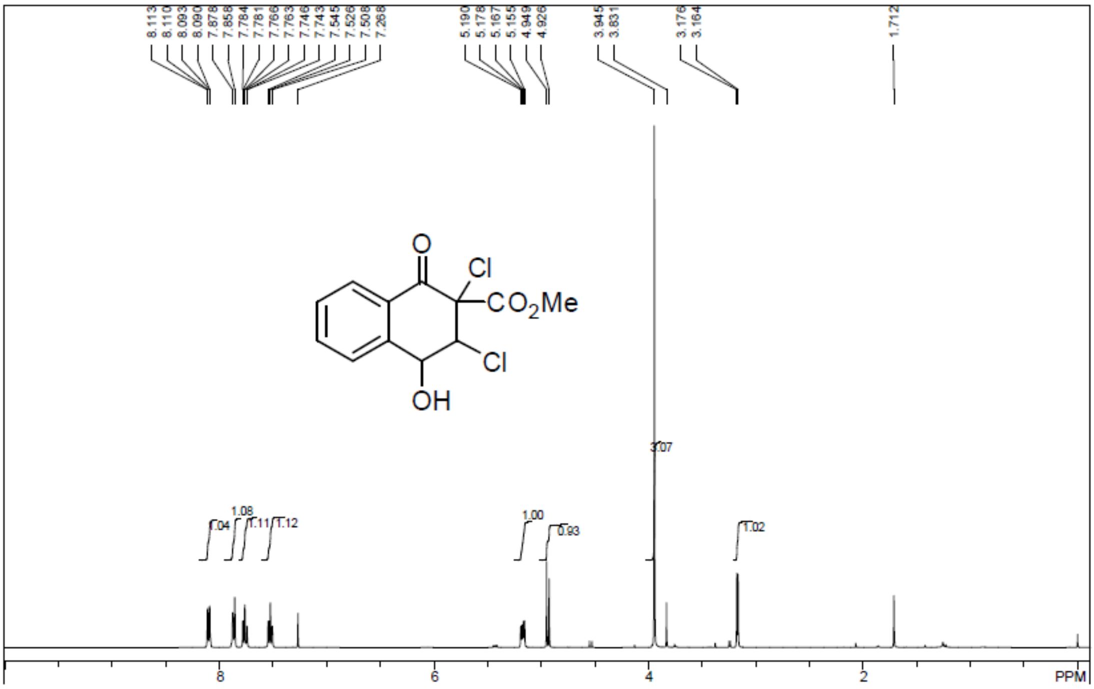

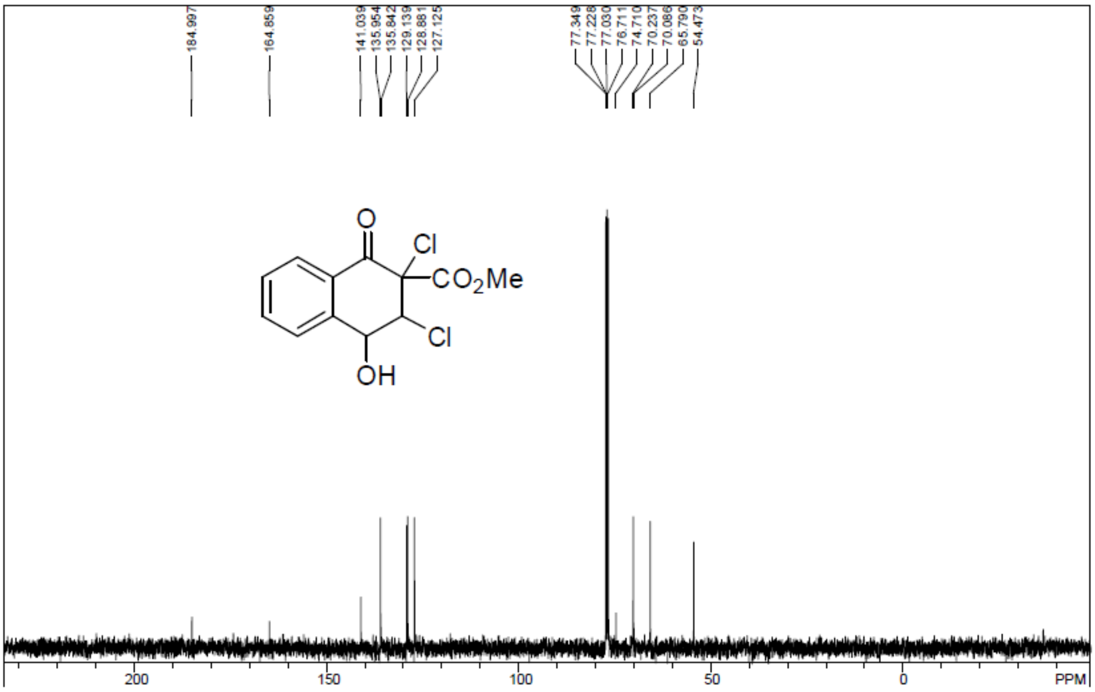

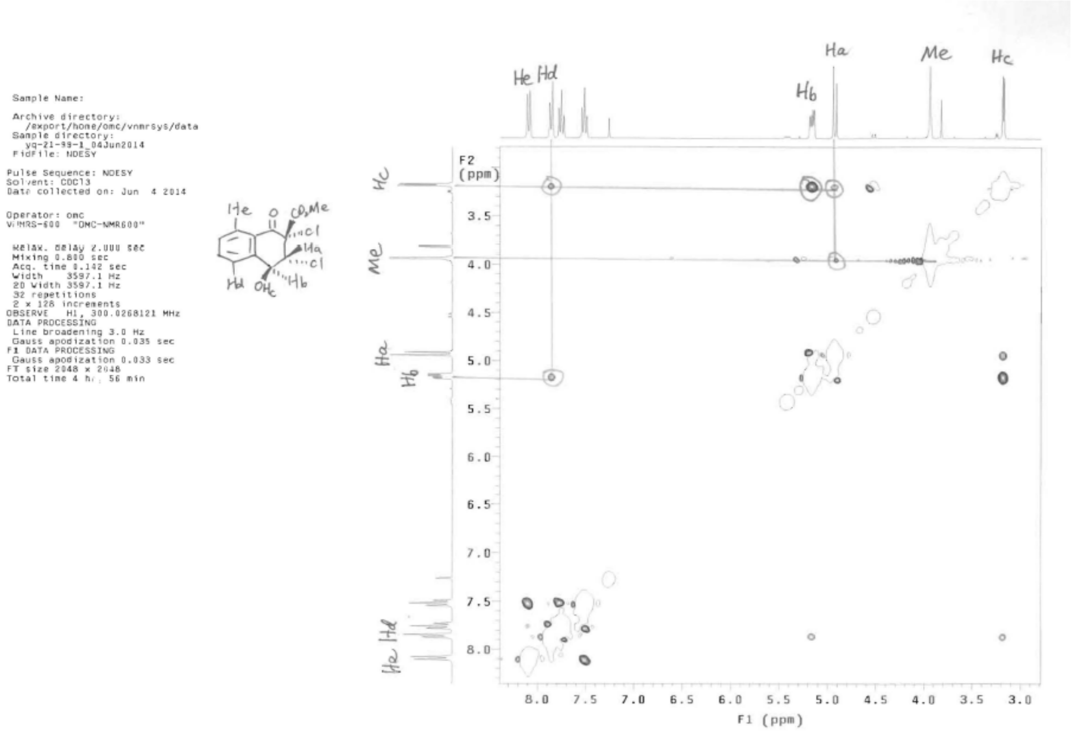
Sample Name:
Archive directory:
/export/home/omc/vnmrsys/data
Sample directory:
yq-21-99-1_04Jun2014
FidFile: NDESY
Pulse Sequence: NDESY
Solvent: CDCl3
Data collected on: Jun 4 2014
Operator: omc
VNMRS-600 "OMC-NMR600"
Relax. delay 2.000 sec
Mixing 0.800 sec
Acq. time 0.142 sec
Width 3597.1 Hz
2D Width 3597.1 Hz
32 repetitions
2 x 128 increments
OBSERVE H1, 300.0268121 MHz
DATA PROCESSING
Line broadening 3.0 Hz
Gauss apodization 0.035 sec
F1 DATA PROCESSING
Gauss apodization 0.033 sec
FT size 2048 x 2048
Total time 4 hr, 56 min
He
Hd
CO2Me
Cl
Ha
Hb
OHc
Me
Hc
F2 (ppm)
F1 (ppm)

5. Copies of HPLC spectra

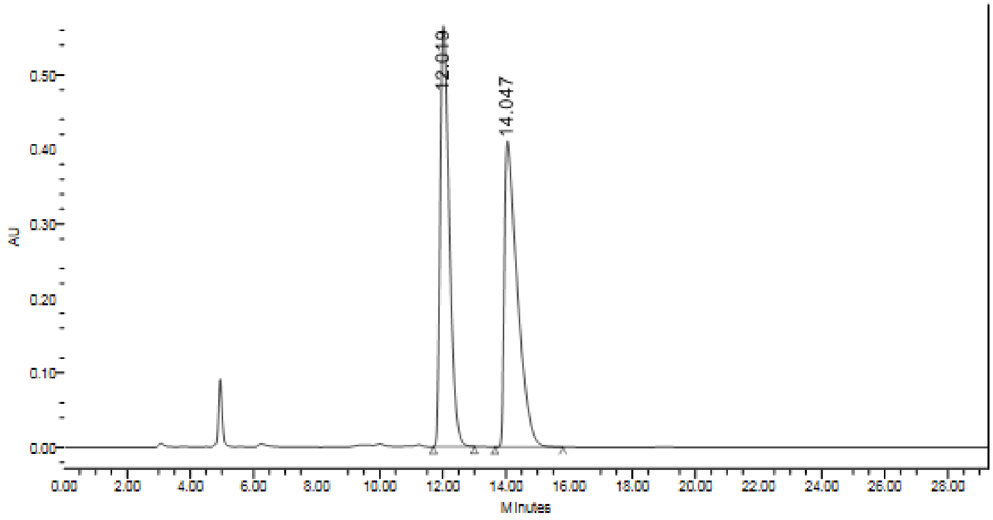

	RT (min)	Area (µV*sec)	% Area	Height (µV)	% Height
1	12.019	11365177	49.00	565461	57.89
2	14.047	11827174	51.00	411247	42.11

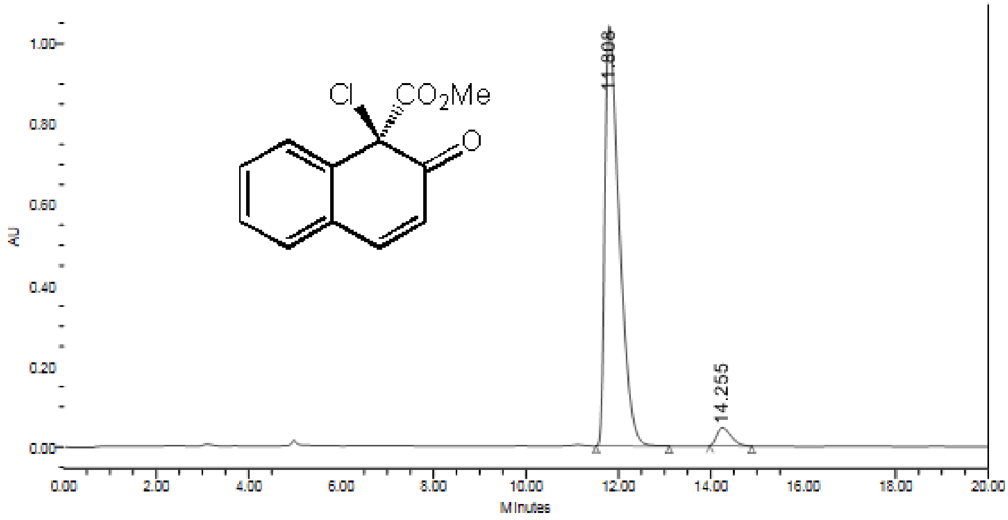

	RT (min)	Area (µV*sec)	% Area	Height (µV)	% Height
1	11.808	22533161	95.96	1043509	95.88
2	14.255	948790	4.04	44814	4.12

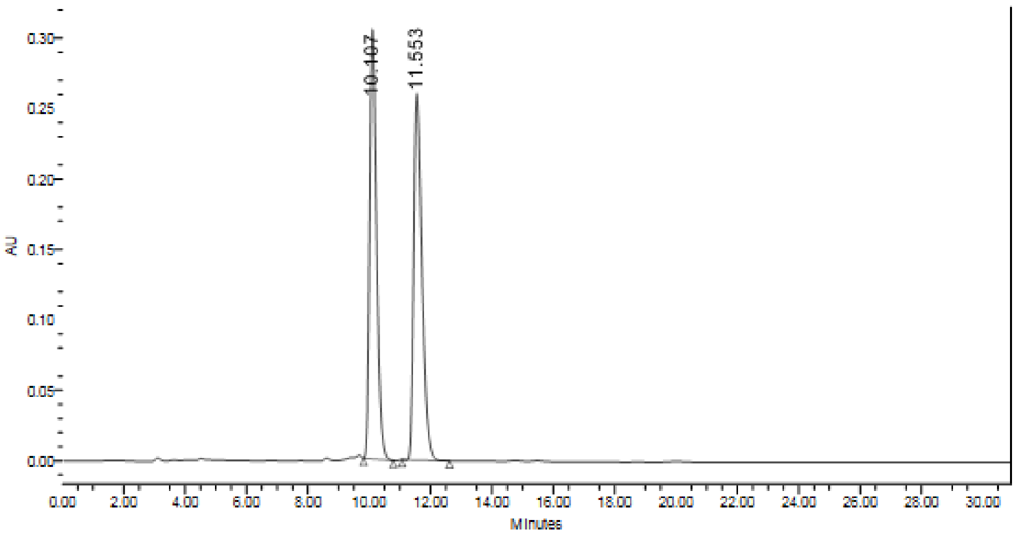

	RT (min)	Area (µV*sec)	% Area	Height (µV)	% Height
1	10.107	4796642	49.62	305747	54.03
2	11.553	4869944	50.38	260129	45.97

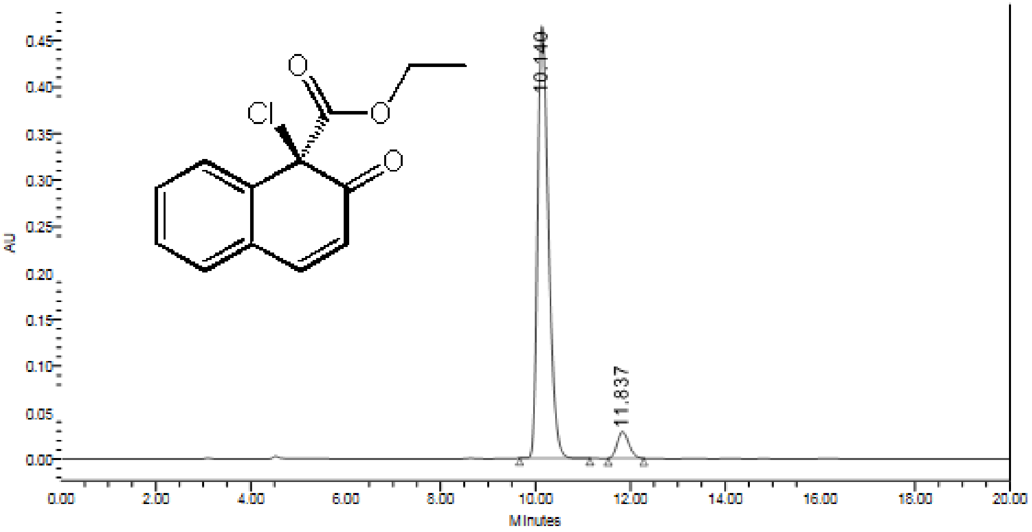

	RT (min)	Area (µV*sec)	% Area	Height (µV)	% Height
1	10.140	7408867	93.77	465188	94.22
2	11.837	492375	6.23	28542	5.78

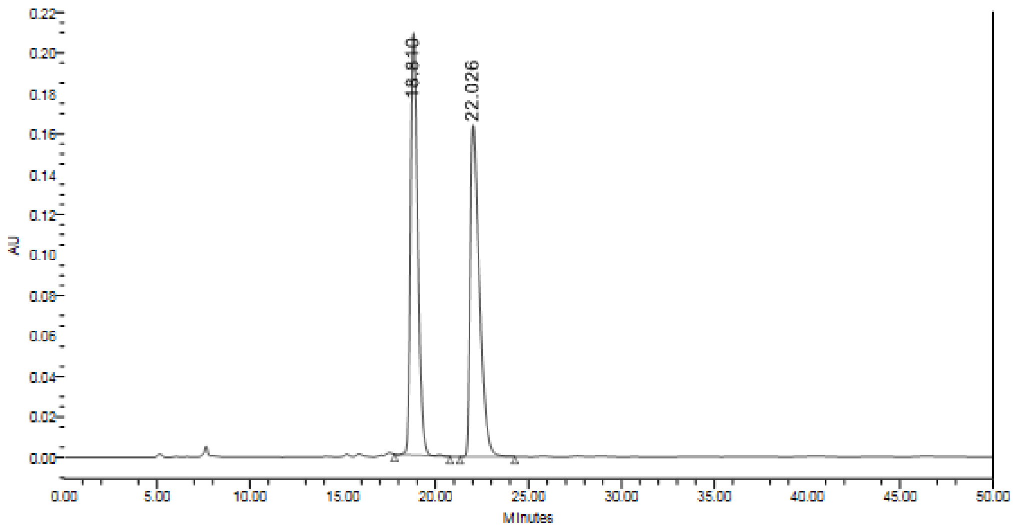

	RT (min)	Area (µV*sec)	% Area	Height (µV)	% Height
1	18.810	5569938	49.72	208702	55.98
2	22.026	5633799	50.28	164089	44.02

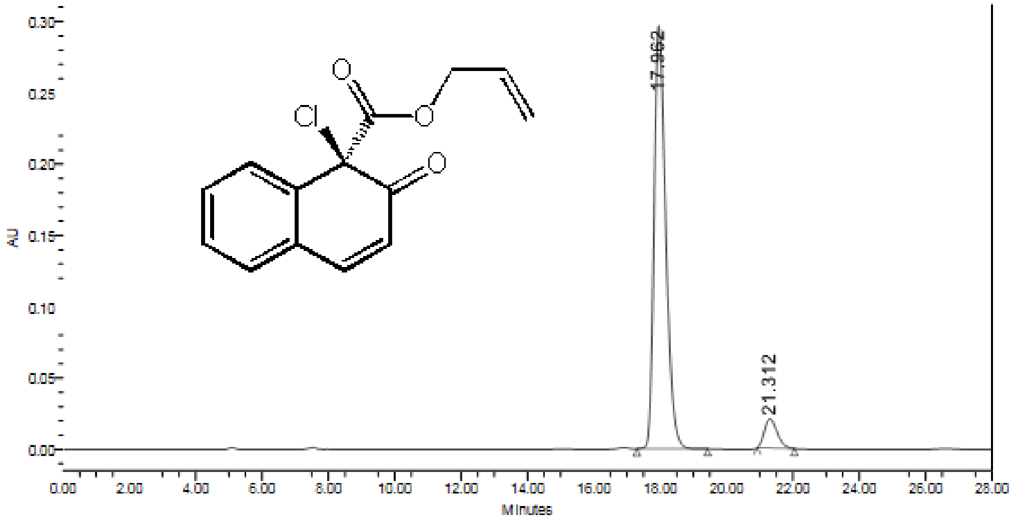

	RT (min)	Area (µV*sec)	% Area	Height (µV)	% Height
1	17.962	7200748	93.00	296744	93.60
2	21.312	542326	7.00	20307	6.40

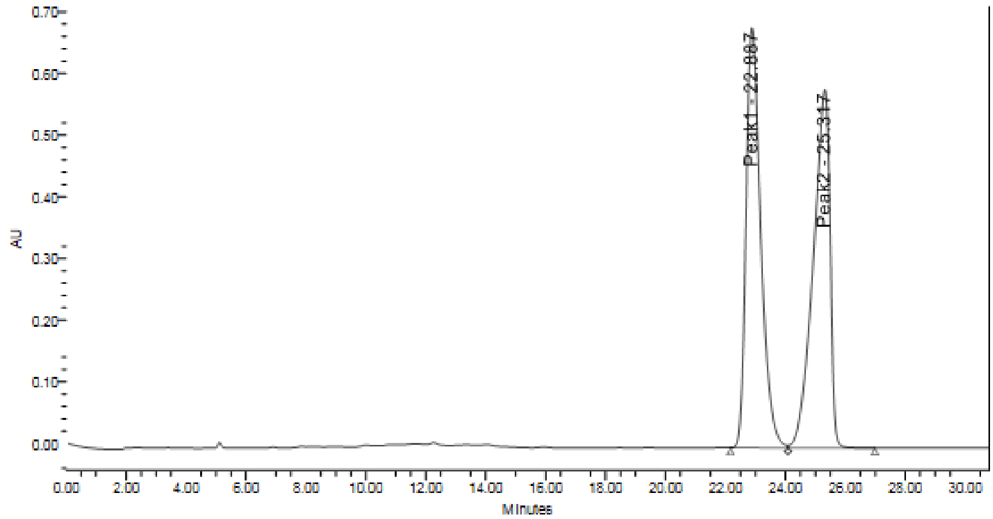

	Peak Name	RT (min)	Area (µV*sec)	% Area	Height (µV)	% Height
1	Peak1	22.887	23134401	50.61	680963	53.94
2	Peak2	25.317	22580891	49.39	581460	46.06

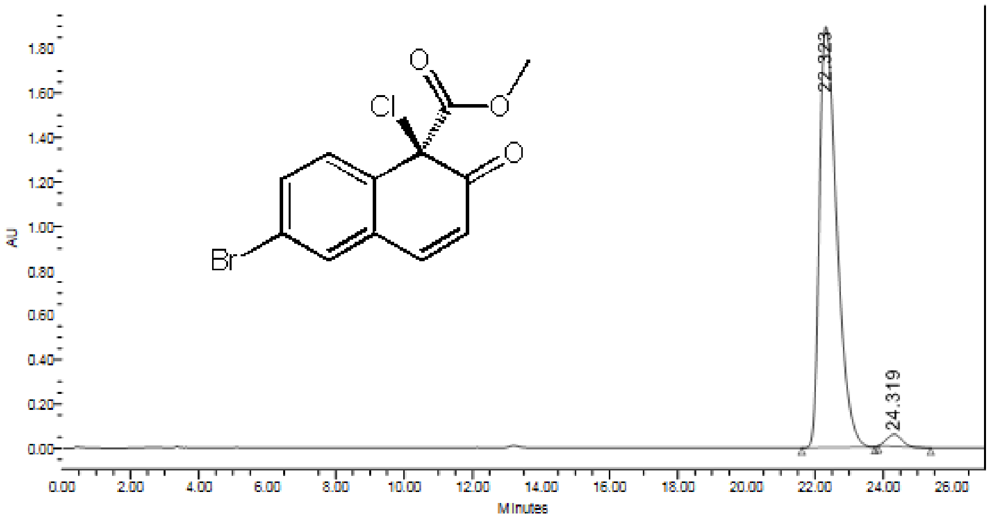

	RT (min)	Area (µV*sec)	% Area	Height (µV)	% Height
1	22.323	66891199	97.56	1895184	97.21
2	24.319	1670645	2.44	54369	2.79

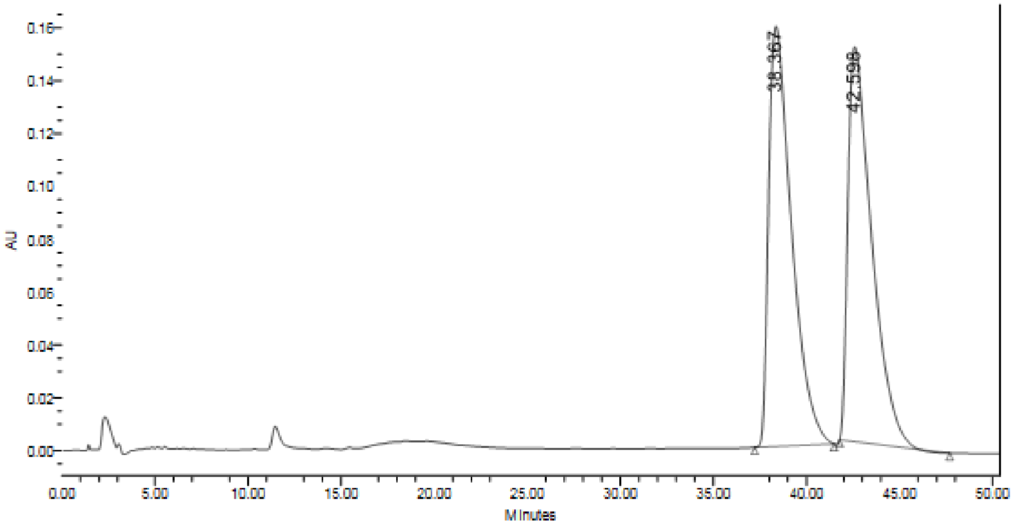

	RT (min)	Area (µV*sec)	% Area	Height (µV)	% Height
1	38.367	13590569	50.48	159063	51.55
2	42.598	13330754	49.52	149479	48.45

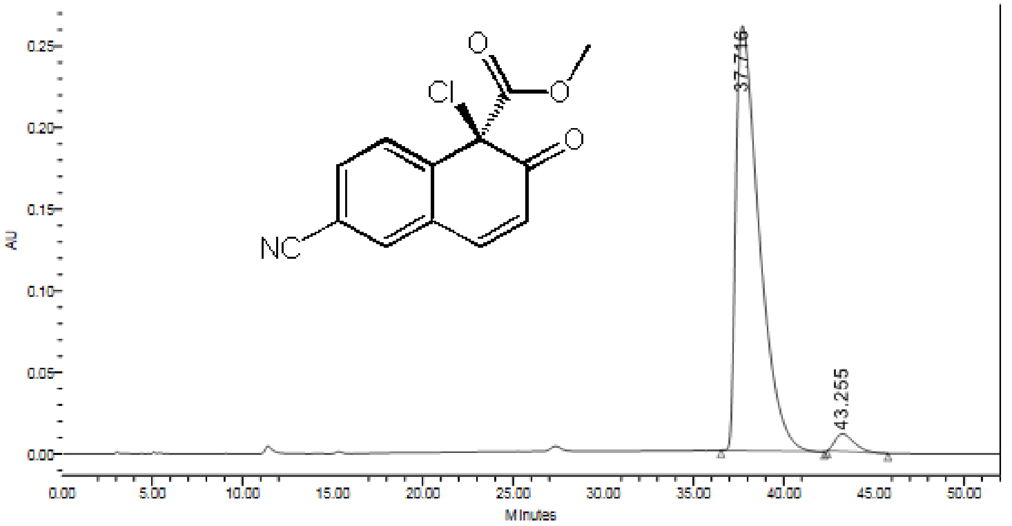

	RT (min)	Area (µV*sec)	% Area	Height (µV)	% Height
1	37.716	23389922	96.78	260136	96.11
2	43.255	776967	3.22	10538	3.89

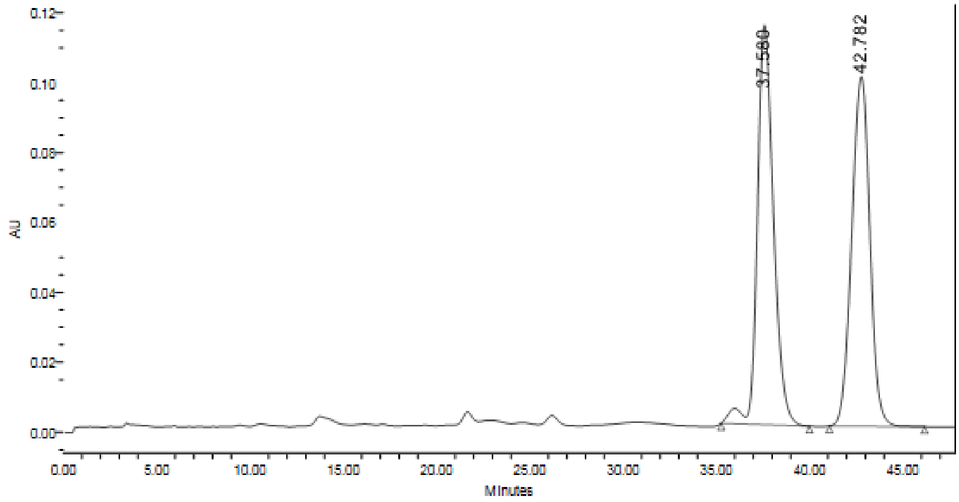

	RT (min)	Area (µV*sec)	% Area	Height (µV)	% Height
1	37.580	6932188	50.61	114376	53.36
2	42.782	6764048	49.39	99977	46.64

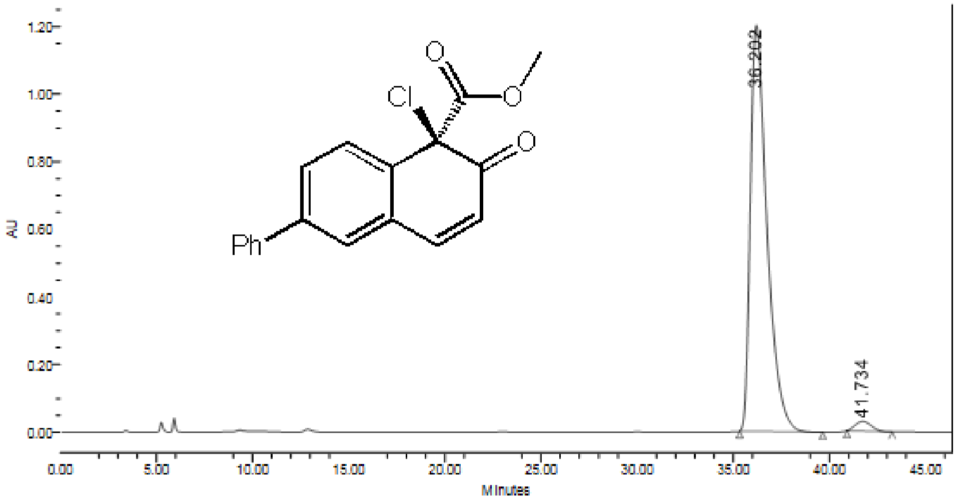

	RT (min)	Area (µV*sec)	% Area	Height (µV)	% Height
1	36.202	72297883	97.74	1201744	97.68
2	41.734	1669028	2.26	28520	2.32

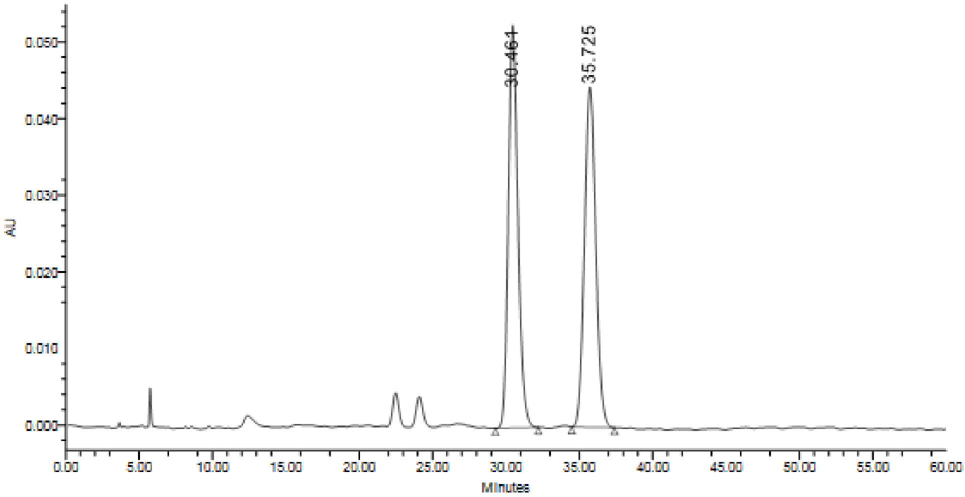

	RT (min)	Area (µV*sec)	% Area	Height (µV)	% Height
1	30.461	2406015	50.30	52607	54.24
2	35.725	2377418	49.70	44384	45.76

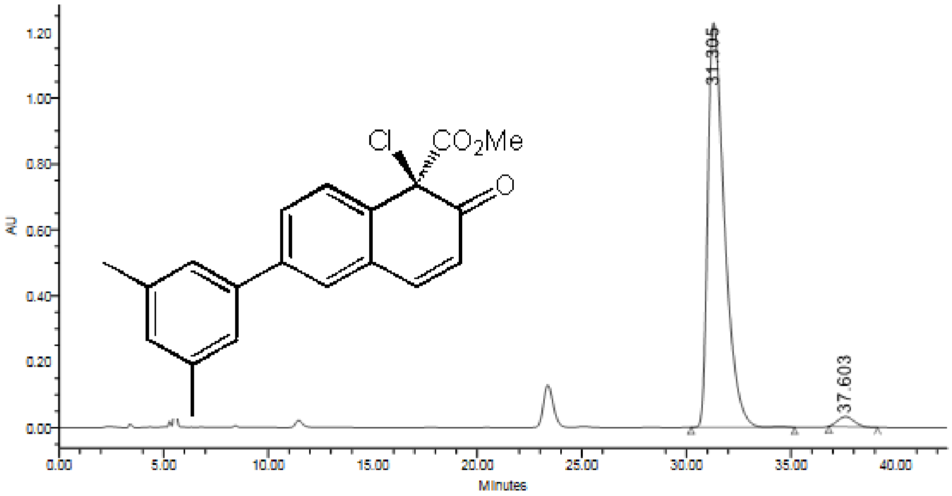

	RT (min)	Area (µV*sec)	% Area	Height (µV)	% Height
1	31.305	68005536	97.60	1227232	97.60
2	37.603	1669203	2.40	30223	2.40

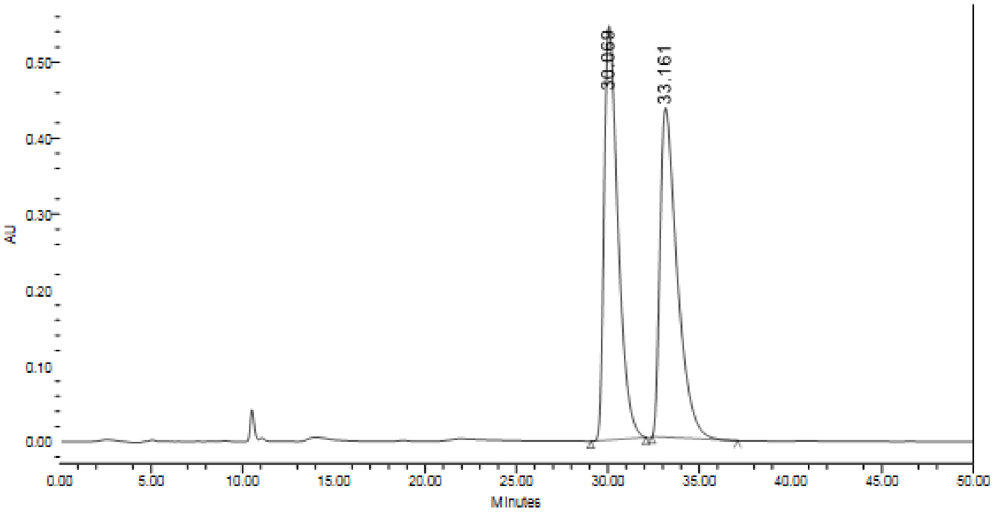

	RT (min)	Area (µV*sec)	% Area	Height (µV)	% Height
1	30.069	28604876	50.17	545690	55.74
2	33.161	28410751	49.83	433389	44.26

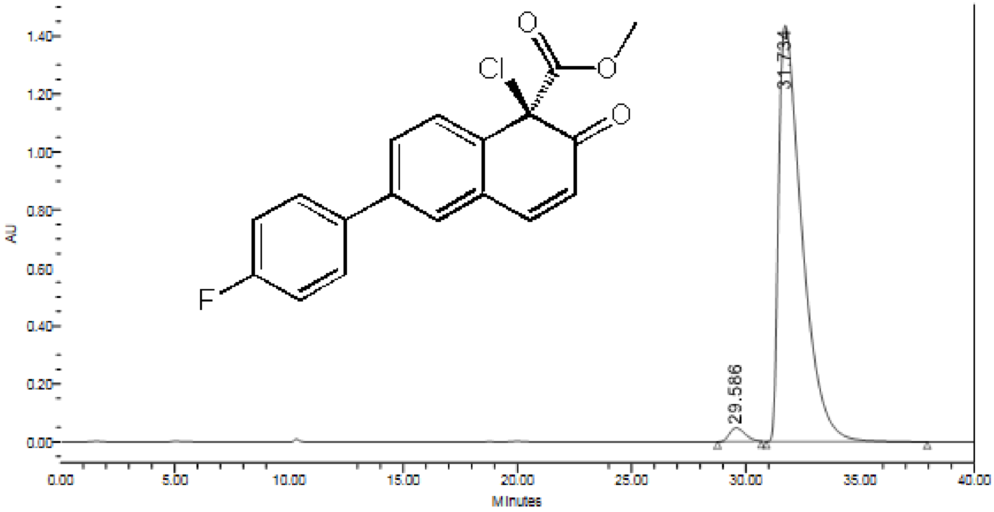

	RT (min)	Area (µV*sec)	% Area	Height (µV)	% Height
1	29.586	2213842	2.13	47029	3.17
2	31.734	101715459	97.87	1435469	96.83

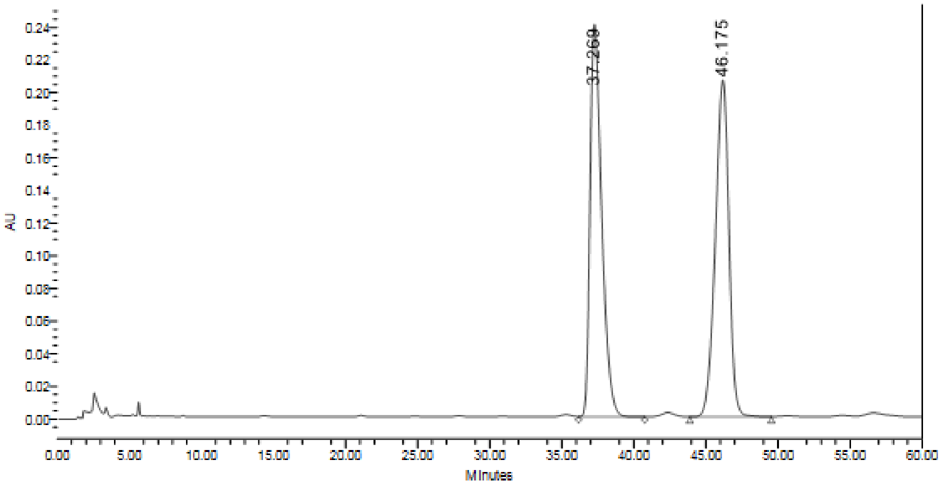

	RT (min)	Area (µV*sec)	% Area	Height (µV)	% Height
1	37.269	13454777	49.86	240363	53.81
2	46.175	13528377	50.14	206292	46.19

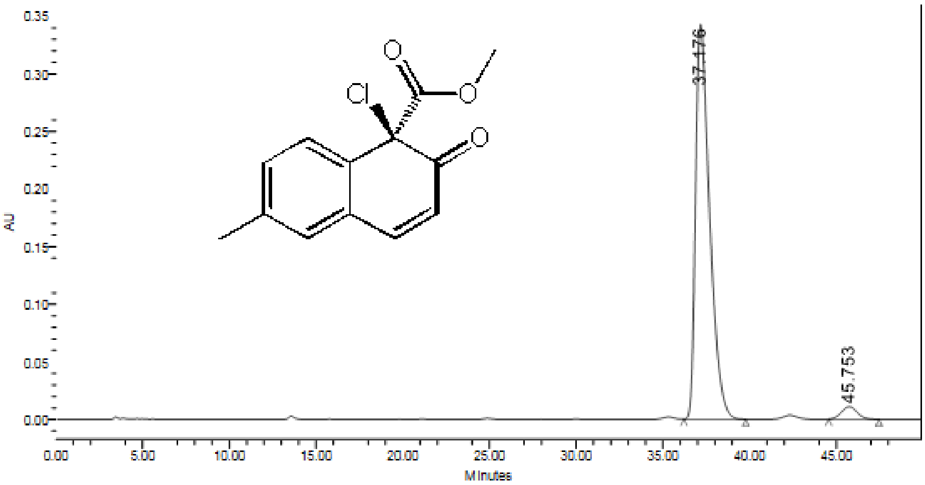

	RT (min)	Area (µV*sec)	% Area	Height (µV)	% Height
1	37.176	19311577	96.55	342900	96.91
2	45.753	689527	3.45	10935	3.09

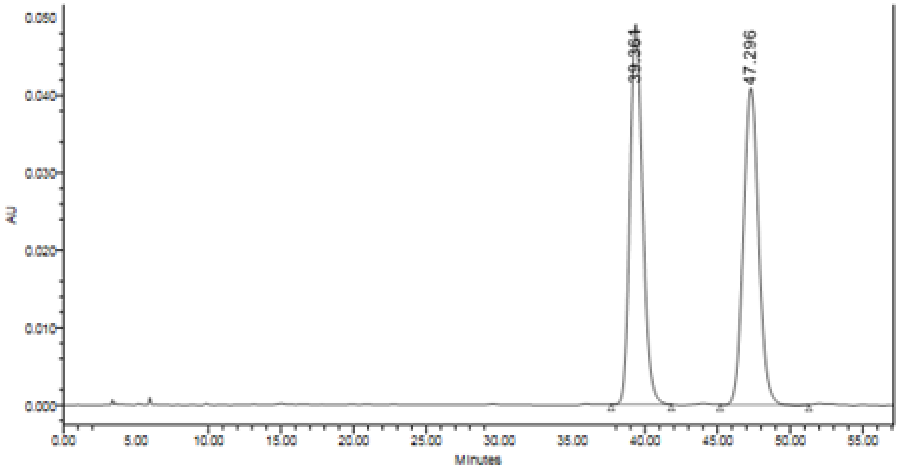

	RT (min)	Area (µV*sec)	% Area	Height (µV)	% Height
1	39.361	2982801	49.98	49122	54.58
2	47.296	2984862	50.02	40885	45.42

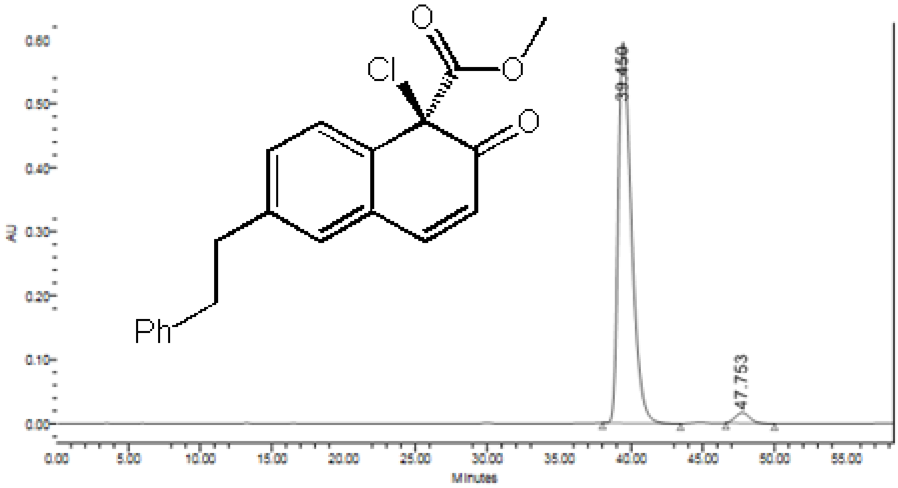

	RT (min)	Area (µV*sec)	% Area	Height (µV)	% Height
1	39.450	38190025	97.09	594775	97.32
2	47.753	1143550	2.91	16393	2.68

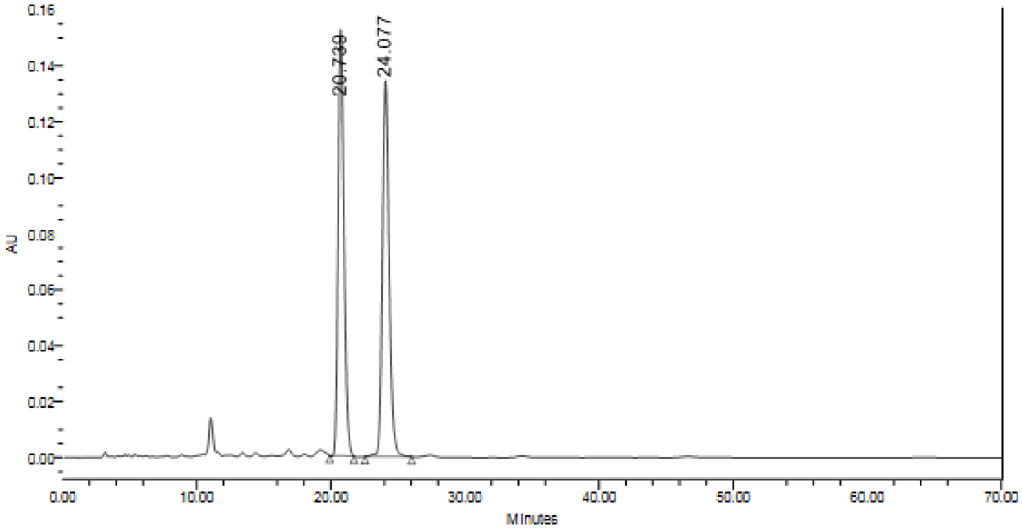

	RT (min)	Area (µV*sec)	% Area	Height (µV)	% Height
1	20.739	4582873	49.38	152392	53.13
2	24.077	4698772	50.62	134431	46.87

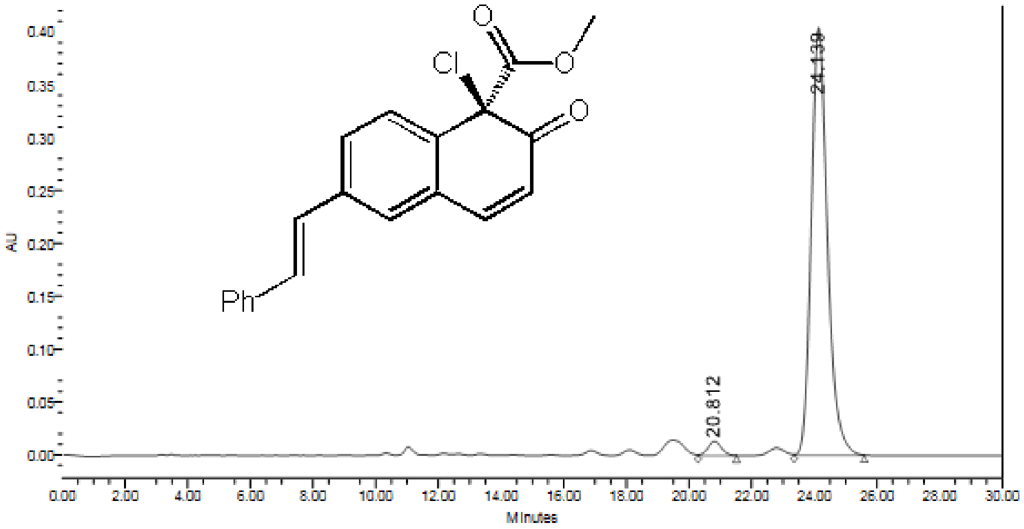

	RT (min)	Area (µV*sec)	% Area	Height (µV)	% Height
1	20.812	403708	2.70	13466	3.22
2	24.139	14527566	97.30	404470	96.78

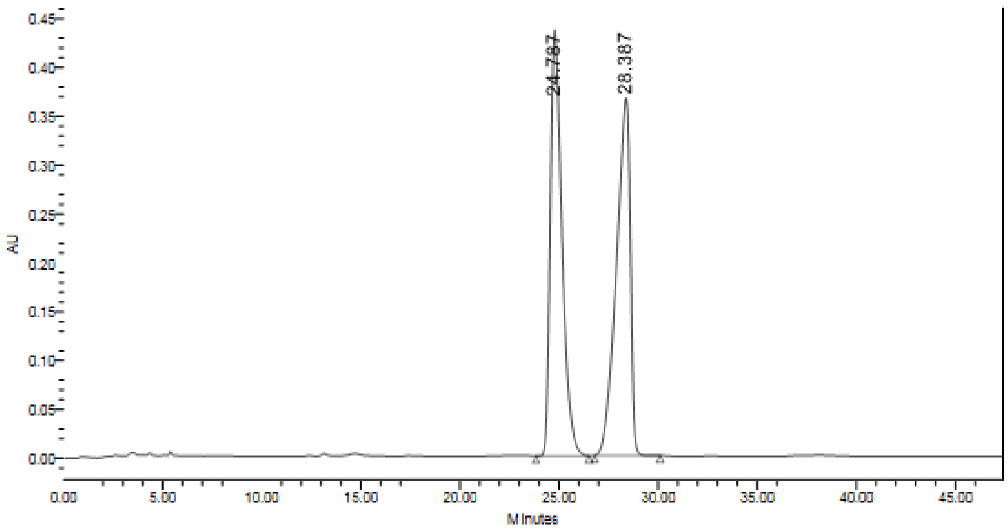

	RT (min)	Area (μV*sec)	% Area	Height (μV)	% Height
1	24.787	17846615	50.04	436463	54.39
2	28.387	17820855	49.96	366054	45.61

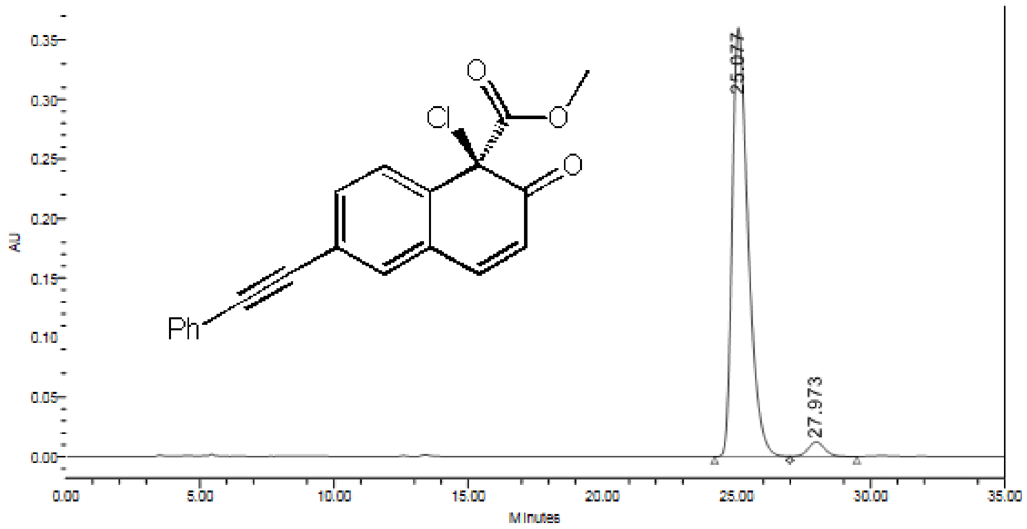

	RT (min)	Area (μV*sec)	% Area	Height (μV)	% Height
1	25.077	14825846	96.47	360412	96.68
2	27.973	541853	3.53	12385	3.32

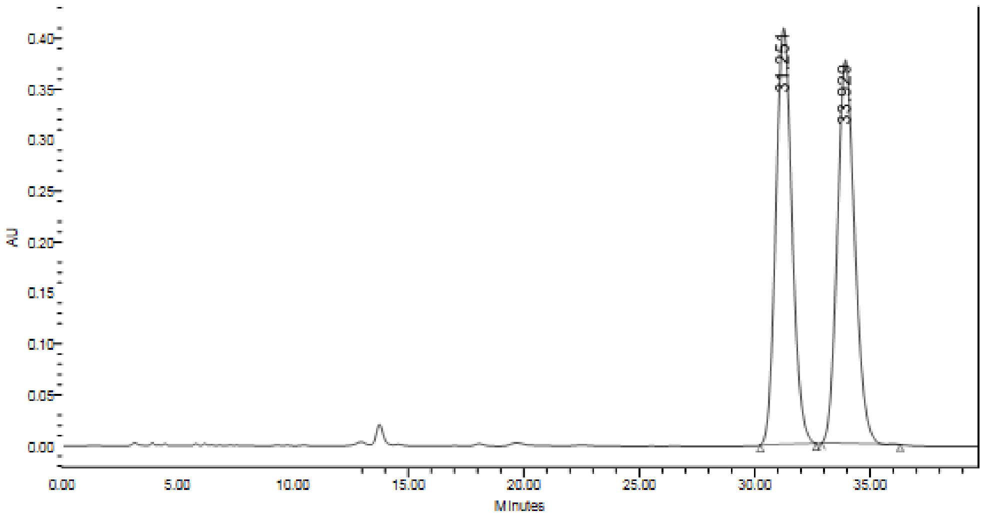

	RT (min)	Area (µV*sec)	% Area	Height (µV)	% Height
1	31.251	19850934	50.15	408669	52.06
2	33.929	19734986	49.85	376259	47.94

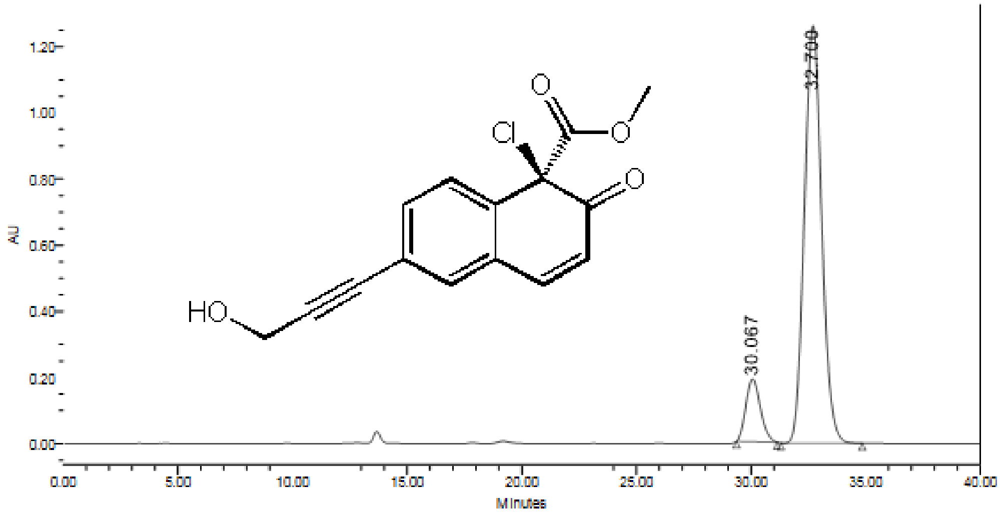

	RT (min)	Area (µV*sec)	% Area	Height (µV)	% Height
1	30.067	8294196	11.14	188885	13.02
2	32.700	66192997	88.86	1261812	86.98

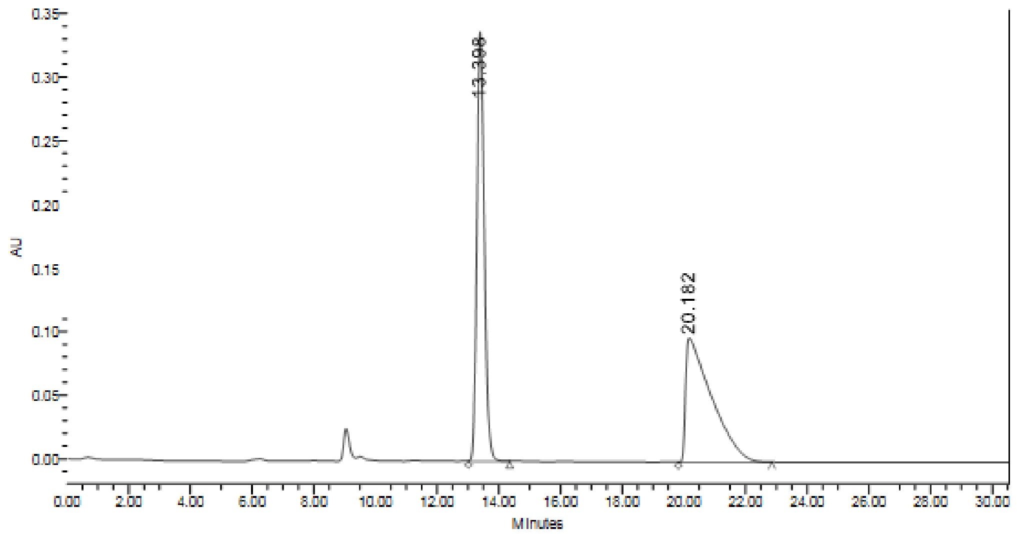

	RT (min)	Area (µV*sec)	% Area	Height (µV)	% Height
1	13.398	5618335	49.76	337321	77.60
2	20.182	5672134	50.24	97347	22.40

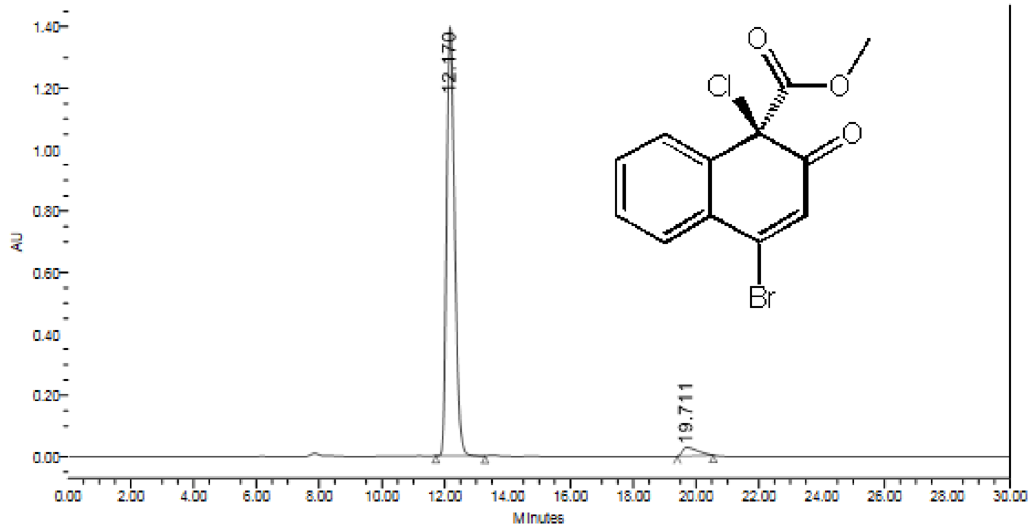

	RT (min)	Area (µV*sec)	% Area	Height (µV)	% Height
1	12.170	24879169	96.29	1397259	98.04
2	19.711	959324	3.71	27867	1.96

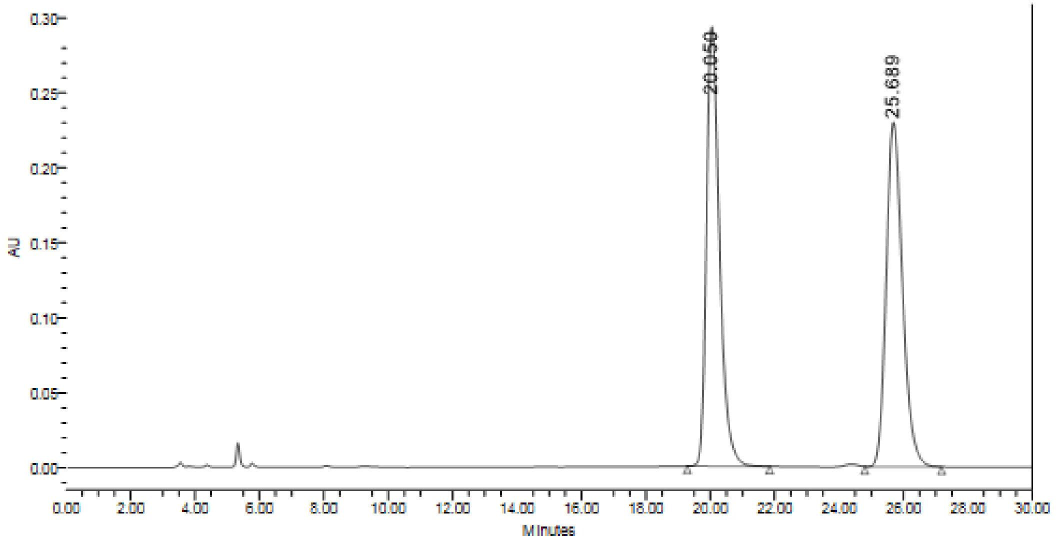

	RT (min)	Area (µV*sec)	% Area	Height (µV)	% Height
1	20.050	8194779	50.38	293221	56.06
2	25.689	8069749	49.62	229817	43.94

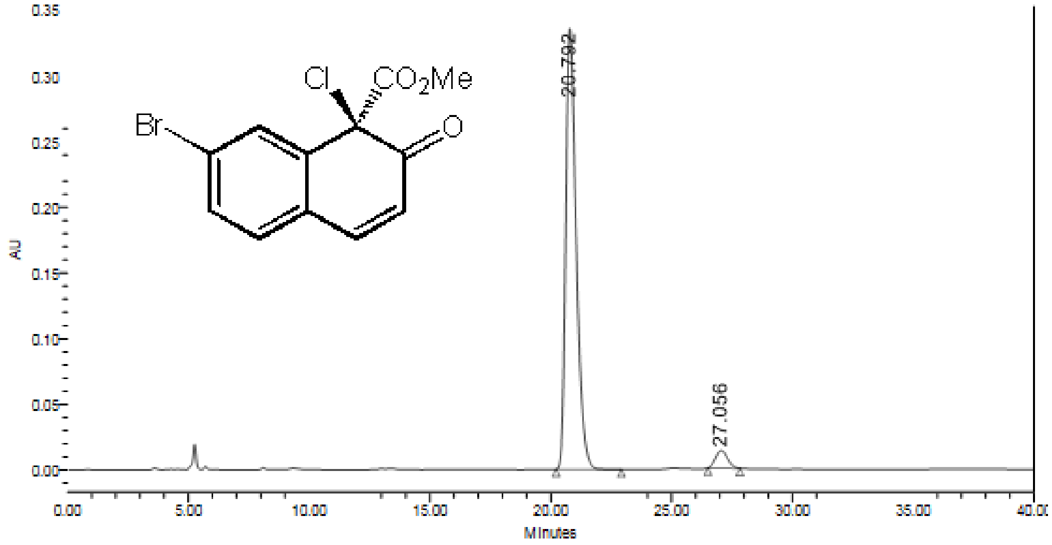

	RT (min)	Area (µV*sec)	% Area	Height (µV)	% Height
1	20.792	10076345	95.58	335996	96.15
2	27.056	466275	4.42	13450	3.85

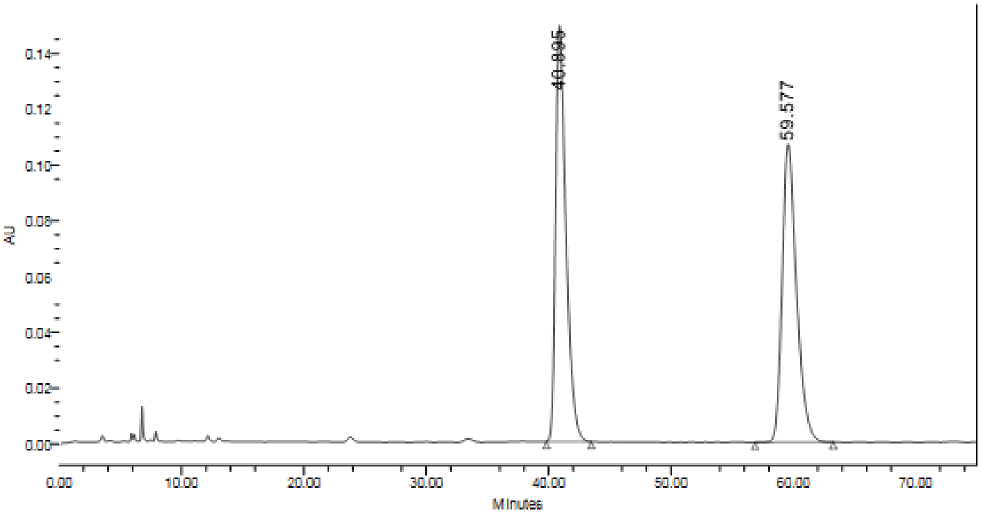

	RT (min)	Area (µV*sec)	% Area	Height (µV)	% Height
1	40.895	8844978	50.23	149243	58.28
2	59.577	8762756	49.77	106845	41.72

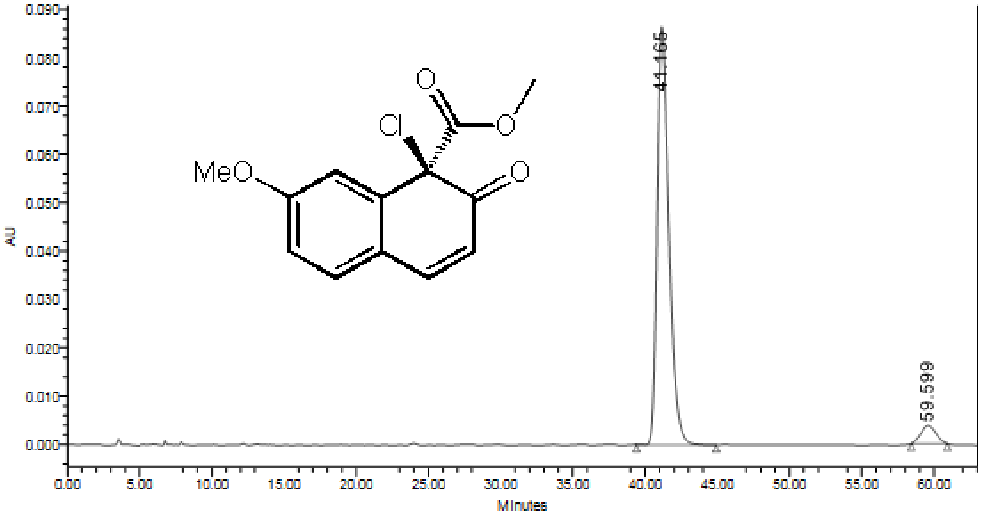

	RT (min)	Area (µV*sec)	% Area	Height (µV)	% Height
1	41.165	4897699	94.86	86454	95.89
2	59.599	265475	5.14	3703	4.11

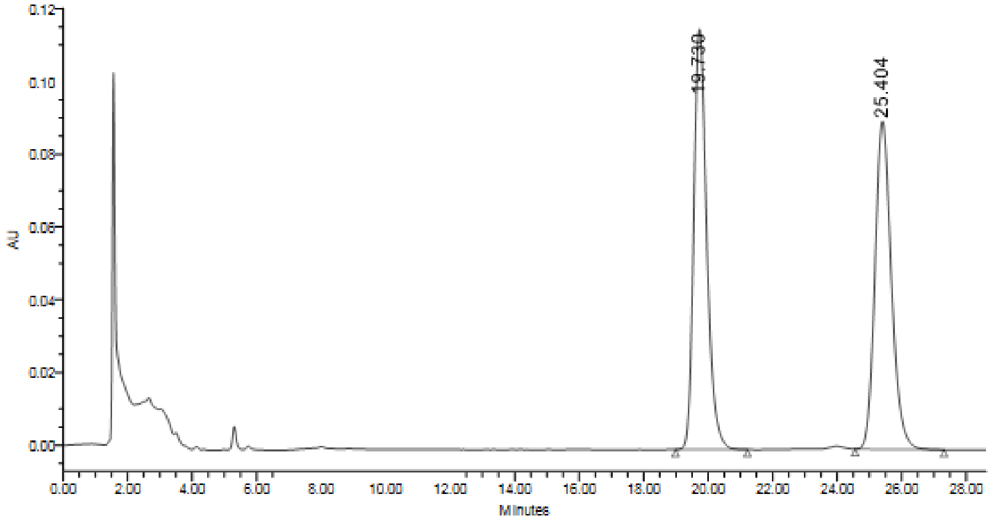

	RT (min)	Area (µV*sec)	% Area	Height (µV)	% Height
1	19.730	3231184	50.23	115637	56.19
2	25.404	3200988	49.77	90164	43.81

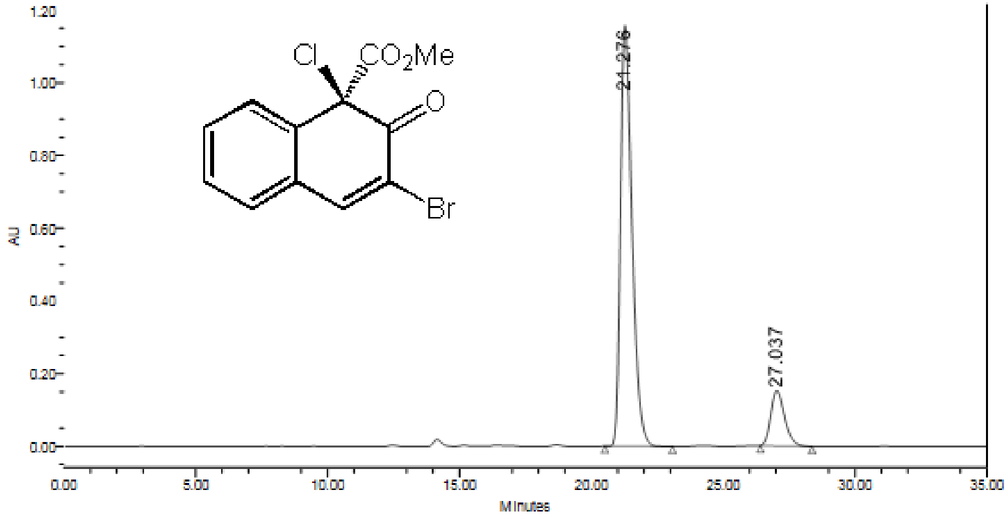

	RT (min)	Area (µV*sec)	% Area	Height (µV)	% Height
1	21.276	35044781	86.42	1158093	88.47
2	27.037	5506585	13.58	150916	11.53

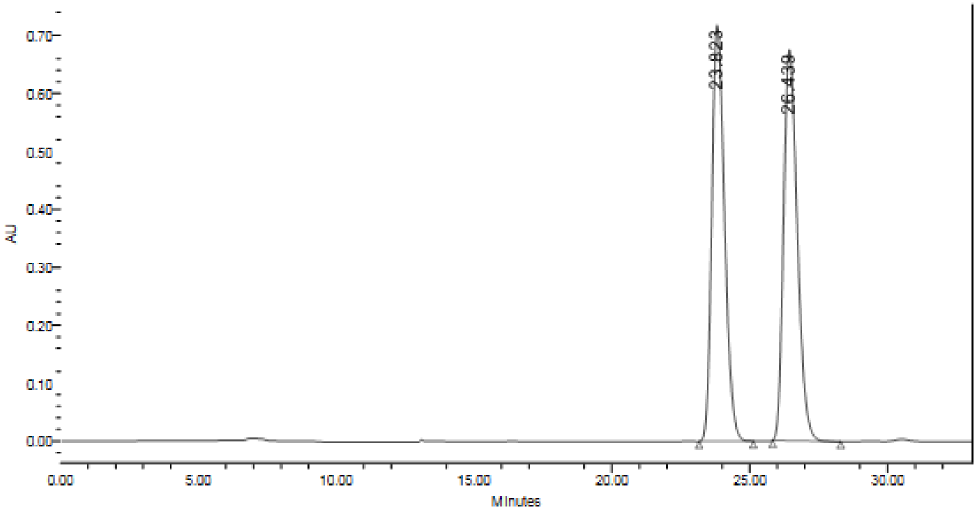

	RT (min)	Area (µV*sec)	% Area	Height (µV)	% Height
1	23.823	23081759	49.83	718170	51.57
2	26.439	23239145	50.17	674502	48.43

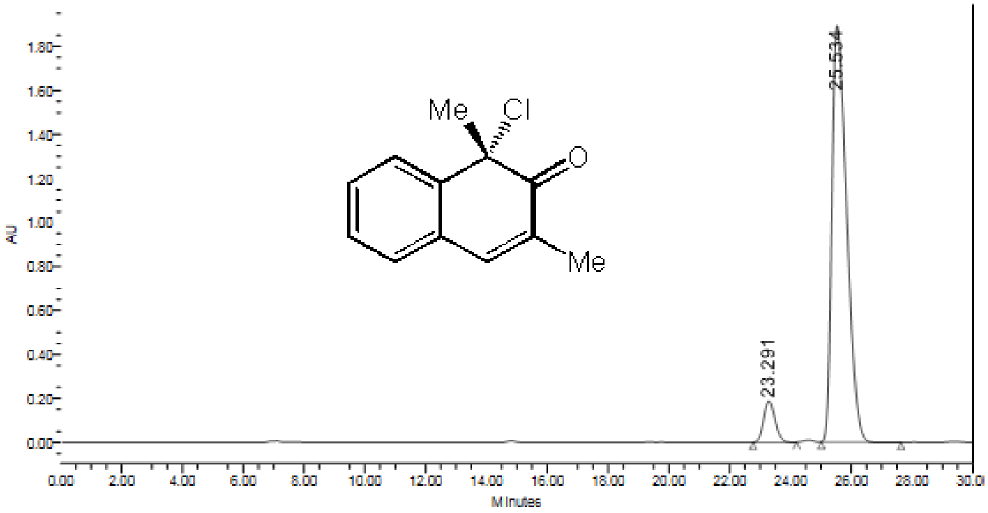

	RT (min)	Area (µV*sec)	% Area	Height (µV)	% Height
1	23.291	4873814	7.08	187938	9.04
2	25.534	63981644	92.92	1892085	90.96

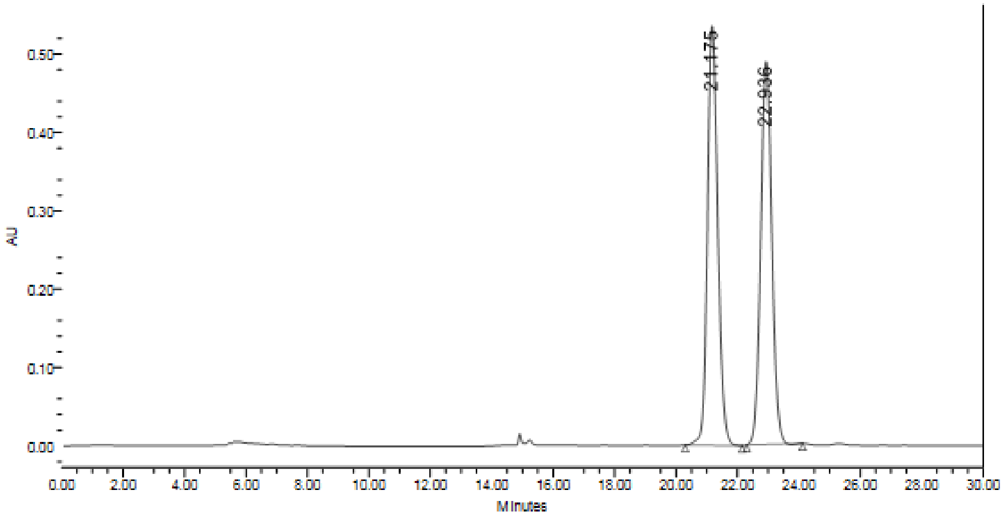

	RT (min)	Area (µV*sec)	% Area	Height (µV)	% Height
1	21.175	12445155	50.42	536006	52.31
2	22.936	12238047	49.58	488634	47.69

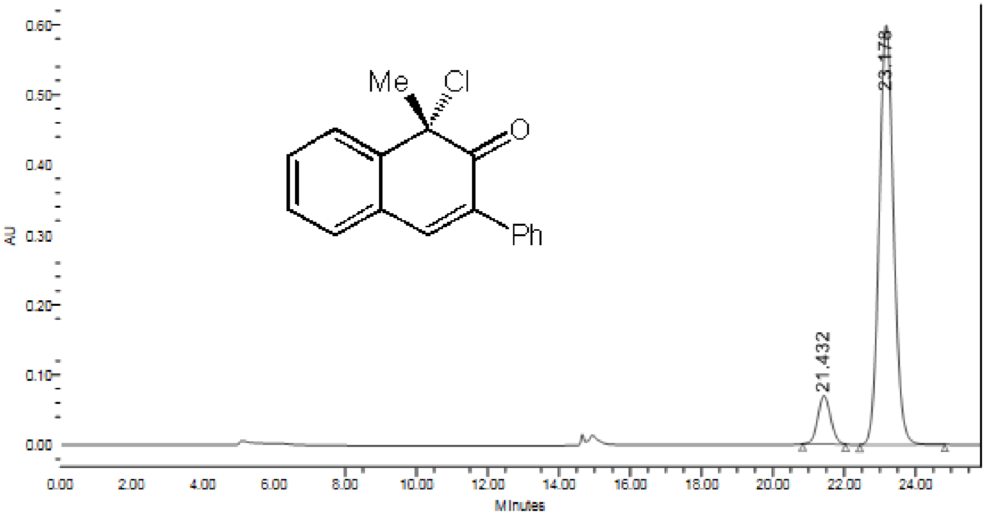

	RT (min)	Area (µV*sec)	% Area	Height (µV)	% Height
1	21.432	1730016	9.31	68738	10.29
2	23.178	16842461	90.69	599046	89.71

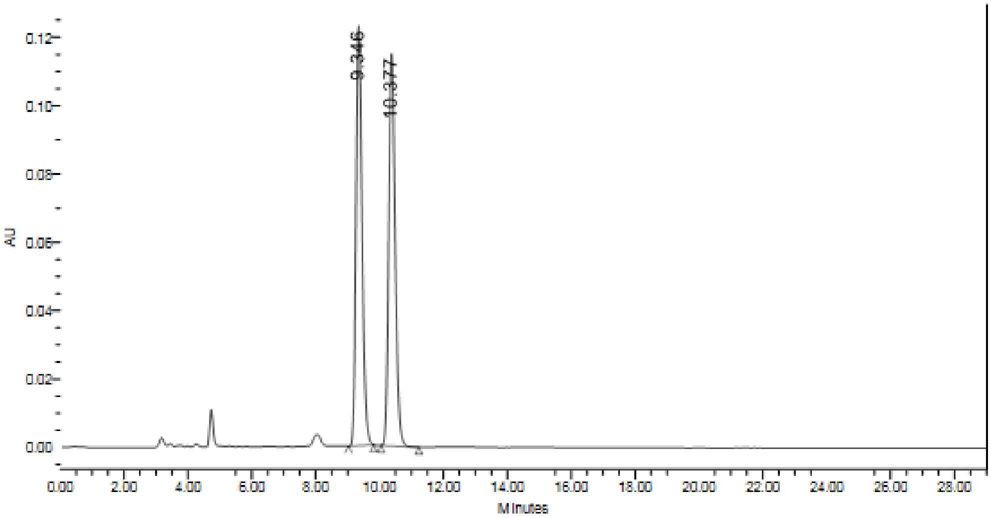

	RT (min)	Area (µV*sec)	% Area	Height (µV)	% Height
1	9.346	1609766	49.82	122987	51.67
2	10.377	1621603	50.18	115029	48.33

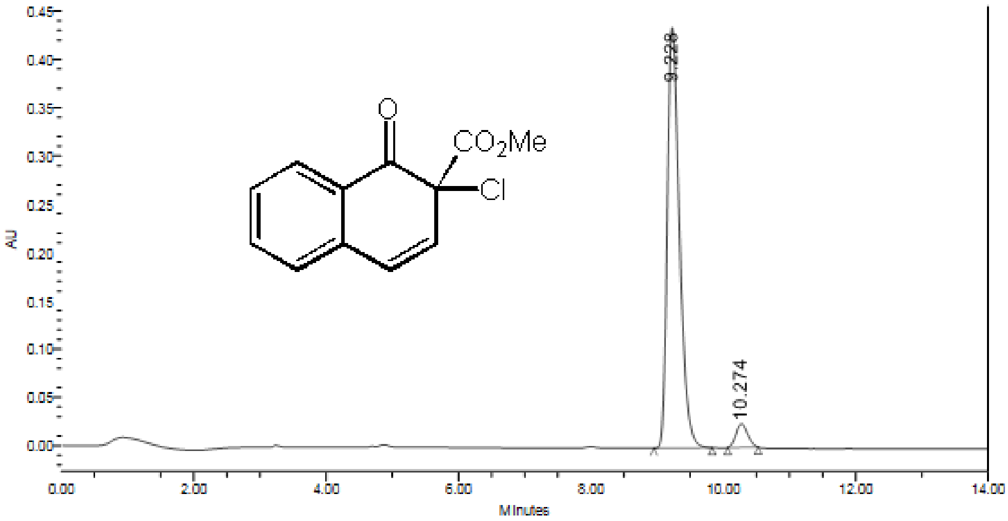

	RT (min)	Area (µV*sec)	% Area	Height (µV)	% Height
1	9.228	5537519	94.91	435633	94.74
2	10.274	296726	5.09	24203	5.26

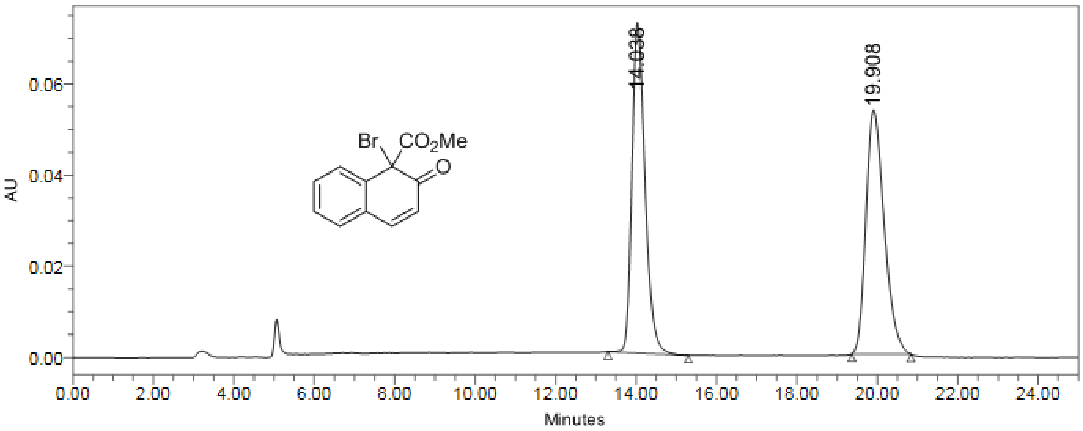

	RT	Area	% Area	Height
1	14.038	1634481	49.16	72502
2	19.908	1690624	50.84	53486

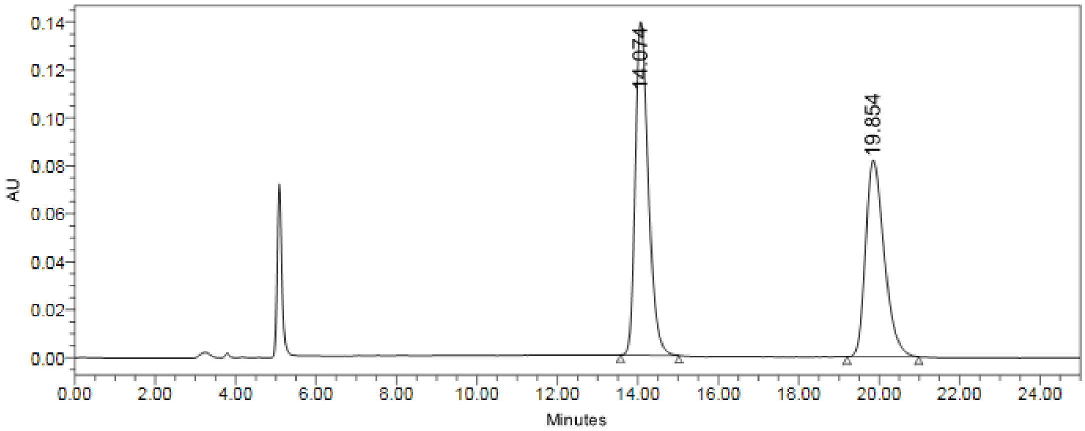

	RT	Area	% Area	Height
1	14.074	3125167	54.31	139069
2	19.854	2629576	45.69	81919

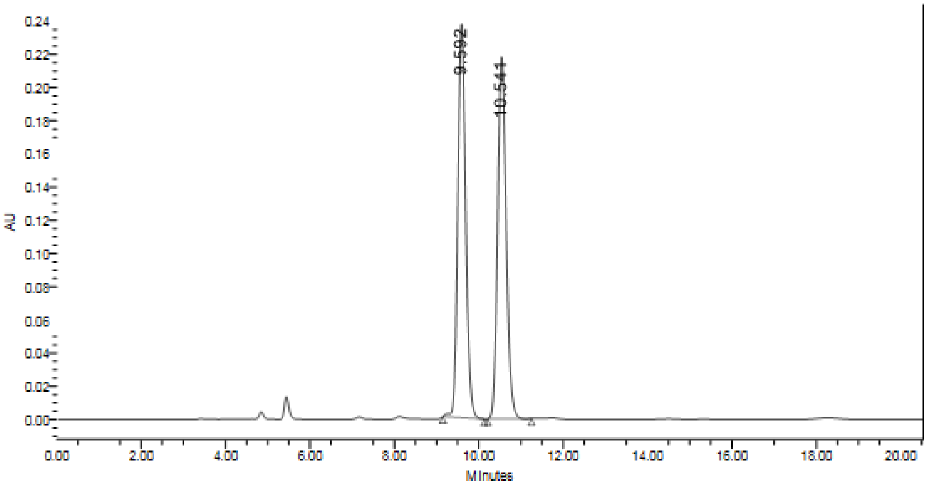

	RT (min)	Area (µV*sec)	% Area	Height (µV)	% Height
1	9.592	3074284	50.14	237888	52.08
2	10.541	3057031	49.86	218857	47.92

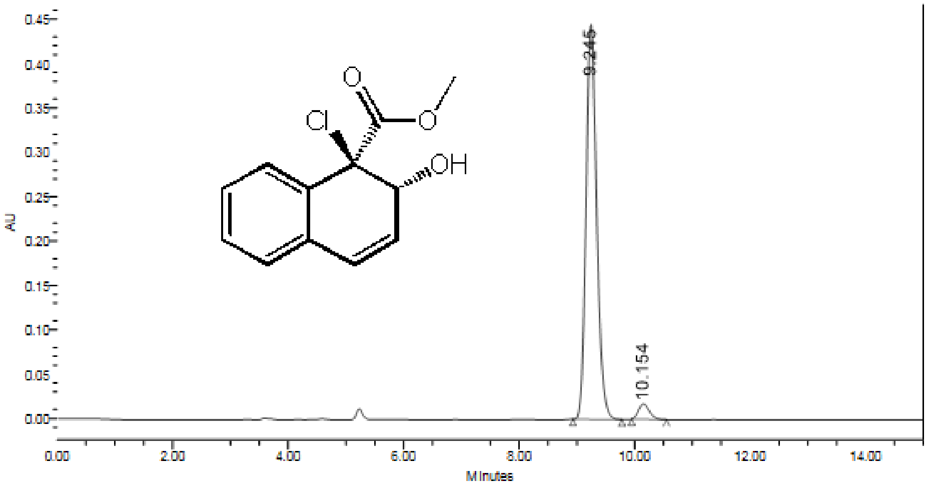

	RT (min)	Area (µV*sec)	% Area	Height (µV)	% Height
1	9.245	5634747	96.17	445529	96.30
2	10.154	224385	3.83	17129	3.70

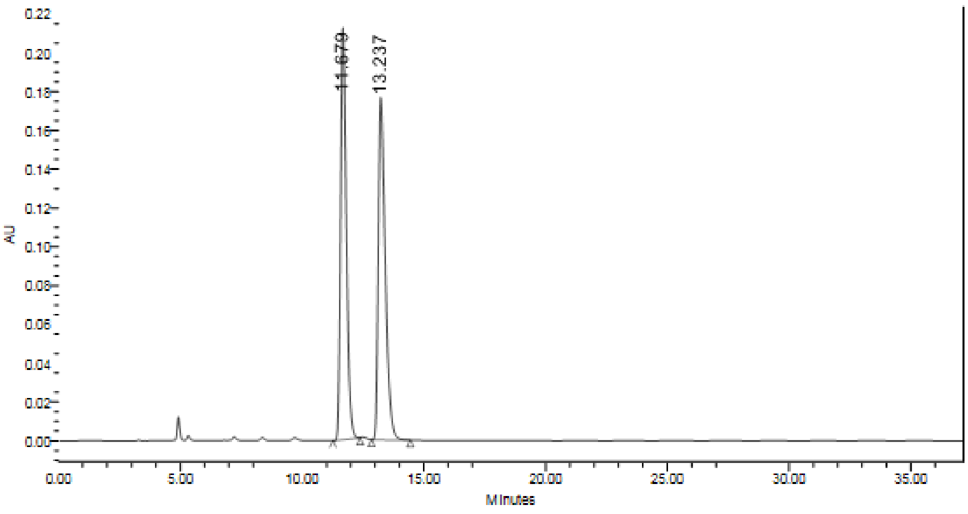

	RT (min)	Area (µV*sec)	% Area	Height (µV)	% Height
1	11.679	3480269	49.72	212462	54.61
2	13.237	3519208	50.28	176623	45.39

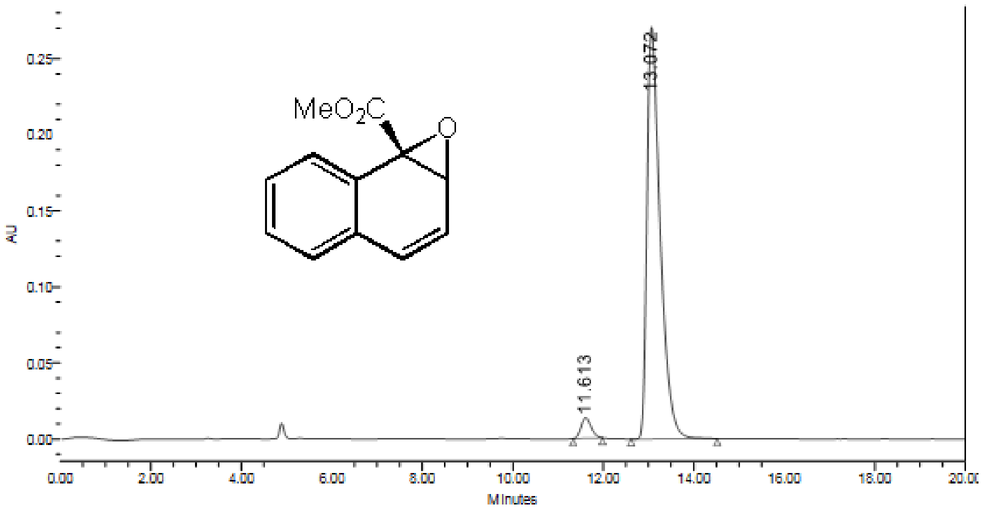

	RT (min)	Area (µV*sec)	% Area	Height (µV)	% Height
1	11.613	212594	3.72	13614	4.78
2	13.072	5498678	96.28	270895	95.22

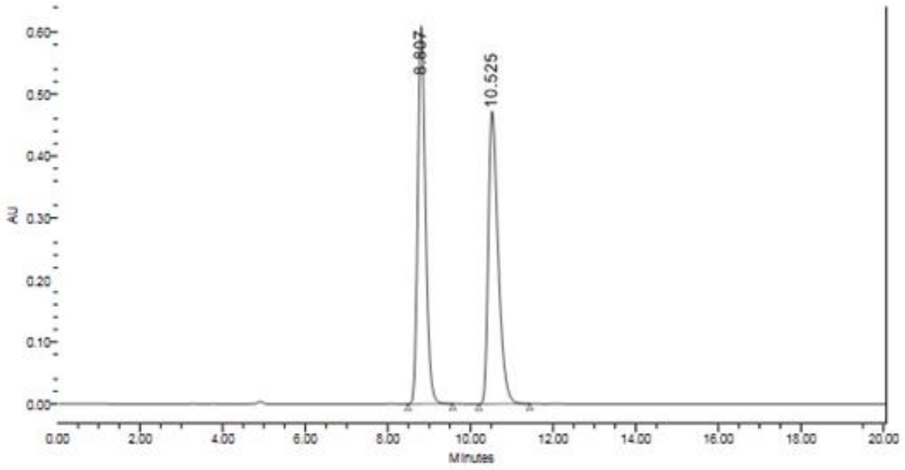

	RT (min)	Area (µV*sec)	% Area	Height (µV)	% Height
1	8.807	7944297	50.45	612259	56.35
2	10.525	7803263	49.55	474246	43.65

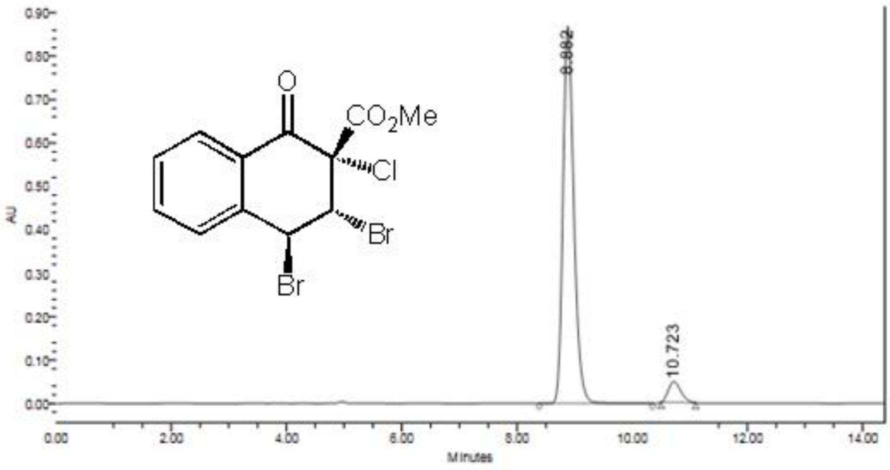

	RT (min)	Area (µV*sec)	% Area	Height (µV)	% Height
1	8.882	11386986	93.98	870034	94.79
2	10.723	729412	6.02	47855	5.21

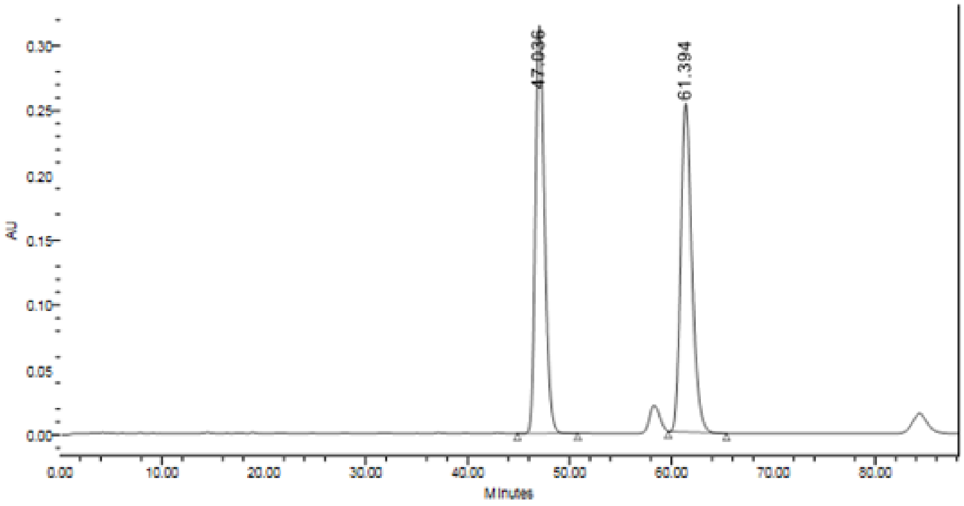

	RT (min)	Area (µV*sec)	% Area	Height (µV)	% Height
1	47.036	19600208	50.34	314631	55.40
2	61.394	19334304	49.66	253265	44.60

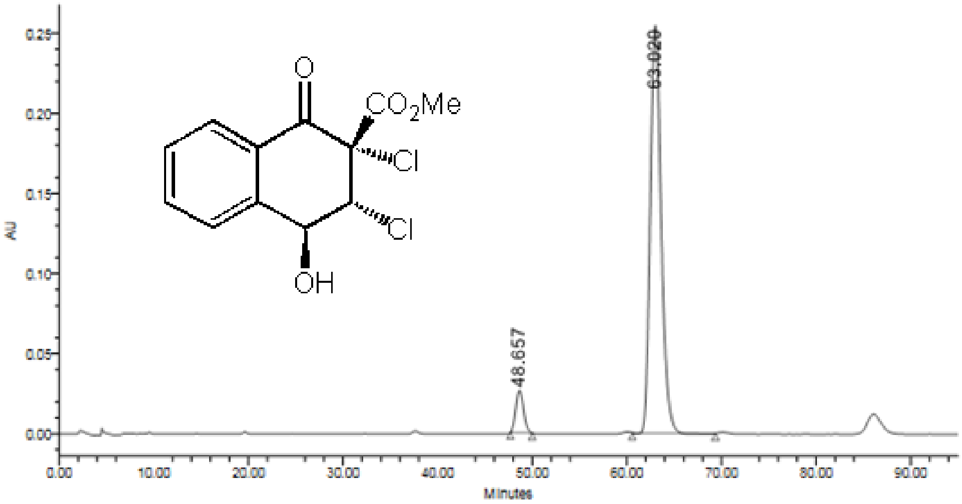

	RT (min)	Area (µV*sec)	% Area	Height (µV)	% Height
1	48.657	1513141	6.92	26451	9.42
2	63.020	20355824	93.08	254371	90.58
